# The prevalence of *EGFR* mutation in patients with non-small cell lung cancer: a systematic review and meta-analysis

**DOI:** 10.18632/oncotarget.12587

**Published:** 2016-10-12

**Authors:** Yue-Lun Zhang, Jin-Qiu Yuan, Kai-Feng Wang, Xiao-Hong Fu, Xiao-Ran Han, Diane Threapleton, Zu-Yao Yang, Chen Mao, Jin-Ling Tang

**Affiliations:** ^1^ Division of Epidemiology, The Jockey Club School of Public Health and Primary Care, The Chinese University of Hong Kong, Hong Kong, China; ^2^ Shenzhen Municipal Key Laboratory for Health Risk Analysis, Shenzhen Research Institute of The Chinese University of Hong Kong, Shenzhen, Guangdong Province, China; ^3^ Division of Epidemiology, School of Public Health and Tropical Medicine, Southern Medical University, Guangzhou, Guangdong Province, China

**Keywords:** non-small cell lung cancer, epidermal growth factor receptor, prevalence, systematic review, meta-analysis

## Abstract

**Objectives:**

Estimate the epidermal growth factor receptor (*EGFR*) mutation prevalence in all non-small cell lung cancer (NSCLC) patients and patient subgroups.

**Results:**

A total of 456 studies were included, reporting 30,466 patients with *EGFR* mutation among 115,815 NSCLC patients. The overall pooled prevalence for *EGFR* mutations was 32.3% (95% CI 30.9% to 33.7%), ranging from 38.4% (95% CI: 36.5% to 40.3%) in China to 14.1% (95% CI: 12.7% to 15.5%) in Europe. The pooled prevalence of *EGFR* mutation was higher in females (females vs. males: 43.7% vs. 24.0%; OR: 2.7, 95% CI: 2.5 to 2.9), non-smokers (non-smokers vs. past or current smokers: 49.3% vs. 21.5%; OR: 3.7, 95% CI: 3.4 to 4.0), and patients with adenocarcinoma (adenocarcinoma vs. non-adenocarcinoma: 38.0% vs. 11.7%; OR: 4.1, 95% CI: 3.6 to 4.8).

**Materials and Methods:**

PubMed, EMBASE, and the Cochrane Library were searched to June 2013. Eligible studies reported *EGFR* mutation prevalence and the association with at least one of the following factors: gender, smoking status and histology. Random-effects models were used to pool *EGFR* mutation prevalence data.

**Conclusion:**

This study provides the exact prevalence of *EGFR* mutations in different countries and NSCLC patient subgroups.

## INTRODUCTION

Lung cancer is the most common cause of cancer death, claiming 1.59 million lives in 2012 worldwide [1]. Non-small cell lung cancer (NSCLC) accounts for approximately 85% of all lung cancers and chemotherapy is one of the key components in the treatment protocol [2]. Although there is strong evidence to show that standard chemotherapy in addition to best supportive care can prolong overall survival and improve the quality of life [3], prognosis remains poor, especially in patients with advanced NSCLC. The median overall survival and the 5-year survival rate is only 1 year and 3.5%, respectively [4].

A large proportion of NSCLC patients have sensitizing mutations in exon 19 or 21 (approximately 45 and 40% of patients, respectively) which activate the tyrosine kinase domain in epidermal growth factor receptors (EGFR). The discovery of EGFR tyrosine kinase inhibitors (TKI) was regarded as a landmark finding in the treatment of lung cancer [5–8]. Targeted EGFR-TKI first-line treatment of sensitizing *EGFR* mutations results in longer PFS, improved health-related quality of life and decreased treatment-related severe side effects when compared with those who received standard chemotherapy [6, 7, 9–13]. Many clinical guidelines therefore recommend that all patients with sensitizing *EGFR* mutations receive first-line treatment with these drugs [14–19], in addition to all patients with advanced or metastatic NSCLC [2, 20].

Despite benefits in using EGFR-TKIs for first-line therapy in all sensitizing mutations, in resource limited situations, targeted screening may be appropriate [21]. Knowledge on the prevalence of *EGFR* mutations in different patient subgroups could therefore inform policy and testing strategies. Existing individual studies may not be informative for estimating prevalence because of small sample sizes in case-series or non-representative sample selection. The mutation prevalence reported in whole study or sub-group populations also varies dramatically in published studies [22–30] and the reported prevalence rates are therefore currently inappropriate for applying to other populations. In order to obtain a more precise estimate of *EGFR* mutation prevalence in NSCLC patients and patient subgroups, we conducted this systematic review and meta-analysis.

## RESULTS

**Figure 1:**
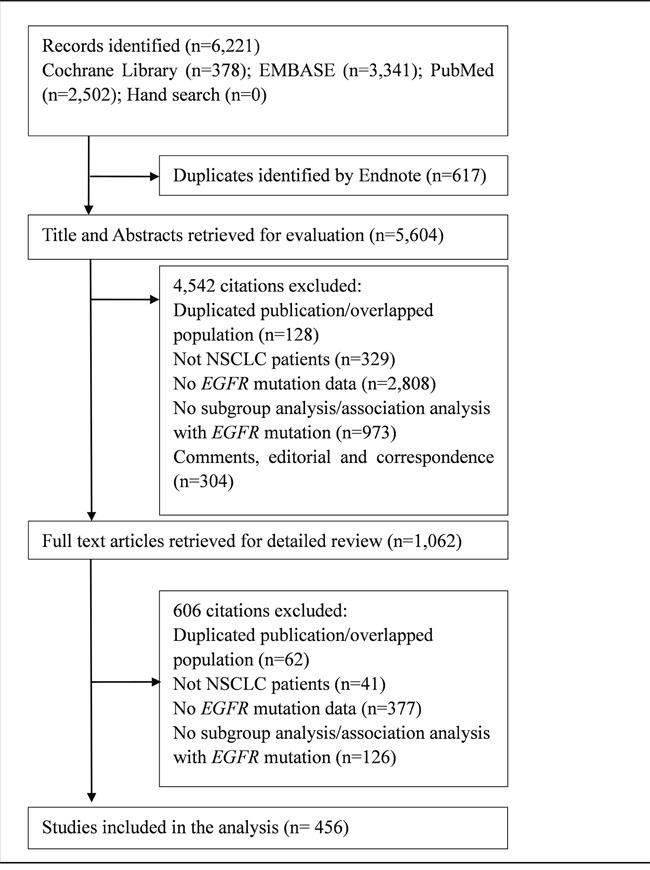
The flow chart of study selection This figure provides detailed information for the study inclusion and exclusion.

We identified 6,221 potentially eligible citations, of which 456 studies were finally included (Figure 1). The majority of included studies were case series (56.6%) and cohort studies (33.2%). Sixty-six percent of the studies were conducted in Asian countries. The median sample size was 103, ranging from 11 to 18,246. Median participant age was 63 years, 56.1% were males and 57.0% were past or current smokers. Histology was reported in 45.0% of studies and 73.3% of patients had adenocarcinoma. *EGFR* exon 19 or 21 mutation was assessed in 89.3% of the studies (see Table A.1 for study characteristics).

In total, 30,466 patients with an *EGFR* mutation were reported among 115,815 patients with NSCLC. The overall pooled prevalence of all *EGFR* mutations and *EGFR* exon 19 or 21 mutations was 32.3% (95% CI: 30.9% to 33.7%) and 32.2% (95% CI: 29.6% to 34.8%), respectively (Table 1). *EGFR* mutation prevalence varied by study location and ethnicity: Asia had the highest prevalence (38.4%, 95% CI: 36.5% to 40.3%) [China: 38.4% (95% CI: 35.7% to 41.1%); Japan: 36.6% (95% CI: 33.2% to 40.0%); Korea 32.4% (95% CI: 28.0% to 36.8%)], followed by North and South America (24.4%, 95% CI: 22.1% to 26.8%) and Europe (14.1%, 95% CI: 12.7% to 15.5%). The prevalence among different ethnicities were similar to study locations, with the prevalence of 38.8% (95% CI: 36.8% to 40.8%) in Asian populations, 17.4% (95% CI: 15.8% to 18.9%) in Caucasians, 17.2% (95% CI: 5.7% to 28.8%) in African-Americans, and 27.0% (95% CI: 22.6% to 31.4%) in mixed populations.

**Table 1:** The pooled prevalence of *EGFR* mutation in different NSCLC patient subgroups

Group variables	No. of studies	Mutation prevalence, % (95%CI)	Tests of heterogeneity	
			P	I^2^(%)
All studies	456	32.3(30.9 to 33.7)	<0.001	97.3
Exon mutation location				
Exon 19	343	16.7(15.8 to 17.5)	<0.001	93.2
Exon 21	330	12.3(11.6 to 13.0)	<0.001	92.7
Exon 19 or 21	95	32.2(29.6 to 34.8)	<0.001	95.5
Exon 18	90	1.2(1.0 to 1.4)	<0.001	48.6
Exon 20	93	1.7(1.4 to 2.0)	<0.001	64.2
Study location (continent)^*^				
Asia	304	38.4(36.5 to 40.3)	<0.001	95.6
America (North and South)	81	24.4(22.1 to 26.8)	<0.001	96.8
Europe	62	14.1(12.7 to 15.5)	<0.001	87.3
Study location (country)				
Japan	107	36.6(33.2 to 40.0)	<0.001	96.4
China	104	38.4(35.7 to 41.1)	<0.001	93.0
Korea	48	32.4(28.0 to 36.8)	<0.001	94.8
U.S.	68	23.9(21.3 to 26.5)	<0.001	96.6
Ethnicity				
Caucasian	96	17.4(15.8 to 18.9)	<0.001	92.0
Asian	301	38.8(36.8 to 40.8)	<0.001	95.8
African-American	5	17.2(5.7 to 28.8)	<0.001	89.0
Mixed	32	27.0(22.6 to 31.4)	<0.001	96.9
Unclear	22	19.3(16.4 to 22.2)	<0.001	94.3
Gender				
Male	322	24.0(22.5 to 25.4)	<0.001	94.2
Female	331	43.7(41.5 to 45.9)	<0.001	94.8
Smoking status				
Non-smoker	284	49.3(47.2 to 51.4)	<0.001	91.5
Past or current smoker	280	21.5(20.2 to 22.7)	<0.001	92.2
Histology				
Adenocarcinoma	307	38.0(36.0 to 40.1)	<0.001	96.6
Non-adenocarcinoma	203	11.7(10.6 to 12.7)	<0.001	83.6
Stage				
Stage I	73	34.0(28.9 to 39.1)	<0.001	97.4
Stage II	55	29.9(25.0 to 34.7)	<0.001	84.9
Stage III	85	33.8(29.8 to 37.8)	<0.001	89.0
Stage IV	68	37.5(33.2 to 41.7)	<0.001	93.3
Chemotherapy				
Chemotherapy	42	33.8(27.1 to 40.5)	<0.001	97.8
No chemotherapy	74	33.2(29.3 to 37.1)	<0.001	96.7

Legend: This table provides pooled prevalence and results from heterogeneity test of *EGFR* mutation in all eligible studies and different subgroups

* Europe includes countries of the European Union and Norway, Switzerland, and Turkey; America includes Canada, the United States, Argentina, Brazil, Colombia, Mexico, and Peru; Asia includes China, East Asia, India, Japan, Korea, Malaysia, Singapore, Taiwan, and Thailand.

Many study or patient characteristics had little influence on *EGFR* mutation prevalence rates, including the disease stage at diagnosis, history of chemotherapy, mutation detection methods (post-hoc analysis of methods reported in 10+ studies) or year of study publication (post-hoc analysis) (Table A.2 for post-hoc analysis).

Table 2 provides associations between of *EGFR* mutation prevalence and gender, smoking status, and tumor histology in Caucasian and Asian populations. Overall, prevalence was higher in females (females vs. males: 43.7% vs. 24.0%; OR: 2.7, 95% CI: 2.5 to 2.9) and the ORs did not differ significantly by ethnicity: Caucasian females vs. males OR 2.7, 95% CI 2.3 to 3.3; Asian females vs. males OR 2.8, 95% CI 2.6 to 3.1. The prevalence was also higher overall in non-smokers (non-smoker vs. past or current smoker: 49.3% vs. 21.5%; OR: 3.7, 95% CI: 3.4 to 4.0). Among non-smokers, as compared to past or current smokers, the mutation prevalence in Caucasians was greater than in Asians: Caucasian non-smokers vs. past or current smokers 39.8% vs. 10.8%; OR: 5.2, 95% CI: 4.4 to 6.3; Asian non-smokers vs. past or current smokers 52.2% vs. 26.3%; OR: 3.3, 95% CI: 2.9 to 3.6. NSCLC patients with adenocarcinoma were also far more likely to carry the *EGFR* mutation (adenocarcinoma vs. non-adenocarcinoma: 38.0% vs. 11.7%; OR: 4.1, 95% CI: 3.6 to 4.8) in overall participants. This observation was more striking in the Asian population (adenocarcinoma vs. non-adenocarcinoma: 44.7% vs. 12.5%; OR: 5.3, 95% CI: 4.4 to 6.4) than the Caucasian population (adenocarcinoma vs. non-adenocarcinoma: 19.7% vs. 9.6%; OR: 2.2, 95% CI: 1.7 to 2.7). The prevalence of *EGFR* mutation was not different in patients diagnosed at different NSCLC stages and by chemotherapy use history.

**Table 2:** The association of *EGFR* mutation with gender, smoking status, and tumor histology in Caucasian and Asian populations

Group variables	No. of studies	Mutation prevalence, 95%CI, (%)	Tests of heterogeneity		OR (95% CI)
			P	I^2^(%)	
**Caucasian population**					
Gender					
Female	66	25.0(22.4 to 27.7)	<0.001	87.2	2.7(2.3 to 3.3)
Male	62	10.1(8.6 to 11.5)	<0.001	86.1	1.0
Smoking status					
Non-smoker	56	39.8(36.3 to 43.2)	<0.001	76.8	5.2(4.4 to 6.3)
Past or current smoker	56	10.8(9.4 to 12.2)	<0.001	82.9	1.0
Histology					
Adenocarcinoma	61	19.7(17.5 to 21.8)	<0.001	89.6	2.2(1.7 to 2.7)
Non-adenocarcinoma	40	9.6(7.6 to 11.5)	<0.001	84.1	1.0
**Asian Population**					
Gender					
Female	231	51.1(48.9 to 53.3)	<0.001	88.0	2.8(2.6 to 3.1)
Male	228	28.7(26.7 to 30.6)	<0.001	92.6	1.0
Smoking status					
Non-smoker	197	52.2(49.7 to 54.7)	<0.001	91.5	3.3(2.9 to 3.6)
Past or current smoker	194	26.3(24.3 to 28.2)	<0.001	91.7	1.0
Histology					
Adenocarcinoma	214	44.7(42.4 to 47.0)	<0.001	93.8	5.3(4.4 to 6.4)
Non-adenocarcinoma	144	12.5(11.1 to 13.9)	<0.001	84.0	1.0

Legend: This table provides pooled prevalence and results from heterogeneity test of *EGFR* mutation, and the pooled within-study ORs for the association between subgroup variables and *EGFR* mutation, in Caucasian populations and Asian populations respectively.

The prevalence trends in different patient groups remained when countries with at least of 50 studies were assessed separately (China, Japan, Korea and U.S) (Table A.3 to A.6). Notable exceptions included a very low prevalence (3.3%, 95% CI: 2.4% to 4.1%) among patients with non-adenocarcinoma patients in Japan (Table A.4) and the higher prevalence for those who previously received chemotherapy in Japan (45.3%, 95% CI: 37.7% to 52.8%) and Korea (34.8%, 95% CI: 18.4% to 51.2%) (Tables A.4 and A.5).

## DISCUSSION

In this study, we found that approximately one-third of NSCLC patients harbor an *EGFR* mutation. Patients who are Asian, female, non-smokers, and have adenocarcinoma are more likely to harbor an *EGFR* mutation, which is consistent with previous studies [23, 29, 31–33].

Some previous systematic reviews were published reporting the prevalence of *EGFR* mutation in patients with non-small cell lung cancer [34–38]. These previously published work focused on patient subgroups such as smokers, adenocarcinomas or studies only in Chinese population. Moreover, all these previous reviews did not employ meta-analysis method to pool the prevalence from original studies. Despite of these difference, the pooled prevalence estimates we generated were similar to these smaller systematic reviews. The overall pooled prevalence in our study is similar to some existing large individual studies, though there is considerable variation among countries and individual studies may not be relied upon for accurate prevalence rates [39–41]. Individual study divergence from the pooled estimates likely reflects the patient characteristics within studies. *EGFR* mutation prevalence is clearly influenced by these characteristics and thus, this large review of whole populations and subgroups provides the best evidence for *EGFR* mutation prevalence [42, 43].

Recent clinical guidelines from the American Society of Clinical Oncology (ASCO) and the National Comprehensive Cancer Network (NCCN) recommend all patients with advanced or metastatic non-squamous NSCLC to receive *EGFR* mutation testing [2, 44]. A cost-effectiveness analysis also supports mutation testing vs. no testing for eligibility of second-line gefitinib treatment, after the failure of platinum-containing doublet [45]. However, this may require substantial resources and effort and at present, the cost of *EGFR* mutation testing in some countries is met by patients' out-of-pocket expenses or from research funds, and can vary from U.S.$ 150 to several hundred U.S.$ [46]. Although non-invasive genetic testing methods exist [47], mutation detection using tumor tissues is still the gold standard for *EGFR* mutation testing. Such barriers prevent the widespread use of *EGFR* mutation testing and testing rates likely reflect the level of health service development and insurance coverage. *EGFR* mutation testing rates in NSCLC vary considerably among countries with approximately 9.6% in Chinese metropolitan areas in 2010 [48] and approximately 39.5% and 53.9% of patients in Korea and Japan, respectively [49, 50]. Where full coverage of screening is not available or possible, providers may target the patient groups identified in this review who are more likely to harbor mutations (e.g. 51.5% of Asian females, 52.2% of Asian smokers, 44.7% of Asian patients with adenocarcinoma, and 39.8% of Caucasian non-smokers harbor an *EGFR* mutation). Although *EGFR* mutation prevalence in some populations is relatively low at about 10% (e.g. 10.1% in Caucasian males and 12.5% in Asian patients with non-adenocarcinoma) screening must still be provided and only be targeted in resource limited settings. Given the fact that the efficacy of standard chemotherapy for patients with NSCLC remains limited, universal testing for *EGFR* mutation may improve overall prognosis through the early use of first-line EGFR-TKI treatment.

A major strength of this work is the large number and range of studies included to estimate the prevalence of *EGFR* mutation in different NSCLC patient groups. These estimates can serve as the reference for the future research or policy making. However, several limitations of our study need to be mentioned. Firstly, since patients with higher likelihood of harboring an *EGFR* mutation are more likely to be tested, the overall prevalence may be overestimated. However, detailed recruitment information was not provided in many studies so we could not assess the influence of selection bias. Secondly, lack of relevant studies prevented us from further exploring the prevalence of *EGFR* mutation in some patient subgroups, such as Asian female non-smokers and Asian female adenocarcinoma patients. Thirdly, significant heterogeneity was found among included studies in almost all the analyses, which seems common in meta-analysis of single arm studies. The substantial difference in patient characteristics, clinical settings, and research methodologies among eligible studies may contribute to the high level of heterogeneity. Unfortunately, without individual patient data it is not possible to further sub-divide prevalence estimates for precise patient groups such as women in the U.S. who are non-smokers and have adenocarcinoma. Despite noted limitations, we believe that this comprehensive synthesis of existing available studies provides valuable estimates of *EGFR* mutation prevalence.

This systematic review and meta-analysis estimates the prevalence of *EGFR* mutations in NSCLC patient subgroups. *EGFR* mutation prevalence estimates in these subgroups can inform policy makers of those patients who are more likely to benefit from EGFR-TKI treatment.

## MATERIALS AND METHODS

### Data sources and search strategy

We performed a comprehensive literature search in PubMed, EMBASE, and Cochrane Library from their respective inception to June 2013, with different combinations of the following keywords: “EGFR”, “lung cancer”, and “mutation”. The literature search was restricted to human studies. No language restriction was applied. We manually checked reference lists of the included studies and relevant reviews to identify additional studies. Details of the search strategy are summarized in Table A.7.

### Study selection

Studies reporting the prevalence of *EGFR* mutation and odds ratio (OR) to estimate the association of *EGFR* mutation with at least one of the following factors (gender, smoking status, and histology) in patients with NSCLC were considered eligible. For duplicate publications, we selected the most recent and complete version of publications. Two reviewers (WKF and YJQ) independently assessed the study eligibility. Any discrepancies were resolved by consensus or by consulting with a third reviewer (MC).

### Data extraction

Data extraction was performed independently by two reviewers (WKF and YJQ). Disagreements were resolved by discussion between the two. If no agreement was reached, then a third researcher (MC) was invited to resolve the disagreement. The following data were collected from each study using a predefined data extraction form: study characteristics (such as the first author's name, year of publication, study location, study design, and sample size), patient characteristics (such as proportion of females, proportion of non-smokers, and proportion of adenocarcinoma), and the prevalence of *EGFR* mutation (such as the number of patients with *EGFR* mutation and OR for association of *EGFR* mutation with gender, smoking status, and histology).

### Outcome measure and subgroup variables

We defined the primary outcome of interest in this systematic review as *EGFR* mutation. The prevalence of *EGFR* mutation was defined as the proportion of patients with *EGFR* mutation among patients who received the mutation testing. Study location was determined using the place where the patients were recruited in the study. If the authors did not describe the patient recruitment, the affiliation address of the corresponding author was used to identify the study location. Population ethnicity was divided into Caucasian, Asian, African-American, and mixed ethnicities. Ethnicity classification was based on the information in the original publication or the principle ethnicity of each country, where the detailed information was not reported (e.g. Italy = Caucasian, Japan = Asian). Similarly, the classifications of smoking status, tumor histology, tumor stage, and previous use of chemotherapy were defined according to the information in original publications.

### Data analysis

We conducted meta-analysis to pool the prevalence of *EGFR* mutation in different NSCLC patient groups. Subgroup analyses were performed according to the following factors: *EGFR* exons (exon 19, 21 or both, and the others), study locations (at least 50 studies in each location), ethnicity, gender, smoking status (non-smoker or past or current smoker), tumor histology (adenocarcinoma or non-adenocarcinoma), tumor stage, and previous use of chemotherapy. The within-study OR was combined to estimate the association of *EGFR* mutation with gender, smoking status, and tumor histology. Post-hoc subgroup analysis was conducted according to the detection methods of *EGFR* mutation, types of samples used in the detection, and the publication year of the study. Given the diverse nature of studies and the likely heterogeneity, we applied random-effects models to carry out meta-analysis by the Der-Simonian Laird method [51]. The statistical heterogeneity among the studies was assessed by the Cochran's Q-test and the I^2^ statistic. A P value ≤ 0.10 for the Cochran's Q-test or an I^2^ ≥ 50% was suggestive of significant among-study heterogeneity. We used Stata 12.0 (StataCorp, College Station, TX) to conduct all the analyses with a two-tailed significance level of 0.05 except in the assessment of heterogeneity (α = 0.10).

## SUPPLEMENTARY FIGURES AND TABLES




