# Supplementary material for: The prevalence of EGFR mutation in patients with non-small cell lung cancer: a systematic review and meta-analysis
Source: Oncotarget. 2016 Oct 12;7(48):78985–93. doi: 10.18632/oncotarget.12587 (PMC5346692; doi:10.18632/oncotarget.12587)
Supplement: Supplementary file 1 [file oncotarget-07-78985-s001.pdf]

The prevalence of *EGFR* mutation in patients with non-small cell lung cancer: a systematic review and meta-analysis

APPENDIX TABLES

Table A.1: The basic characteristics of included studies

See Appendix File 1

Table A.2: Post-hoc subgroup analysis on the detection methods, samples for EGFR mutation, and publication year

Group variables	No. of studies	Mutation prevalence, 95% CI (%)	Tests of heterogeneity	
			P	I^2(%)
All studies	456	32.3 (30.9 to 33.7)	<0.001	97.3
Detection method				
Pyrosequencing or direct sequencing	306	32.2 (30.3 to 34.0)	<0.001	97.4
COLD-PCR and pyrosequencing or fragment size analysis	7	31.9 (24.5 to 39.3)	<0.001	85.8
Sanger sequencing	2	35.5 (27.2 to 43.7)	0.205	37.8
Amplification refractory mutation system ARMS	28	33.7 (27.1 to 40.4)	<0.001	97.8
Real-time PCR	13	27.6 (20.2 to 35.1)	<0.001	96.9
Fragment size analysis	4	42.5 (29.0 to 56.1)	<0.001	89.5
RT-PCR	7	41.6 (23.6 to 59.5)	<0.001	97.0
PCR and sequencing	26	28.9 (24.9 to 32.9)	<0.001	95.7
PCR single strand conformational polymorphism	3	43.0 (0.0 to 93.1)	<0.001	99.5
High Performance Liquid Chromatography	11	37.7 (32.7 to 42.7)	<0.001	70.8
PCR clamp method or PCR invader assay	5	52.9 (36.2 to 69.7)	<0.001	93.3
Peptide nucleic acid (PNA) clamping	21	33.4 (28.1 to 38.8)	<0.001	91.3
Other methods	2	14.1 (10.4 to 17.7)	0.931	0.0
Unclear	21	25.0 (20.9 to 29.1)	<0.001	96.2
Sample for *EGFR* mutation detection				
Tissue	365	32.8 (31.1 to 34.5)	<0.001	97.0
Blood	10	36.0 (26.4 to 45.6)	<0.001	88.3
Mixed (at least include tissue or blood samples)	41	31.9 (26.8 to 37.0)	<0.001	97.5
Other samples	11	25.1 (14.2 to 36.1)	<0.001	94.9
Unclear	29	27.8 (24.1 to 31.5)	<0.001	97.7
Publication date				
2004	5	38.3 (20.9 to 55.7)	<0.001	96.0
2005	36	26.6 (22.0 to 31.2)	<0.001	94.8
2006	29	29.2 (24.7 to 33.7)	<0.001	87.3
2007	38	30.6 (24.8 to 36.4)	<0.001	96.5
2008	37	34.2 (28.3 to 40.1)	<0.001	95.8
2009	37	30.0 (25.0 to 35.1)	<0.001	96.6
2010	60	35.3 (31.0 to 39.6)	<0.001	96.9
2011	107	34.9 (31.5 to 38.4)	<0.001	97.9
2012	89	31.3 (28.5 to 34.2)	<0.001	97.9
2013	18	31.2 (24.1 to 38.3)	<0.001	97.6

Table A.3: The pooled prevalence of *EGFR* mutation in studies from China

Group variables	No. of studies	Mutation prevalence, 95%CI (%)	Tests of heterogeneity	
			P	I^2 (%)
All studies	104	38.4(35.7 to 41.1)	<0.001	93.0
Exons				
Exon 19	86	19.9(18.0 to 21.9)	<0.001	88.8
Exon 21	84	15.8(14.3 to 17.4)	<0.001	83.2
Exon 19 or 21	34	35.8(31.4 to 40.2)	<0.001	91.9
Gender				
Female	82	50.3(46.9 to 53.7)	<0.001	84.8
Male	82	30.3(27.5 to 33.2)	<0.001	88.0
Smoking status				
Non-smoker	69	49.0(45.3 to 52.7)	<0.001	89.4
Past or current smoker	65	27.6(24.2 to 31.0)	<0.001	90.0
Histology				
Adenocarcinoma	82	50.3(46.9 to 53.7)	<0.001	84.8
Non-adenocarcinoma	60	16.9(14.3 to 19.5)	<0.001	81.4
Chemotherapy				
Chemotherapy	15	37.4(29.0 to 45.9)	<0.001	90.2
No chemotherapy	25	36.3(30.3 to 42.3)	<0.001	93.4

Table A.4: The Pooled prevalence of *EGFR* mutation in studies from Japan

Group variables	No. of studies	Mutation prevalence, 95% CI (%)	Tests of heterogeneity	
			P	I^2(%)
All studies	107	36.6(33.2 to 40.0)	<0.001	96.4
Exons				
Exon 19	81	17.8(15.9 to 19.7)	<0.001	87.8
Exon 21	79	15.9(14.2 to 17.5)	<0.001	83.2
Exon 19 or 21	27	35.3(30.6 to 39.9)	<0.001	93.1
Gender				
Female	81	49.8(45.3 to 54.2)	<0.001	91.8
Male	81	22.5(19.7 to 25.3)	<0.001	91.5
Smoking status				
Non-smoker	66	54.4(48.6 to 60.1)	<0.001	94.7
Past or current smoker	67	20.6(17.9 to 23.4)	<0.001	90.8
Histology				
Adenocarcinoma	76	41.6(37.2 to 46.0)	<0.001	95.7
Non-adenocarcinoma	42	3.3(2.4 to 4.1)	0.068	25.8
Chemotherapy				
Chemotherapy	5	45.3(37.7 to 52.8)	0.672	0.0
No chemotherapy	17	36.5(27.3 to 45.8)	<0.001	92.1

Table A.5: The pooled prevalence of *EGFR* mutation in studies from Korea

Group variables	No. of studies	Mutation prevalence, 95% CI (%)	Tests of heterogeneity	
			P	I^2(%)
All studies	48	32.4(28.0 to 36.8)	<0.001	94.8
Exons				
Exon 19	37	17.8(15.0 to 20.6)	<0.001	88.4
Exon 21	37	10.2(8.3 to 12.1)	<0.001	82.9
Exon 19 or 21	4	37.9(25.4 to 50.5)	<0.001	89.9
Gender				
Female	38	47.5(43.2 to 51.7)	<0.001	80.5
Male	35	24.7(20.2 to 29.2)	<0.001	90.7
Histology				
Adenocarcinoma	33	40.6(36.2 to 45.0)	<0.001	89.2
Non-adenocarcinoma	25	14.4(10.5 to 18.3)	<0.001	81.4
Smoking status				
Non-smoker	35	47.2(42.9 to 51.5)	<0.001	80.0
Past or current smoker	35	23.9(19.7 to 28.2)	<0.001	88.6
Chemotherapy				
Chemotherapy	8	34.8(18.4 to 51.2)	<0.001	97.2
No chemotherapy	5	19.8(16.4 to 23.3)	0.878	0.0

Table A.6: The pooled prevalence of *EGFR* mutation in studies from U.S.

Group variables	No. of studies	Mutation prevalence, 95% CI (%)	Tests of heterogeneity	
			P	I^2(%)
All studies	68	23.9(21.3 to 26.5)	<0.001	96.6
Exons				
Exon 19	37	12.8(11.0 to 14.6)	<0.001	92.9
Exon 21	34	7.6(6.3 to 9.0)	<0.001	92.0
Exon 19 or 21	19	25.0(21.5 to 28.5)	<0.001	92.2
Gender				
Female	42	29.3(25.4 to 33.2)	<0.001	93.4
Male	38	18.9(15.7 to 22.2)	<0.001	89.7
Smoking status				
Non-smoker	42	46.5(41.3 to 51.6)	<0.001	91.8
Past or current smoker	40	14.3(12.5 to 16.1)	<0.001	83.2
Histology				
Adenocarcinoma	42	25.3(22.7 to 27.9)	<0.001	90.1
Non-adenocarcinoma	21	17.3(12.7 to 21.9)	<0.001	79.6
Chemotherapy				
Chemotherapy	5	19.1(6.2 to 31.9)	<0.001	96.9
No chemotherapy	7	19.8(13.5 to 26.2)	<0.001	92.8

Table A.7: Search strategy

Search	Query
1	EGFR
2	lung cancer
3	1 and 2
4	mutation or mutant or mutated
5	3 and 4

LIST OF ELIGIBLE STUDIES

1. Fan M, Li H, Lü Z, Zhang H, Shen X. [Relationship of epidermal growth factor receptor gene mutations, clinicopathologic features and prognosis in patients with non-small cell lung cancer]. Chinese journal of pathology. 2011; 40:679-682.
2. Fang Q, Zhang L, Wang S, Ou W. [Discordance of epidermal growth factor receptor mutations between primary and corresponding metastatic tumors in non-small cell lung cancer]. Chinese journal of lung cancer. 2011; 14:518-522.
3. Feng Q, Li X, Chen Z, He J, Wang C, Zhou L, Xue W. [Epidermal growth factor receptor gene mutations and clinicopathologic correlation in 309 patients with non-small cell lung cancer]. Chinese journal of pathology. 2011; 40:660-663.
4. Fiala O, Pesek M, Finek J, Bruha F, Bortlicek Z, Krejci J, Benesova L, Minarik M. EGFR mutations in patients with advanced NSCLC. Lung cancer (Amsterdam, Netherlands). 2012; 77:S30.
5. Fong Y, LIN YS, LIOU CP, LI CF, TZENG CC. Chromosomal imbalances in lung adenocarcinomas with or without mutations in the epidermal growth factor receptor gene. Respirology (Carlton, Vic). 2010; 15:700-705.
6. Fujimoto D, Tomii K, Otoshi T, Kawamura T, Tamai K, Takeshita J, Tanaka K, Matsumoto T, Monden K, Nagata K. Preexisting interstitial lung disease is inversely correlated to tumor epidermal growth factor receptor mutation in patients with lung adenocarcinoma. Lung cancer (Amsterdam, Netherlands). 2013; 80:159-164.
7. Fujita S, Katakami N, Masago K, Yoshioka H, Tomii K, Kaneda T, Hirabayashi M, Kunimasa K, Morizane T, Mio T. Customized chemotherapy based on epidermal growth factor receptor mutation status for elderly patients with advanced non-small-cell lung cancer: a phase II trial. BMC cancer. 2012; 12:1.
8. Fujiwara Y, Kiura K, Toyooka S, Takigawa N, Tokumo M, Hotta K, Aoe M, Tabata M, Matsuo K, Date H. Relationship between epidermal growth factor receptor gene mutations and the severity of adverse events by gefitinib in patients with advanced non-small cell lung cancer. Lung cancer (Amsterdam, Netherlands). 2006; 52:99-103.
9. Fukuoka M, Wu Y-L, Thongprasert S, Sunpaweravong P, Leong S-S, Sriuranpong V, Chao T-Y, Nakagawa K, Chu D-T, Saijo N. Biomarker analyses and final overall survival results from a phase III, randomized, open-label, first-line study of gefitinib versus carboplatin/paclitaxel in clinically selected patients with advanced non–small-cell lung cancer in Asia (IPASS). Journal of Clinical Oncology. 2011; 29:2866-2874.
10. Gandara DR, Grimminger P, Mack PC, Lara PN, Li T, Danenberg PV, Danenberg KD. Association of epidermal growth factor receptor activating mutations with low ERCC1 gene expression in non-small cell lung cancer. Journal of Thoracic Oncology. 2010; 5:1933-1938.
11. Gao B, Sun Y, Zhang J, Ren Y, Fang R, Han X, Shen L, Liu X-y, Pao W, Chen H. Spectrum of LKB1, EGFR, and KRAS mutations in chinese lung adenocarcinomas. Journal of Thoracic Oncology. 2010; 5:1130-1135.
12. Gao J, Chen J, Zhang L, Liang Z. [Relationship between EGFR and KRAS mutations and prognosis in Chinese patients with non-small cell lung cancer: a mutation analysis with real-time polymerase chain reaction using scorpion amplification refractory mutation system]. Chinese journal of pathology. 2012; 41:652-656.
13. Garcia-Olivé I, Monsó E, Andreo F, Sanz-Santos J, Taron M, Molina-Vila M, Llatjós M, Castellà E, Moran T, Bertran-Alamillo J. Endobronchial ultrasound-guided transbronchial needle aspiration for identifying EGFR mutations. European Respiratory Journal. 2010; 35:391-395.
14. Giovannetti E, Zucali PA, Peters GJ, Cortesi F, D'Incecco A, Smit EF, Falcone A, Burgers JA, Santoro A, Danesi R. Association of Polymorphisms in AKT1 and EGFR with Clinical Outcome and Toxicity in Non–Small Cell Lung Cancer Patients Treated with Gefitinib. Molecular cancer therapeutics. 2010; 9:581-593.
15. Girard N, Lou E, Azzoli CG, Reddy R, Robson M, Harlan M, Orlow I, Yatabe Y, Nafa K, Ladanyi M. Analysis of genetic variants in never-smokers with lung cancer facilitated by an Internet-based blood collection protocol: a preliminary report. Clinical Cancer Research. 2010; 16:755-763.
16. Girard N, Sima C, Jackman DM, Sequist LV, Chen H, Yang J-H, Ji H, Waltman B, Rosell R, Taron M. Nomogram to predict the presence of EGFR activating mutation in lung adenocarcinoma. European Respiratory Journal. 2012; 39:366-372.
17. Goto K, Ichinose Y, Ohe Y, Yamamoto N, Negoro S, Nishio K, Itoh Y, Jiang H, Duffield E, McCormack R. Epidermal growth factor receptor mutation status in circulating free DNA in serum: from IPASS, a phase III study of gefitinib or carboplatin/paclitaxel in non-small cell lung cancer. Journal of Thoracic Oncology. 2012; 7:115-121.
18. Gow C-H, Chang Y-L, Hsu Y-C, Tsai M-F, Wu C-T, Yu C-J, Yang C-H, Lee Y-C, Yang P-C, Shih J-Y. Comparison of epidermal growth factor receptor mutations between primary and corresponding metastatic tumors in tyrosine kinase inhibitor-naive non-small-cell lung cancer. Annals of oncology. 2008:mdn679.
19. Bai H, Wang Z, Chen K, Zhao J, Lee JJ, Wang S, Zhou Q, Zhuo M, Mao L, An T, Duan J, Yang L, Wu M, Liang Z, Wang Y, Kang X, et al. (2012). Influence of chemotherapy on EGFR mutation status among patients

with non-small-cell lung cancer. (United States: American Society of Clinical Oncology (330 John Carlyle Street, Suite 300, Alexandria VA 22314, United States)), pp. 3077-3083.

20. Girard N, Sima CS, Jackman DM, Sequist LV, Chen H, Yang JC, Ji H, Waltman B, Rosell R, Taron M, Zakowski MF, Ladanyi M, Riely G, Pao W. Nomogram to predict the presence of EGFR activating mutation in lung adenocarcinoma. The European respiratory journal. 39:366-372.
21. Enting D, Januszewski A, Benepal T. EGFR-mutation analysis, treatment and outcomes in newly diagnosed patients with non-small cell lung cancer (NSCLC): A single-institution retrospective review. Elsevier Ireland Ltd), pp. S5.
22. Arrieta O, Cardona AF, Federico Bramuglia G, Gallo A, Campos-Parra AD, Serrano S, Castro M, Aviles A, Amorin E, Kirchuk R, Cuello M, Borbolla J, Riemersma O, Becerra H, Rosell R, ClicaP. Genotyping non-small cell lung cancer (NSCLC) in Latin America. Journal of thoracic oncology: official publication of the International Association for the Study of Lung Cancer. 6:1955-1959.
23. Wang F, Fu S, Tang T, Deng L, Zhang X, Li Y, Shao J. [Relationship between mutations of epidermal growth factor receptor gene and clinicopathologic features of non-small cell lung cancers]. Chinese journal of pathology. 2011; 40:664-666.
24. Jian G, Songwen Z, Ling Z, Qinfang D, Jie Z, Liang T, Caicun Z. Prediction of epidermal growth factor receptor mutations in the plasma/pleural effusion to efficacy of gefitinib treatment in advanced non-small cell lung cancer. Journal of cancer research and clinical oncology. 2010; 136:1341-1347.
25. Guo H, Wan Y, Tian G, Liu Q, Kang Y, Li Y, Yao Z, Lin D. EGFR mutations predict a favorable outcome for malignant pleural effusion of lung adenocarcinoma with Tarceva therapy. Oncology reports. 2012; 27:880.
26. Gwak G-Y, Yoon J-H, Shin C, Ahn Y, Chung J, Kim Y, Kim T-Y, Lee H-S. Detection of response-predicting mutations in the kinase domain of the epidermal growth factor receptor gene in cholangiocarcinomas. Journal of cancer research and clinical oncology. 2005; 131:649-652.
27. Han S-W, Kim T-Y, Hwang PG, Jeong S, Kim J, Choi IS, Oh D-Y, Kim JH, Kim D-W, Chung DH. Predictive and prognostic impact of epidermal growth factor receptor mutation in non–small-cell lung cancer patients treated with gefitinib. Journal of clinical oncology. 2005; 23:2493-2501.
28. Han Y, Xu J, Duan H, Song S, Liu X, Zhang Y, Zhang J. [EGFR mutation predicts response and prognosis in iressa-treated advanced-stage non-small cell lung cancer]. Chinese journal of oncology. 2007; 29:278-283.
29. Han R, Zhong W, Zhao J, Zhang L, Xia Y, Wang H, Li L, Wang M. [Comparison of EGFR mutation status in paired pre-and post-chemotherapy serum for advanced pulmonary adenocarcinoma]. Chinese journal of lung cancer. 2011; 14:127-131.
30. Han H-S, Eom D-W, Kim JH, Kim K-H, Shin H-M, An JY, Lee KM, Choe KH, Lee KH, Kim ST. EGFR mutation status in primary lung adenocarcinomas and corresponding metastatic lesions: discordance in pleural metastases. Clinical lung cancer. 2011; 12:380-386.
31. Han H-S, Lim S-n, An JY, Lee KM, Choe KH, Lee KH, Kim ST, Son S-M, Choi S-Y, Lee H-c. Detection of EGFR mutation status in lung adenocarcinoma specimens with different proportions of tumor cells using two methods of differential sensitivity. Journal of Thoracic Oncology. 2012; 7:355-364.
32. Han S-W, Kim T-Y, Jeon YK, Hwang PG, Im S-A, Lee K-H, Kim JH, Kim D-W, Heo DS, Kim NK. Optimization of patient selection for gefitinib in non–small cell lung cancer by combined analysis of epidermal growth factor receptor mutation, K-ras mutation, and Akt phosphorylation. Clinical Cancer Research. 2006; 12:2538-2544.
33. Han J-Y, Kim JY, Lee S-H, Lee GK, Lee JS. Association of plasma hepatocyte growth factor (HGF) and insulin-like growth factor binding protein (IGFBP)-3 with gefitinib resistance in patients with advanced non-small cell lung cancer. Cancer research. 2011; 71:4119-4119.
34. Haneda H, Sasaki H, Lindeman N, Kawano O, Endo K, Suzuki E, Shimizu S, Yukiue H, Kobayashi Y, Yano M. A correlation between EGFR gene mutation status and bronchioloalveolar carcinoma features in Japanese patients with adenocarcinoma. Japanese journal of clinical oncology. 2006; 36:69-75.
35. Harada T, Lopez-Chavez A, Xi L, Raffeld M, Wang Y, Giaccone G. Characterization of epidermal growth factor receptor mutations in non-small-cell lung cancer patients of African-American ancestry. Oncogene. 2011; 30:1744-1752.
36. Han SW, Kim TY, Yoon KJ, Pil GH, Im SA, Lee KH, Jee HK, Kim DW, Dae SH, Noe KK, Doo HC, Bang YJ. (2006). Optimization of patient selection for gefitinib in non-small cell lung cancer by combined analysis of epidermal growth factor receptor mutation, K-ras mutation, and Akt phosphorylation. (United States: American Association for Cancer Research Inc.), pp. 2528-2544.
37. Hata A, Katakami N, Fujita S, Kaji R, Imai Y, Takahashi Y, Nishimura T, Tomii K, Ishihara K. Frequency of EGFR and KRAS mutations in Japanese patients with lung adenocarcinoma with features of the mucinous subtype of bronchioloalveolar carcinoma. Journal of Thoracic Oncology. 2010; 5:1197-1200.
38. Hata A, Yoshioka H, Fujita S, Kunimasa K, Kaji R, Imai Y, Tomii K, Iwasaku M, Nishiyama A, Ishida T. Complex mutations in the epidermal growth factor receptor gene in non-small cell lung cancer. Journal of Thoracic Oncology. 2010; 5:1524-1528.

39. Hata A, Katakami N, Yoshioka H, Kunimasa K, Fujita S, Kaji R, Notohara K, Imai Y, Tachikawa R, Tomii K. How sensitive are epidermal growth factor receptor–tyrosine kinase inhibitors for squamous cell carcinoma of the lung harboring EGFR gene–sensitive mutations? Journal of Thoracic Oncology. 2013; 8:89-95.
40. He C, Liu M, Zhou C, Zhang J, Ouyang M, Zhong N, Xu J. Detection of epidermal growth factor receptor mutations in plasma by mutant-enriched PCR assay for prediction of the response to gefitinib in patients with non-small-cell lung cancer. International Journal of Cancer. 2009; 125:2393-2399.
41. He Y, Li S, Ren S, Cai W, Li X, Zhao C, Li J, Chen X, Gao G, Li W. Impact of family history of cancer on the incidence of mutation in epidermal growth factor receptor gene in non-small cell lung cancer patients. Lung cancer (Amsterdam, Netherlands). 2013; 81:162-166.
42. Helland Å, Skaug HM, Kleinberg L, Iversen ML, Rud AK, Fleischer T, Sagerup C, Solberg S, Jørgensen L, Ariansen S. EGFR gene alterations in a Norwegian cohort of lung cancer patients selected for surgery. Journal of Thoracic Oncology. 2011; 6:947-950.
43. Hirsch F, Varella-Garcia M, Cappuzzo F, McCoy J, Bemis L, Xavier A, Dziadziuszko R, Gumerlock P, Chansky K, West H. Combination of EGFR gene copy number and protein expression predicts outcome for advanced non-small-cell lung cancer patients treated with gefitinib. Annals of Oncology. 2007; 18:752-760.
44. Hlinkova K, Babal P, Berzinec P, Majer I, Mikle-Barathova Z, Piackova B, Ilencikova D. Evaluation of 2-year experience with EGFR mutation analysis of small diagnostic samples. Diagnostic Molecular Pathology. 2013; 22:70-75.
45. Horiike A, Kimura H, Nishio K, Ohyanagi F, Satoh Y, Okumura S, Ishikawa Y, Nakagawa K, Horai T, Nishio M. Detection of epidermal growth factor receptor mutation in transbronchial needle aspirates of non-small cell lung cancer. CHEST Journal. 2007; 131:1628-1634.
46. Hosokawa S, Toyooka S, Fujiwara Y, Tokumo M, Soh J, Takigawa N, Hotta K, Yoshino T, Date H, Tanimoto M. Comprehensive analysis of EGFR signaling pathways in Japanese patients with non-small cell lung cancer. Lung cancer (Amsterdam, Netherlands). 2009; 66:107-113.
47. Hotta K, Kiura K, Toyooka S, Takigawa N, Soh J, Fujiwara Y, Tabata M, Date H, Tanimoto M. Clinical significance of epidermal growth factor receptor gene mutations on treatment outcome after first-line cytotoxic chemotherapy in Japanese patients with non-small cell lung cancer. Journal of Thoracic Oncology. 2007; 2:632-637.
48. Hou X-W, Zhuang X-J, Song Q, Li L-J, Wang N-N. Application of CT-guided percutaneous lung biopsy in detecting epidermal growth factor receptor gene mutations in patients with advanced non-small cell lung cancer. Journal of Interventional Radiology. 2013; 22.
49. Hsieh RK, Lim KH, Kuo HT, Tzen CY, Huang MJ. (2005). Female sex and bronchioloalveolar pathologic subtype predict EGFR mutations in non-small cell lung cancer. (United States: American College of Chest Physicians), pp. 317-321.
50. Hsieh M-H, Fang Y-F, Chang W-C, Kuo H-P, Lin S-Y, Liu H-P, Liu C-L, Chen H-C, Ku Y-C, Chen Y-T. Complex mutation patterns of epidermal growth factor receptor gene associated with variable responses to gefitinib treatment in patients with non-small cell lung cancer. Lung cancer (Amsterdam, Netherlands). 2006; 53:311-322.
51. Hsu K-H, Chen K-C, Yang T-Y, Yeh Y-C, Chou T-Y, Chen H-Y, Tsai C-R, Chen C-Y, Hsu C-P, Hsia J-Y. Epidermal growth factor receptor mutation status in stage I lung adenocarcinoma with different image patterns. Journal of Thoracic Oncology. 2011; 6:1066-1072.
52. Hsu C-L, Chen K-Y, Shih J-Y, Ho C-C, Yang C-H, Yu C-J, Yang P-C. Advanced non-small cell lung cancer in patients aged 45 years or younger: outcomes and prognostic factors. BMC cancer. 2012; 12:1.
53. Huang S-F, Liu H-P, Li L-H, Ku Y-C, Fu Y-N, Tsai H-Y, Chen Y-T, Lin Y-F, Chang W-C, Kuo H-P. High frequency of epidermal growth factor receptor mutations with complex patterns in non–small cell lung cancers related to gefitinib responsiveness in Taiwan. Clinical Cancer Research. 2004; 10:8195-8203.
54. Huang J, Dong Q, Xu K, HAN B-h, BAI H, GENG X, ZHOU J. Epidermal growth factor receptor mutation in serum circulating DNA and selective targeting therapy against lung cancer. Tumor. 2007; 27:968-972.
55. Huang C-T, Yen R-F, Cheng M-F, Hsu Y-C, Wei P-F, Tsai Y-J, Tsai M-F, Shih J-Y, Yang C-H, Yang P-C. Correlation of F-18 fluorodeoxyglucose-positron emission tomography maximal standardized uptake value and EGFR mutations in advanced lung adenocarcinoma. Medical Oncology. 2010; 27:9-15.
56. Huang Y-s, Yang J-j, Zhang X-c, Yang X-n, Huang Y-j, Xu C-r, Zhou Q, Wang Z, Su J, Wu Y. Impact of smoking status and pathologic type on epidermal growth factor receptor mutations in lung cancer. Chinese Medical Journal-Beijing. 2011; 124:2457.
57. Huang Z, Wang Z, Bai H, Wu M, An T, Zhao J, Yang L, Duan J, Zhuo M, Wang Y. The detection of EGFR mutation status in plasma is reproducible and can dynamically predict the efficacy of EGFR-TKI. Thoracic Cancer. 2012; 3:334-340.
58. Hung M, Lin C, Leu S, Wu M, Tsai Y, Yang C. Epidermal growth factor receptor mutations in cells from non-small cell lung cancer malignant pleural effusions. Chang Gung medical journal. 2006; 29:373.

59. Inoue A, Suzuki T, Fukuhara T, Maemondo M, Kimura Y, Morikawa N, Watanabe H, Saijo Y, Nukiwa T. Prospective phase II study of gefitinib for chemotherapy-naive patients with advanced non–small-cell lung cancer with epidermal growth factor receptor gene mutations. Journal of clinical oncology. 2006; 24:3340-3346.
60. Jackman DM, Yeap BY, Lindeman NI, Fidias P, Rabin MS, Temel J, Skarin AT, Meyerson M, Holmes AJ, Borras AM. Phase II clinical trial of chemotherapy-naive patients≥ 70 years of age treated with erlotinib for advanced non–small-cell lung cancer. Journal of Clinical Oncology. 2007; 25:760-766.
61. Jackman DM, Miller VA, Cioffredi L-A, Yeap BY, Jänne PA, Riely GJ, Ruiz MG, Giaccone G, Sequist LV, Johnson BE. Impact of epidermal growth factor receptor and KRAS mutations on clinical outcomes in previously untreated non–small cell lung cancer patients: results of an online tumor registry of clinical trials. Clinical Cancer Research. 2009; 15:5267-5273.
62. Jang TW, Oak CH, Chang HK, Suo SJ, Jung MH. EGFR and KRAS mutations in patients with adenocarcinoma of the lung. The Korean journal of internal medicine. 2009; 24:48-54.
63. Jänne PA, Wang X, Socinski MA, Crawford J, Stinchcombe TE, Gu L, Capelletti M, Edelman MJ, Villalona-Calero MA, Kratzke R. Randomized phase II trial of erlotinib alone or with carboplatin and paclitaxel in patients who were never or light former smokers with advanced lung adenocarcinoma: CALGB 30406 trial. Journal of clinical oncology. 2012; 30:2063-2069.
64. Jia X-L, Chen G. EGFR and KRAS mutations in Chinese patients with adenosquamous carcinoma of the lung. Lung cancer (Amsterdam, Netherlands). 2011; 74:396-400.
65. Jiang X, Liu Y, Chen C, Zhan Z, Yan Q, Guo Y, Wang Q, Li K. The value of biomarkers in patients with sarcomatoid carcinoma of the lung: molecular analysis of 33 cases. Clinical lung cancer. 2012; 13:288-296.
66. Jida M, Toyooka S, Mitsudomi T, Takano T, Matsuo K, Hotta K, Tsukuda K, Kubo T, Yamamoto H, Yamane M. Usefulness of cumulative smoking dose for identifying the EGFR mutation and patients with non-small-cell lung cancer for gefitinib treatment. Cancer science. 2009; 100:1931-1934.
67. Jin G, Kim MJ, Jeon H-S, Choi JE, Kim DS, Lee EB, Cha SI, Yoon GS, Kim CH, Jung TH. PTEN mutations and relationship to EGFR, ERBB2, KRAS, and TP53 mutations in non-small cell lung cancers. Lung cancer (Amsterdam, Netherlands). 2010; 69:279-283.
68. Johnson ML, Sima CS, Chaft J, Paik PK, Pao W, Kris MG, Ladanyi M, Riely GJ. Association of KRAS and EGFR mutations with survival in patients with advanced lung adenocarcinomas. Cancer. 2013; 119:356-362.
69. Myerson JS, Iqbal SA, O'Brien ME, Popat S. Supersensitive mutation: two case reports of non-small-cell lung cancer treated with epidermal growth factor receptor tyrosine kinase inhibitors. Clinical lung cancer. 2010; 11:E5-8.
70. Jung M, Kim SH, Lee YJ, Hong S, Kang Y, Kim SK, Chang J, Rha SY, Kim JH, Kim DJ. Prognostic and predictive value of CEA and CYFRA 21-1 levels in advanced non-small cell lung cancer patients treated with gefitinib or erlotinib. Experimental and therapeutic medicine. 2011; 2:685-693.
71. Kaira K, Horie Y, Ayabe E, Murakami H, Takahashi T, Tsuya A, Nakamura Y, Naito T, Endo M, Kondo H, Nakajima T, Yamamoto N. Pulmonary pleomorphic carcinoma: a clinicopathological study including EGFR mutation analysis. Journal of thoracic oncology: official publication of the International Association for the Study of Lung Cancer. 2010; 5:460-465.
72. Watanabe R, Kamiyoshihara M, Kaira K, Motegi A, Takise A. Spontaneous expectoration of primary pulmonary synovial sarcoma. Journal of thoracic oncology: official publication of the International Association for the Study of Lung Cancer. 2006; 1:1025-1026.
73. Kalikaki A, Koutsopoulos A, Trypaki M, Souglakos J, Stathopoulos E, Georgoulias V, Mavroudis D, Voutsina A. Comparison of EGFR and K-RAS gene status between primary tumours and corresponding metastases in NSCLC. British journal of cancer. 2008; 99:923-929.
74. Kalikaki A, Koutsopoulos A, Hatzidaki D, Trypaki M, Kontopodis E, Stathopoulos E, Mavroudis D, Georgoulias V, Voutsina A. Clinical outcome of patients with non-small cell lung cancer receiving front-line chemotherapy according to EGFR and K-RAS mutation status. Lung cancer (Amsterdam, Netherlands). 2010; 69:110-115.
75. Kanaji N, Bandoh S, Ishii T, Kushida Y, Haba R, Kohno K, Dobashi H, Ohnishi H, Matsunaga T. Detection of epidermal growth factor receptor mutations in a few cancer cells from transbronchial cytologic specimens by reverse transcriptase-polymerase chain reaction. Molecular diagnosis & therapy. 2011; 15:353-359.
76. Kang SM, Kang HJ, Shin JH, Kim H, Shin DH, Kim SK, Kim JH, Chung KY, Kim SK, Chang J. Identical epidermal growth factor receptor mutations in adenocarcinomatous and squamous cell carcinomatous components of adenosquamous carcinoma of the lung. Cancer. 2007; 109:581-587.
77. Kanaji N, Bandoh S, Ishii T, Tadokoro A, Watanabe N, Takahama T, Haba R, Imataki O, Dobashi H, Matsunaga T. Detection of EML4-ALK fusion genes in a few cancer cells from transbronchial cytological specimens utilizing immediate cytology during bronchoscopy. Lung cancer (Amsterdam, Netherlands). 2012; 77:293-298.
78. Kato Y, Peled N, Wynes MW, Yoshida K, Pardo M, Mascaux C, Ohira T, Tsuboi M, Matsubayashi J, Nagao T. Novel epidermal growth factor receptor mutation-specific antibodies for non-small cell lung cancer: immunohistochemistry as a possible screening method

for epidermal growth factor receptor mutations. Journal of Thoracic Oncology. 2010; 5:1551-1558.

79. Kato T, Koriyama C, Khan N, Samukawa T, Yanagi M, Hamada T, Yokomakura N, Otsuka T, Inoue H, Sato M. EGFR mutations and human papillomavirus in lung cancer. Lung cancer (Amsterdam, Netherlands). 2012; 78:144-147.

80. Endo K, Konishi A, Sasaki H, Takada M, Tanaka H, Okumura M, Kawahara M, Sugiura H, Kuwabara Y, Fukai I. Epidermal growth factor receptor gene mutation in non-small cell lung cancer using highly sensitive and fast TaqMan PCR assay. Lung cancer (Amsterdam, Netherlands). 2005; 50:375-384.

81. Kawada I, Soejima K, Watanabe H, Nakachi I, Yasuda H, Naoki K, Kawamura M, Eguchi K, Kobayashi K, Ishizaka A. An alternative method for screening EGFR mutation using RFLP in non-small cell lung cancer patients. Journal of Thoracic Oncology. 2008; 3:1096-1103.

82. Kawaguchi T, Ando M, Kubo A, Takada M, Atagi S, Okishio K, Asami K, Matsumura A, Tsujino K, Ignatius OS-H. Long exposure of environmental tobacco smoke associated with activating EGFR mutations in never-smokers with non–small cell lung cancer. Clinical Cancer Research. 2011; 17:39-45.

83. Lozano MD, Zulueta JJ, Echeveste JI, Gurpide A, Seijo LM, Martin-Algarra S, Del Barrio A, Pio R, Idoate MA, Labiano T, Perez-Gracia JL. Assessment of epidermal growth factor receptor and K-ras mutation status in cytological stained smears of non-small cell lung cancer patients: correlation with clinical outcomes. The oncologist. 2011; 16:877-885.

84. Ahn HK, Choi YL, Han JH, Ahn YC, Kim K, Kim J, Shim YM, Um SW, Kim H, Kwon OJ, Sun JM, Ahn JS, Park K, Ahn MJ. Epidermal growth factor receptor mutation and treatment outcome of mediastinoscopic N2 positive non-small cell lung cancer patients treated with neoadjuvant chemoradiotherapy followed by surgery. Lung cancer (Amsterdam, Netherlands). 2013; 79:300-306.

85. Ahn MJ, Yang JC, Liang J, Kang JH, Xiu Q, Chen YM, Blair JM, Peng G, Linn C, Orlando M. Randomized phase II trial of first-line treatment with pemetrexed-cisplatin, followed sequentially by gefitinib or pemetrexed, in East Asian, never-smoker patients with advanced non-small cell lung cancer. Lung cancer (Amsterdam, Netherlands). 2012; 77:346-352.

86. Akamatsu H, Kaira K, Murakami H, Serizawa M, Koh Y, Ono A, Shukuya T, Tsuya A, Nakamura Y, Kenmotsu H, Naito T, Takahashi T, Endo M, Harada H, Nakajima T, Yamamoto N. The Impact of Clinical Outcomes According to EGFR Mutation Status in Patients with Locally Advanced Lung Adenocarcinoma Who Recieved Concurrent Chemoradiotherapy. American journal of clinical oncology. 2012.

87. Akca H, Demiray A, Yaren A, Bir F, Koseler A, Iwakawa R, Bagci G, Yokota J. (2013). Utility of serum DNA and pyrosequencing for the detection of EGFR mutations in non-small cell lung cancer. (United States: Elsevier Inc. (360 Park Avenue South, New York NY 10010, United States)), pp. 73-80.

88. Akerley W, Boucher KM, Bentz JS, Arbogast K, Walters T. (2009). A phase II study of erlotinib as initial treatment for patients with stage IIIB-IV non-small cell lung cancer. (United States: International Association for the Study of Lung Cancer (PO Box 6511, Mail Stop 8111, Aurora, Colorado 80045-0511, United States)), pp. 214-219.

89. Ambrosini-Spaltro A, Campanini N, Bortesi B, Azzoni C, Naldi N, Ampollini L, Tiseo M, Ardizzoni A, Rusca M, Carbognani P, Silini EM. EGFR mutation-specific antibodies in pulmonary adenocarcinoma: a comparison with DNA direct sequencing. Applied immunohistochemistry & molecular morphology: AIMM / official publication of the Society for Applied Immunohistochemistry. 2012; 20:356-362.

90. An SJ, Chen ZH, Su J, Zhang XC, Zhong WZ, Yang JJ, Zhou Q, Yang XN, Huang L, Guan JL, Nie Q, Yan HH, Mok TS, Wu YL. Identification of enriched driver gene alterations in subgroups of non-small cell lung cancer patients based on histology and smoking status. PloS one. 2012; 7:e40109.

91. Andujar P, Pairon JC, Renier A, Descatha A, Hysi I, Abd-Alsamad I, Billon-Galland MA, Blons H, Clin B, Danel C, Debrosse D, Galateau-Salle F, Housset B, Laurent-Puig P, Le Pimpec-Barthes F, Letourneux M, et al. Differential mutation profiles and similar intronic TP53 polymorphisms in asbestos-related lung cancer and pleural mesothelioma. Mutagenesis. 2013; 28:323-331.

92. Angulo B, Conde E, Suarez-Gauthier A, Plaza C, Martinez R, Redondo P, Izquierdo E, Rubio-Viqueira B, Paz-Ares L, Hidalgo M, Lopez-Rios F. A comparison of EGFR mutation testing methods in lung carcinoma: direct sequencing, real-time PCR and immunohistochemistry. PloS one. 2012; 7:e43842.

93. Arrieta O, Cardona AF, Federico Bramuglia G, Gallo A, Campos-Parra AD, Serrano S, Castro M, Aviles A, Amorin E, Kirchuk R, Cuello M, Borbolla J, Riemersma O, Becerra H, Rosell R. (2011). Genotyping non-small cell lung cancer (NSCLC) in latin America. (United States: International Association for the Study of Lung Cancer (PO Box 6511, Mail Stop 8111, Aurora, Colorado 80045-0511, United States)), pp. 1955-1959.

94. Arrieta O, Cardona AF, Gallo A, Bramuglia GF, Campos-Parra AD, Serrano S, Poleri C, Amorin E, Kirchuk R, Cuello M, Arechaga E, Rosell R. Genotyping non-small cell lung cancer (NSCLC) in Latin America (LATAM). American Society of Clinical Oncology).

95. Arrieta O, Cardona AF, Gallo A, Bramuglia GF, Campos-Parra AD, Serrano S, Poleri C, Amorin E, Kirchuk R, Cuello M, Arechaga E, Rosell R. (2011). Genotyping non-small

cell lung cancer (NSCLC) in Latin America (LATAM). American Society of Clinical Oncology).

96. Asahina H, Yamazaki K, Kinoshita I, Sukoh N, Harada M, Yokouchi H, Ishida T, Ogura S, Kojima T, Okamoto Y, Fujita Y, Dosaka-Akita H, Isobe H, Nishimura M. (2006). A phase II trial of gefitinib as first-line therapy for advanced non-small cell lung cancer with epidermal growth factor receptor mutations. (United Kingdom: Nature Publishing Group), pp. 998-1004.
97. Azuma K, Okamoto I, Kawahara A, Taira T, Nakashima K, Hattori S, Kinoshita T, Takeda M, Nakagawa K, Takamori S, Kuwano M, Ono M, Kage M. (2012). Association of the expression of mutant epidermal growth factor receptor protein as determined with mutation-specific antibodies in non-small cell lung cancer with progression-free survival after gefitinib treatment. (United States: International Association for the Study of Lung Cancer (PO Box 6511, Mail Stop 8111, Aurora, Colorado 80045-0511, United States)), pp. 122-127.
98. Bacchi CE, Ciol H, Queiroga EM, Benine LC, Silva LH, Ojopi EB. (2012). Epidermal growth factor receptor and KRAS mutations in Brazilian lung cancer patients. (Brazil: Universidade de Sao Paulo (Av.Dr.Arnaldo 455-Cerqueira Cesar, Sao Paulo 01246903, Brazil)), pp. 419-424.
99. Bae NC, Chae MH, Lee MH, Kim KM, Lee EB, Kim CH, Park TI, Han SB, Jheon S, Jung TH, Park JY. (2007). EGFR, ERBB2, and KRAS mutations in Korean non-small cell lung cancer patients. (United States: Elsevier Inc.), pp. 107-113.
100. Bai H, Han B. (2009). The analysis of erlotinib on brain metastases in patients with non-small-cell lung cancer. (China: Chinese Journal of Lung Cancer (228 Nanjing Rd, Tianjin 300020, China)), pp. 1271-1275.
101. Bai H, Wang Z, Chen K, Zhao J, Lee JJ, Wang S, Zhou Q, Zhuo M, Mao L, An T, Duan J, Yang L, Wu M, Liang Z, Wang Y, Kang X, et al. Influence of chemotherapy on EGFR mutation status among patients with non-small-cell lung cancer. Journal of clinical oncology: official journal of the American Society of Clinical Oncology. 2012; 30:3077-3083.
102. Baykara O, Tansarikaya M, Demirkaya A, Kaynak K, Tanju S, Toker A, Buyru N. Association of epidermal growth factor receptor and K-Ras mutations with smoking history in non-small cell lung cancer patients. Experimental and therapeutic medicine. 2013; 5:495-498.
103. Bell DW, Lynch TJ, Haserlat SM, Harris PL, Okimoto RA, Brannigan BW, Sgroi DC, Muir B, Riemenschneider MJ, Iacona RB, Krebs AD, Johnson DH, Giaccone G, Herbst RS, Manegold C, Fukuoka M, et al. Epidermal growth factor receptor mutations and gene amplification in non-small-cell lung cancer: molecular analysis of the IDEAL/INTACT gefitinib trials. Journal of clinical oncology: official journal of the American Society of Clinical Oncology. 2005; 23:8081-8092.
104. Bichev S, Marinova D, Slavova Y, Kaneva R, Savov A. (2012). Assessment of frequency of the EGFR mutations according to the histological type and sex of bulgarian patients with nsclc. Elsevier Ltd), pp. S275.
105. Billah S, Stewart J, Staerkel G, Chen S, Gong Y, Guo M. EGFR and KRAS mutations in lung carcinoma: molecular testing by using cytology specimens. Cancer cytopathology. 2011; 119:111-117.
106. Bloom KJ, Ha T, Uyeda L, Choppa P. (2012). Evaluation of C-MET fish on non-small cell lung cancer samples with known EGFR mutational status. Nature Publishing Group), pp. 473A.
107. Boch C, Kollmeier J, Roth A, Stephan-Falkenau S, Misch D, Gruning W, Bauer TT, Mairinger T. The frequency of EGFR and KRAS mutations in non-small cell lung cancer (NSCLC): routine screening data for central Europe from a cohort study. BMJ open. 2013; 3.
108. Boldrini L, Gisfredi S, Ursino S, Camacci T, Baldini E, Melfi F, Fontanini G. Mutational analysis in cytological specimens of advanced lung adenocarcinoma: a sensitive method for molecular diagnosis. Journal of thoracic oncology: official publication of the International Association for the Study of Lung Cancer. 2007; 2:1086-1090.
109. Bonanno L, Schiavon M, Nardo G, Bertorelle R, Bonaldi L, Galligioni A, Indraccolo S, Pasello G, Rea F, Favaretto A. Prognostic and predictive implications of EGFR mutations, EGFR copy number and KRAS mutations in advanced stage lung adenocarcinoma. Anticancer research. 2010; 30:5121-5128.
110. Bosdet I, Ali RH, Young S, Karsan A, Ionescu DN. (2012). Cytology samples are comparable to histological samples for egfr mutation testing in non-small cell lung cancer (NSCLC). Nature Publishing Group), pp. 474A.
111. Brachtel EF, Iafrate AJ, Mark EJ, Deshpande V. Cytomorphological correlates of epidermal growth factor receptor mutations in lung carcinoma. Diagnostic cytopathology. 2007; 35:257-262.
112. Brevet M, Johnson ML, Azzoli CG, Ladanyi M. Detection of EGFR mutations in plasma DNA from lung cancer patients by mass spectrometry genotyping is predictive of tumor EGFR status and response to EGFR inhibitors. Lung cancer (Amsterdam, Netherlands). 2011; 73:96-102.
113. Buckingham LE, Coon JS, Morrison LE, Jacobson KK, Jewell SS, Kaiser KA, Mauer AM, Muzzafar T, Polowy C, Basu S, Gale M, Villaflor VM, Bonomi P. The prognostic value of chromosome 7 polysomy in non-small cell lung cancer patients treated with gefitinib. Journal of thoracic oncology: official publication of the International Association for the Study of Lung Cancer. 2007; 2:414-422.

114. Cadranel J, Mauguen A, Faller M, Zalcman G, Buisine MP, Westeel V, Longchampt E, Wislez M, Coudert B, Daniel C, Chetaille B, Michiels S, Blons H, Solassol J, De Fraipont F, Foucher P, et al. Impact of systematic EGFR and KRAS mutation evaluation on progression-free survival and overall survival in patients with advanced non-small-cell lung cancer treated by erlotinib in a French prospective cohort (ERMETIC project--part 2). Journal of thoracic oncology: official publication of the International Association for the Study of Lung Cancer. 2012; 7:1490-1502.

115. Camidge DR, Kono SA, Lu X, Okuyama S, Baron AE, Oton AB, Davies AM, Varella-Garcia M, Franklin W, Doebele RC. Anaplastic lymphoma kinase gene rearrangements in non-small cell lung cancer are associated with prolonged progression-free survival on pemetrexed. Journal of thoracic oncology: official publication of the International Association for the Study of Lung Cancer. 2011; 6:774-780.

116. Cappuzzo F, Hirsch FR, Rossi E, Bartolini S, Ceresoli GL, Bemis L, Haney J, Witta S, Danenberg K, Domenichini I, Ludovini V, Magrini E, Gregorc V, Doglioni C, Sidoni A, Tonato M, et al. (2005). Epidermal growth factor receptor gene and protein and gefitinib sensitivity in non-small-cell lung cancer. (United States, pp. 643-655.

117. Cappuzzo F, Ligorio C, Toschi L, Rossi E, Trisolini R, Paioli D, Magrini E, Finocchiaro G, Bartolini S, Cancellieri A, Hirsch FR, Crino L, Varella-Garcia M. EGFR and HER2 gene copy number and response to first-line chemotherapy in patients with advanced non-small cell lung cancer (NSCLC). Journal of thoracic oncology: official publication of the International Association for the Study of Lung Cancer. 2007; 2:423-429.

118. Cardona AF, Ramos PL, Duarte R, Carranza H, Castro CJ, Lema M, Vargas CA, Jimenez A, Becerra HA, Rosell R. (2011). Screening for mutations in Colombian metastatic non-small cell lung cancer (NSCLC) patients (ONCOLGroup). American Society of Clinical Oncology).

119. Castro AS, Parente B, Goncalves I, Antunes A, Barroso A, Conde S, Neves S, Machado JC. Epidermal Growth Factor Recetor mutation study for 5 years, in a population of patients with non-small cell lung cancer. Revista portuguesa de pneumologia. 2013; 19:7-12.

120. Chamberlin NE, Ward AC. (2011). EGFR mutation prevalence in patients with nonsmall cell lung cancer: An audit of testing within the north of England cancer network. BMJ Publishing Group), pp. A134.

121. Chang YL, Wu CT, Lin SC, Hsiao CF, Jou YS, Lee YC. Clonality and prognostic implications of p53 and epidermal growth factor receptor somatic aberrations in multiple primary lung cancers. Clinical cancer research: an official journal of the American Association for Cancer Research. 2007; 13:52-58.

122. Chang YL, Wu CT, Lin SC, Hsiao CF, Jou YS, Lee YC. (2007). Clonality and prognostic implications of p53 and epidermal growth factor receptor somatic aberrations in multiple primary lung cancers. (United States: American Association for Cancer Research Inc.), pp. 52-58.

123. Chang YL, Wu CT, Shih JY, Lee YC. Comparison of p53 and epidermal growth factor receptor gene status between primary tumors and lymph node metastases in non-small cell lung cancers. (United States: Springer New York (233 Springer Street, New York NY 10013-1578, United States)), pp. 543-550.

124. Chen C-M, Chang J-C, Cheung Y-C, Lin G, Hsieh J-J, Hsu T, Huang S-F. Computed tomography-guided core-needle biopsy specimens demonstrate epidermal growth factor receptor mutations in patients with non-small-cell lung cancer. Acta Radiologica. 2008; 49:991-994.

125. Chen C-H, Gow C-H, Yu C-J, Shih J-Y, Hsu Y-C, Lee W-Y, Yang P-C, Kuo S-H. Clinical response of gefitinib on malignant pleural effusions in patients with non-small cell lung cancer. J Cancer Mol. 2008; 4:23-28.

126. Chen J, Fan M, Jiang G, Fu X, Shen Y, Xie L, Xu X, Zhang G. Concurrent Thoracic Radiotherapy (TRT) With Single-agent Gefitinib In Patients With Pre-treated IIIB/IV Non-small Cell Lung Cancer (NSCLC): Final Report of A Phase I Study With EGFR Mutation Analyses. International Journal of Radiation Oncology* Biology* Physics. 2011; 81:S577-S578.

127. Chen Y-T, Chang JW-C, Liu H-P, Yu T-F, Chiu Y-T, Hsieh J-J, Chen Y-T, Chen Y-R, Wu H-DI, Huang S-F. Clinical implications of high MET gene dosage in non-small cell lung cancer patients without previous tyrosine kinase inhibitor treatment. Journal of Thoracic Oncology. 2011; 6:2027-2035.

128. Chen X, Li W, Hu X, Geng Y, Wang R, Yin Y, Shu Y. Effect of gefitinib challenge to initial treatment with non-small cell lung cancer. Biomedicine & Pharmacotherapy. 2011; 65:542-546.

129. Chen Y-M, Tsai C-M, Fan W-C, Shih J-F, Liu S-H, Wu C-H, Chou T-Y, Lee Y-C, Perng R-P, Whang-Peng J. Phase II randomized trial of erlotinib or vinorelbine in chemonaive, advanced, non-small cell lung cancer patients aged 70 years or older. Journal of Thoracic Oncology. 2012; 7:412-418.

130. Cho BC, Im C-K, Park M-S, Kim SK, Chang J, Park JP, Choi HJ, Kim YJ, Shin S-J, Sohn JH. Phase II study of erlotinib in advanced non–small-cell lung cancer after failure of gefitinib. Journal of clinical oncology. 2007; 25:2528-2533.

131. Choi YH, Lee JK, Kang HJ, Lee TS, Kim H-R, Kim CH, Koh JS, Baek HJ, Lee JC. Association between age at diagnosis and the presence of EGFR mutations in female patients with resected non-small cell lung cancer. Journal of Thoracic Oncology. 2010; 5:1949-1952.

132. Choi Y, Han J, Cho J, Guallar E, Parasuraman B, Lee G, Park K, Lee J, Sun J, Shim YM. 9150 POSTER Testing Practices for EGFR and KRAS in Advanced Non-small Cell

Lung Cancer in a Comprehensive Cancer Care Setting in Korea. European Journal of Cancer. 47:S638-S639.

133. Choi H, Kratz J, Pham P, Lee S, Ray R, Kwon Y-W, Mao J-H, Kang HC, Jablons D, Kim I-J. Development of a rapid and practical mutation screening assay for human lung adenocarcinoma. International journal of oncology. 2012; 40:1900-1906.

134. Choi H, Paeng JC, Kim D-W, Lee JK, Park CM, Kang KW, Chung J-K, Lee DS. Metabolic and metastatic characteristics of ALK-rearranged lung adenocarcinoma on FDG PET/CT. Lung cancer (Amsterdam, Netherlands). 2013; 79:242-247.

135. Choi Y-J, Cho BC, Jeong YH, Seo HJ, Kim HJ, Cho A, Lee JH, Yun M, Jeon TJ, Lee JD. Correlation Between 18F-Fluorodeoxyglucose Uptake and Epidermal Growth Factor Receptor Mutations in Advanced Lung Cancer. Nuclear medicine and molecular imaging. 2012; 46:169-175.

136. Chung CH, Seeley EH, Roder H, Grigorieva J, Tsypin M, Roder J, Burtness BA, Argiris A, Forastiere AA, Gilbert J, Murphy B, Caprioli RM, Carbone DP, Cohen EE. Detection of tumor epidermal growth factor receptor pathway dependence by serum mass spectrometry in cancer patients. Cancer epidemiology, biomarkers & prevention: a publication of the American Association for Cancer Research, cosponsored by the American Society of Preventive Oncology. 2010; 19:358-365.

137. Chung FT, Lee KY, Wang CW, Heh CC, Chan YF, Chen HW, Kuo CH, Feng PH, Lin TY, Wang CH, Chou CL, Chen HC, Lin SM, Kuo HP. Tumor-associated macrophages correlate with response to epidermal growth factor receptor-tyrosine kinase inhibitors in advanced non-small cell lung cancer. International journal of cancer Journal international du cancer. 2012; 131:E227-235.

138. Ck L, Hr L, Yk P, Kt C, Bk L, Ch L, Nl L, J P, P R. (2011). Epidermal growth factor receptor mutations in adenocarcinoma in malaysian patients. Blackwell Publishing), pp. 172-173.

139. Conde E, Angulo B, Tang M, Morente M, Torres-Lanzas J, Lopez-Encuentra A, Lopez-Rios F, Sanchez-Cespedes M. Molecular context of the EGFR mutations: evidence for the activation of mTOR/S6K signaling. Clinical cancer research. 2006; 12:710-717.

140. Cortes-Funes H, Gomez C, Rosell R, Valero P, Garcia-Giron C, Velasco A, Izquierdo A, Diz P, Camps C, Castellanos D. Epidermal growth factor receptor activating mutations in Spanish gefitinib-treated non-small-cell lung cancer patients. Annals of oncology. 2005; 16:1081-1086.

141. Cote ML, Haddad R, Edwards DJ, Atikukke G, Gadgeel S, Soubani AO, Lonardo F, Bepler G, Schwartz AG, Ethier SP. Frequency and type of epidermal growth factor receptor mutations in African Americans with non-small cell lung cancer. Journal of Thoracic Oncology. 2011; 6:627-630.

142. Dacic S, Shuai Y, Yousem S, Ohori P, Nikiforova M. Clinicopathological predictors of EGFR/KRAS mutational status in primary lung adenocarcinomas. Modern Pathology. 2010; 23:159-168.

143. D'Angelo SP, Pietanza MC, Johnson ML, Riely GJ, Miller VA, Sima CS, Zakowski MF, Rusch VW, Ladanyi M, Kris MG. Incidence of EGFR exon 19 deletions and L858R in tumor specimens from men and cigarette smokers with lung adenocarcinomas. Journal of Clinical Oncology. 2011; 29:2066-2070.

144. Daniele L, Cassoni P, Bacillo E, Cappia S, Righi L, Volante M, Tondat F, Inghirami G, Sapino A, Scagliotti GV. Epidermal growth factor receptor gene in primary tumor and metastatic sites from non-small cell lung cancer. Journal of Thoracic Oncology. 2009; 4:684-688.

145. Dasgupta S, Soudry E, Mukhopadhyay N, Shao C, Yee J, Lam S, Lam W, Zhang W, Gazdar AF, Fisher PB. Mitochondrial DNA mutations in respiratory complex-I in never-smoker lung cancer patients contribute to lung cancer progression and associated with EGFR gene mutation. Journal of cellular physiology. 2012; 227:2451-2460.

146. Eberhard DA, Johnson BE, Amler LC, Goddard AD, Heldens SL, Herbst RS, Ince WL, Jänne PA, Januario T, Johnson DH. Mutations in the epidermal growth factor receptor and in KRAS are predictive and prognostic indicators in patients with non–small-cell lung cancer treated with chemotherapy alone and in combination with erlotinib. Journal of Clinical Oncology. 2005; 23:5900-5909.

147. de Mello RA, Pires FS, Marques DS, Oliveira J, Rodrigues A, Soares M, Azevedo I, Peixoto A, Santos C, Pinto C. EGFR exon mutation distribution and outcome in non-small-cell lung cancer: a Portuguese retrospective study. Tumor Biology. 2012; 33:2061-2068.

148. De Pas T, Toffalorio F, Manzotti M, Fumagalli C, Spitaleri G, Catania C, Delmonte A, Giovannini M, Spaggiari L, de Braud F. Activity of epidermal growth factor receptor-tyrosine kinase inhibitors in patients with non-small cell lung cancer harboring rare epidermal growth factor receptor mutations. Journal of Thoracic Oncology. 2011; 6:1895-1901.

149. DENG QF, ZHOU CC, SU CX. Clinicopathological features and epidermal growth factor receptor mutations associated with epithelial–mesenchymal transition in non-small cell lung cancer. Respirology (Carlton, Vic). 2009; 14:371-376.

150. Dingemans A-M, De Langen A, Van Den Boogaart V, Marcus J, Backes W, Scholtens H, van Tinteren H, Hoekstra O, Pruim J, Brans B. First-line erlotinib and bevacizumab in patients with locally advanced and/or metastatic non-small-cell lung cancer: a phase II study including molecular imaging. Annals of Oncology. 2010:mdq391.

151. Doebele RC, Lu X, Sumey C, Maxson DA, Weickhardt AJ, Oton AB, Bunn PA, Barón AE, Franklin WA, Aisner DL. Oncogene status predicts patterns of metastatic spread in treatment-naive nonsmall cell lung cancer. Cancer. 2012; 118:4502-4511.
152. Dogan S, Shen R, Ang DC, Johnson ML, D'Angelo SP, Paik PK, Brzostowski EB, Riely GJ, Kris MG, Zakowski MF. Molecular epidemiology of EGFR and KRAS mutations in 3,026 lung adenocarcinomas: higher susceptibility of women to smoking-related KRAS-mutant cancers. Clinical Cancer Research. 2012; 18:6169-6177.
153. Dong Q, Han B, Huang J, Yang L, Huang J, Zhao C, Lu L. [Analysis of EGFR mutations in 176 cases of non-small cell lung cancer]. Chinese journal of oncology. 2006; 28:686-690.
154. Dongiovanni D, Daniele L, Barone C, Dongiovanni V, Fissore C, Sapino A, Macrì L, Bussolati G, Buffoni L, Gaspari F. Gefitinib (ZD1839): therapy in selected patients with non-small cell lung cancer (NSCLC)? Lung cancer (Amsterdam, Netherlands). 2008; 61:73-81.
155. Eberhardt W, Thomas M, Graf Von Der Schulenburg JM, Dietel M, Schirmacher P, Gutendorf B, Zirrgiebel U, Schutte W. (2011). EGFR mutation testing and first line treatment of patients with advanced NSCLC and positive EGFR mutation status - Results from a German registry. Elsevier Ltd), pp. S636.
156. Ebi N, Semba H, ji Tokunaga S, Takayama K, Wataya H, Kuraki T, Yamamoto H, ji Akamine S, Okamoto I, Nakanishi Y. A phase II trial of gefitinib monotherapy in chemotherapy-naive patients of 75 years or older with advanced non-small cell lung cancer. Journal of Thoracic Oncology. 2008; 3:1166-1171.
157. Eichler AF, Kahle KT, Wang DL, Joshi VA, Willers H, Engelman JA, Lynch TJ, Sequist LV. EGFR mutation status and survival after diagnosis of brain metastasis in nonsmall cell lung cancer. Neuro-oncology. 2010:noq076.
158. El-Zammar OA, Zhang S, Katzenstein A-LA. Comparison of FISH, PCR, and immunohistochemistry in assessing EGFR status in lung adenocarcinoma and correlation with clinicopathologic features. Diagnostic Molecular Pathology. 2009; 18:133-137.
159. Kawahara A, Azuma K, Hattori S, Nakashima K, Basaki Y, Akiba J, Takamori S, Aizawa H, Yanagawa T, Izumi H. The close correlation between 8-hydroxy-2′-deoxyguanosine and epidermal growth factor receptor activating mutation in non–small cell lung cancer. Human pathology. 2010; 41:951-959
160. Kawano D, Yano T, Shoji F, Ito K, Morodomi Y, Haro A, Miura N, Takenaka T, Yoshino I, Maehara Y. The influence of intracellular epidermal growth factor receptor (EGFR) signal activation on the outcome of EGFR tyrosine kinase inhibitor treatment for pulmonary adenocarcinoma. Surgery today. 2011; 41:818-823.
161. Kayatani H, Fujiwara K, Tokimasa Y, Higo H, Kameyama N, Matsushita M, Matsuo K, Yonei T, Sato T. (2011). Erlotinib for advanced non-small cell lung cancer: Evaluation in single institute. Blackwell Publishing), pp. 164.
162. Kim K-S, Jeong J-Y, Kim Y-C, Na K-J, Kim Y-H, Ahn S-J, Baek S-M, Park C-S, Park C-M, Kim Y-I. Predictors of the Response to Gefitinib in Refractory Non–Small Cell Lung Cancer. Clinical Cancer Research. 2005; 11:2244-2251.
163. Kim YT, Kim T-y, Lee DS, Park SJ, Park J-y, Seo S-J, Choi H-S, Kang HJ, Hahn S, Kang CH. Molecular changes of epidermal growth factor receptor (EGFR) and KRAS and their impact on the clinical outcomes in surgically resected adenocarcinoma of the lung. Lung cancer (Amsterdam, Netherlands). 2008; 59:111-118.
164. Kim D, Min H, Lee K, Kim Y, Oh D, Jeon Y, Lee S, Im S, Chung D, Kim Y. High tumour islet macrophage infiltration correlates with improved patient survival but not with EGFR mutations, gene copy number or protein expression in resected non-small cell lung cancer. British journal of cancer. 2008; 98:1118-1124.
165. Kim ST, Choi YJ, Park KH, Oh SC, Seo JH, Shin SW, Kim JS, Kim YH. Epidermal growth factor receptor mutations as a prognostic factor in Korean patients with advanced lung adenocarcinoma who had not been treated with received epidermal growth factor receptor tyrosine kinase inhibitors. Chemotherapy. 2011; 57:108-114.
166. Kim HJ, Kim WS, Shin K-C, Lee GH, Kim M-J, Lee JE, Song KS, Kim SY, Lee KY. Comparative analysis of peptide nucleic acid (PNA)-mediated real-time PCR clamping and DNA direct sequencing for EGFR mutation detection. Tuberculosis and respiratory diseases. 2011; 70:21-27.
167. Kim HJ, Lee KY, Kim Y-C, Kim K-S, Lee SY, Jang TW, Lee MK, Shin K-C, Lee GH, Lee JC. Detection and comparison of peptide nucleic acid-mediated real-time polymerase chain reaction clamping and direct gene sequencing for epidermal growth factor receptor mutations in patients with non-small cell lung cancer. Lung cancer (Amsterdam, Netherlands). 2012; 75:321-325.
168. Moon J-H, Heo JS, Kim JS, Jun EK, Lee JH, Kim A, Kim J, Whang KY, Kang Y-K, Yeo S. Reprogramming fibroblasts into induced pluripotent stem cells with Bmi1. Cell research. 2011; 21:1305-1315.
169. Kim HJ, Oh SY, Kim WS, Kim SJ, Yoo GH, Kim WD, Lee KY. Clinical investigation of EGFR mutation detection by pyrosequencing in lung cancer patients. Oncology letters. 2013; 5:271-276.
170. Kim YH, Hirabayashi M, Togashi Y, Hirano K, Tomii K, Masago K, Kaneda T, Yoshimatsu H, Otsuka K, Mio T. Phase II study of carboplatin and pemetrexed in advanced non-squamous, non-small-cell lung cancer: Kyoto Thoracic Oncology Research Group Trial 0902. Cancer chemotherapy and pharmacology. 2012; 70:271-276.

171. Kim HR, Shim HS, Chung JH, Lee YJ, Hong YK, Rha SY, Kim SH, Ha SJ, Kim SK, Chung KY. Distinct clinical features and outcomes in never-smokers with nonsmall cell lung cancer who harbor EGFR or KRAS mutations or ALK rearrangement. Cancer. 2012; 118:729-739.
172. Kim YT, Seong YW, Jung YJ, Jeon YK, Park IK, Kang CH, Kim JH. The Presence of Mutations in Epidermal Growth Factor Receptor Gene Is Not a Prognostic Factor for Long-Term Outcome after Surgical Resection of Non–Small-Cell Lung Cancer. Journal of Thoracic Oncology. 2013; 8:171-178.
173. Kim ST, Sung JS, Jo UH, Park KH, Shin SW, Kim YH. Can mutations of EGFR and KRAS in serum be predictive and prognostic markers in patients with advanced non-small cell lung cancer (NSCLC)? Medical Oncology. 2013; 30:1-10.
174. Kimura H, Kasahara K, Kawaishi M, Kunitoh H, Tamura T, Holloway B, Nishio K. Detection of epidermal growth factor receptor mutations in serum as a predictor of the response to gefitinib in patients with non–small-cell lung cancer. Clinical Cancer Research. 2006; 12:3915-3921.
175. Kimura H, Fujiwara Y, Sone T, Kunitoh H, Tamura T, Kasahara K, Nishio K. High sensitivity detection of epidermal growth factor receptor mutations in the pleural effusion of non-small cell lung cancer patients. Cancer science. 2006; 97:642-648.
176. Koga T, Takeshita M, Yano T, Maehara Y, Sueishi K. CHFR hypermethylation and EGFR mutation are mutually exclusive and exhibit contrastive clinical backgrounds and outcomes in non-small cell lung cancer. International Journal of Cancer. 2011; 128:1009-1017.
177. Kosaka T, Yatabe Y, Endoh H, Kuwano H, Takahashi T, Mitsudomi T. Mutations of the epidermal growth factor receptor gene in lung cancer biological and clinical implications. Cancer research. 2004; 64:8919-8923.
178. Kosaka T, Yatabe Y, Onozato R, Kuwano H, Mitsudomi T. Prognostic implication of EGFR, KRAS, and TP53 gene mutations in a large cohort of Japanese patients with surgically treated lung adenocarcinoma. Journal of Thoracic Oncology. 2009; 4:22-29.
179. Koudelakova V, Trojanec R, Kneblova M, Drabek J, Kolar Z, Kolek V, Mlcochova S, Hajduch M. 582 ALK Rearrangement and EGFR Copy Number Gains/mutations in Czech Non-small Cell Lung Cancer Patients. european journal of cancer. 2012; 48:S139.
180. Koyama N, Jinn Y, Takabe K, Yoshizawa M, Usui Y, Inase N, Miyake S, Yoshizawa Y, Hagiwara K, Kanazawa M. The characterization of gefitinib sensitivity and adverse events in patients with non-small cell lung cancer. Anticancer research. 2006; 26:4519-4525.
181. Lai R, Xie L, Shen L, Zhu C, Qian J. [Epithelial growth factor receptor (EGFR) exon double-sequencing analysis in NSClC]. Chinese journal of oncology. 2006; 28:599-602.
182. Leary AF, de Castro DG, Nicholson AG, Ashley S, Wotherspoon A, O'Brien ME, Popat S. Establishing an EGFR mutation screening service for non-small cell lung cancer–Sample quality criteria and candidate histological predictors. European Journal of Cancer. 2012; 48:61-67.
183. Sequist LV, Gettinger S, Senzer NN, Martins RG, Jänne PA, Lilenbaum R, Gray JE, Iafrate AJ, Katayama R, Hafeez N. Activity of IPI-504, a novel heat-shock protein 90 inhibitor, in patients with molecularly defined non–small-cell lung cancer. Journal of Clinical Oncology. 2010:JCO. 2010.2030. 8338.
184. Lee K-H, Han S-W, Hwang PG, Oh D-Y, Kim D-W, Chung DH, Im S-A, Kim T-Y, Heo DS, Bang Y-J. Epidermal growth factor receptor mutations and response to chemotherapy in patients with non-small-cell lung cancer. Japanese journal of clinical oncology. 2006; 36:344-350.
185. Lee K-H, Min HS, Han S-W, Oh D-Y, Lee S-H, Kim D-W, Im S-A, Chung DH, Kim YT, Kim T-Y. ERCC1 expression by immunohistochemistry and EGFR mutations in resected non-small cell lung cancer. Lung cancer (Amsterdam, Netherlands). 2008; 60:401-407.
186. Lee YJ, Park IK, Park M-S, Choi HJ, Cho BC, Chung KY, Kim SK, Chang J, Moon JW, Kim H. Activating mutations within the EGFR kinase domain: a molecular predictor of disease-free survival in resected pulmonary adenocarcinoma. Journal of cancer research and clinical oncology. 2009; 135:1647-1654.
187. Lee YJ, Shim HS, Kang YA, Hong SJ, Kim HK, Kim H, Kim SK, Choi SH, Kim J-H, Cho BC. Dose effect of cigarette smoking on frequency and spectrum of epidermal growth factor receptor gene mutations in Korean patients with non-small cell lung cancer. Journal of cancer research and clinical oncology. 2010; 136:1937-1944.
188. Lee YJ, Cho BC, Jee SH, Moon JW, Kim SK, Chang J, Chung KY, Park IK, Choi SH, Kim J-H. Impact of environmental tobacco smoke on the incidence of mutations in epidermal growth factor receptor gene in never-smoker patients with non–small-cell lung cancer. Journal of Clinical Oncology. 2010; 28:487-492.
189. Lee SY, Kim MJ, Jin G, Yoo SS, Park JY, Choi JE, Jeon HS, Cho S, Lee EB, Cha SI. Somatic mutations in epidermal growth factor receptor signaling pathway genes in non-small cell lung cancers. Journal of Thoracic Oncology. 2010; 5:1734-1740.
190. Lee KY, Kim HJ, Kim SJ, Yoo GH, Kim WD, Oh SY, Kim WS. PNA-mediated PCR clamping for the detection of EGFR mutations in non-small cell lung cancer. Tuberc Respir Dis. 2010; 69:271-278.
191. Lee H-J, Kim YT, Kang CH, Zhao B, Tan Y, Schwartz LH, Persigehl T, Jeon YK, Chung DH. Epidermal growth factor receptor mutation in lung adenocarcinomas: relationship with CT characteristics and histologic subtypes. Radiology. 2013; 268:254-264.

192. Lee J-O, Kim TM, Lee S-H, Kim D-W, Kim S, Jeon Y-K, Chung DH, Kim W-H, Kim YT, Yang S-C. Anaplastic lymphoma kinase translocation: a predictive biomarker of pemetrexed in patients with non-small cell lung cancer. Journal of thoracic oncology. 2011; 6:1474-1480.

193. Lee H-L, Chung T-S, Ting L-L, Tsai J-T, Chen S-W, Chiou J-F, Leung HW-C, Liu HE. EGFR mutations are associated with favorable intracranial response and progression-free survival following brain irradiation in non-small cell lung cancer patients with brain metastases. Radiation Oncology. 2012; 7:1.

194. Lee HJ, Xu X, Kim H, Jin Y, Sun P, Kim JE, Chung J-H. Comparison of Direct Sequencing, PNA Clamping-Real Time Polymerase Chain Reaction, and Pyrosequencing Methods for the Detection of EGFR Mutations in Non-small Cell Lung Carcinoma and the Correlation with Clinical Responses to EGFR Tyrosin. The Korean Journal of Pathology. 2013; 47:52-60.

195. Leidner RS, Fu P, Clifford B, Hamdan A, Jin C, Eisenberg R, Boggon TJ, Skokan M, Franklin WA, Cappuzzo F. Genetic abnormalities of the EGFR pathway in African American patients with non–small-cell lung cancer. Journal of Clinical Oncology. 2009; 27:5620-5626.

196. Fu P, Panneerselvam A, Clifford B, Dowlati A, Ma P, Zeng G, Halmos B, Leidner R. Simpson's paradox - aggregating and partitioning populations in health disparities of lung cancer patients. Statistical methods in medical research. 2012.

197. Zhang Z, Kobayashi S, Borczuk AC, Leidner RS, Laframboise T, Levine AD, Halmos B. Dual specificity phosphatase 6 (DUSP6) is an ETS-regulated negative feedback mediator of oncogenic ERK signaling in lung cancer cells. Carcinogenesis. 2010; 31:577-586.

198. Li R, Chen Y, Leng Y. Clinical significance of EGFR mutation in NSCLC tissues. Chin J Cancer Prev Treat. 2008; 15:1557-1560.

199. Li AR, Chitale D, Riely GJ, Pao W, Miller VA, Zakowski MF, Rusch V, Kris MG, Ladanyi M. EGFR mutations in lung adenocarcinomas: clinical testing experience and relationship to EGFR gene copy number and immunohistochemical expression. The Journal of Molecular Diagnostics. 2008; 10:242-248.

200. Li Z, Zhang L, Wang W, Guo K, Shao J, Rong T. [Correlation between EGFR gene mutation and high copy number and their association with the clinicopathological features in Chinese patients with non-small cell lung cancer]. Chinese journal of oncology. 2011; 33:666-670.

201. Li C, Sun Y, Fang Z, Han X, Fang R, Zhang Y, Pan Y, Zhang W, Ren Y, Ji H. Comprehensive analysis of epidermal growth factor receptor gene status in lung adenocarcinoma. Journal of Thoracic Oncology. 2011; 6:1016-1021.

202. Li M, Zhang Q, Liu L, Liu Z, Zhou L, Wang Z, Yue S, Xiong H, Feng L, Lu S. The different clinical significance of EGFR mutations in exon 19 and 21 in non-small cell lung cancer patients of China. Neoplasma. 2010; 58:74-81.

203. Fuhai L, Hua B, Xia'nan L, Meina W, Rong Y, Anhui S, Li Y, Jie W, Guangying Z. Role of EGFR mutation status in patients with stage III non-squamous non-small cell lung cancer treated with chemoradiotherapy. Chinese Journal of Lung Cancer. 2011; 14:715-718.

204. Li B, Wang Y, Zhu H, Li J, Hu X, Wang B, Hao X, Wang L, Zhang X, Shi Y. [Association of serum EGFR protein concentration with the efficacy of Gefitinib in the treatment of advanced non-small cell lung cancer]. Chinese journal of oncology. 2011; 33:431-435.

205. Li H, Pan Y, Li Y, Li C, Wang R, Hu H, Zhang Y, Ye T, Wang L, Shen L. Frequency of well-identified oncogenic driver mutations in lung adenocarcinoma of smokers varies with histological subtypes and graduated smoking dose. Lung cancer (Amsterdam, Netherlands). 2013; 79:8-13.

206. Liang Z, Zeng X, Zhang J, Wu S, Gao J, Liu T. [Status of gene mutation and copy number of EGFR in 290 cases of non-small cell lung carcinoma]. Chinese journal of pathology. 2008; 37:654-659.

207. Liang Z, Zhang J, Zeng X, Gao J, Wu S, Liu T. Relationship between EGFR expression, copy number and mutation in lung adenocarcinomas. BMC cancer. 2010; 10:1.

208. Lim EH, Zhang S-L, Li J-L, Yap W-S, Howe T-C, Tan B-P, Lee Y-S, Wong D, Khoo K-L, Seto K-Y. Using whole genome amplification (WGA) of low-volume biopsies to assess the prognostic role of EGFR, KRAS, p53, and CMET mutations in advanced-stage non-small cell lung cancer (NSCLC). Journal of Thoracic Oncology. 2009; 4:12-21.

209. Lin C-C, Hsu H-H, Sun C-T, Shih J-Y, Lin Z-Z, Yu C-J, Chen GG, Hsin MKY, Lam KC, Leung L. Chemotherapy response in East Asian non-small cell lung cancer patients harboring wild-type or activating mutation of epidermal growth factor receptors. Journal of Thoracic Oncology. 2010; 5:1424-1429.

210. Lind JS, Dingemans A-MC, Groen HJ, Thunnissen FB, Bekers O, Heideman DA, Honeywell RJ, Giovannetti E, Peters GJ, Postmus PE. A multicenter phase II study of erlotinib and sorafenib in chemotherapy-naive patients with advanced non–small cell lung cancer. Clinical Cancer Research. 2010; 16:3078-3087.

211. Liu F, Jiang B, Gong S, Yao B, Zhang W, Zhu G, Zhu Z, Gong Y, Wang M, Hu X. [Mutational analysis of EGFR and K-RAS in Chinese patients with non-small cell lung cancer]. Chinese journal of medical genetics. 2007; 24:31-34.

212. Liu Y, Xu ML, Zhong HH, Heng WJ, Wu BQ. EGFR mutations are more frequent in well-differentiated than in poor-differentiated lung adenocarcinomas. Pathology & Oncology Research. 2008; 14:373-379.

213. Liu H, Li Y, Chen G, Wang J, Wang Y, Wei S, Zhu D, Qiu X, Wang W, Song Z. [Detection and Its Clinical Significance

of EGFR Gene Mutation and Gene Amplification in 187 Patients with Non-small Cell Lung Cancer.]. Chinese journal of lung cancer. 2009; 12:1219-1228.

214. Liu H-P, Wu H-DI, Chang JW-C, Wu Y-C, Yang H-Y, Chen Y-T, Hsieh W-Y, Chen Y-T, Chen Y-R, Huang S-F. Prognostic implications of epidermal growth factor receptor and KRAS gene mutations and epidermal growth factor receptor gene copy numbers in patients with surgically resectable non-small cell lung cancer in Taiwan. Journal of Thoracic Oncology. 2010; 5:1175-1184.
215. Liu Y, Liu B, Li X-Y, Li J-J, Qin H-F, Tang C-H, Guo W-F, Hu H-X, Li S, Chen C-J. A comparison of ARMS and direct sequencing for EGFR mutation analysis and tyrosine kinase inhibitors treatment prediction in body fluid samples of non-small-cell lung cancer patients. Journal of Experimental & Clinical Cancer Research. 2011; 30:1.
216. Liu Y, Zhu J, Zhang L, Zhang T, Zhang Z, Wang C. [Epithelial growth factor receptor mutation status to the effective of survival in non-small cell lung cancer after surgery]. Chinese journal of surgery. 2012; 50:1082-1086.
217. Liu B, Shi S, Wang X, Xu Y, Zhang X, Yu B, Lu Z, Wang J, Zhou X. [Relevance of molecular alterations in histopathologic subtyping of lung adenocarcinoma based on 2011 International Multidisciplinary Lung Adenocarcinoma Classification]. Chinese journal of pathology. 2012; 41:505-510.
218. Loprevite M, Tiseo M, Chiaramondia M, Capelletti M, Bozzetti C, Bortesi B, Naldi N, Nizzoli R, Dadati P, Kunkl A. Buccal Mucosa Cells as *In vivo* Model to Evaluate Gefitinib Activity in Patients with Advanced Non–Small Cell Lung Cancer. Clinical Cancer Research. 2007; 13:6518-6526.
219. Lozano MD, Zulueta JJ, Echeveste JI, Gúrpide A, Seijo LM, Martín-Algarra S, Del Barrio A, Pio R, Idoate MA, Labiano T. Assessment of epidermal growth factor receptor and K-ras mutation status in cytological stained smears of non-small cell lung cancer patients: correlation with clinical outcomes. The oncologist. 2011; 16:877-885.
220. Lu H-Y, Su D, Pan X-D, Jiang H, Ma S-L. Mutation and expression of multiple treatment response-related genes in a population with locally advanced non-small cell lung cancer. Oncology letters. 2012; 3:415-420.
221. Ludovini V, Bianconi F, Pistola L, Chiari R, Minotti V, Colella R, Giuffrida D, Tofanetti FR, Siggillino A, Flacco A. Phosphoinositide-3-kinase catalytic alpha and KRAS mutations are important predictors of resistance to therapy with epidermal growth factor receptor tyrosine kinase inhibitors in patients with advanced non-small cell lung cancer. Journal of Thoracic Oncology. 2011; 6:707-715.
222. Lv C, Ma Y, Feng Q, Fang F, Bai H, Zhao B, Yan S, Wu N, Zheng Q, Li S. A pilot study: sequential gemcitabine/cisplatin and icotinib as induction therapy for stage IIB to IIIA non-small-cell lung adenocarcinoma. World journal of surgical oncology. 2013; 11:1.
223. Mack PC, Holland WS, Burich RA, Sangha R, Solis LJ, Li Y, Beckett LA, Lara PN, Davies AM, Gandara DR. EGFR mutations detected in plasma are associated with patient outcomes in erlotinib plus docetaxel-treated non-small cell lung cancer. Journal of Thoracic Oncology. 2009; 4:1466-1472.
224. Mak RH, Doran E, Muzikansky A, Kang J, Neal JW, Baldini EH, Choi NC, Willers H, Jackman DM, Sequist LV. Outcomes after combined modality therapy for EGFR-mutant and wild-type locally advanced NSCLC. The oncologist. 2011; 16:886-895.
225. Malapelle U, de Rosa N, Rocco D, Bellevicine C, Crispino C, Illiano A, Piantedosi FV, Nappi O, Troncone G. EGFR and KRAS mutations detection on lung cancer liquid-based cytology: a pilot study. Journal of clinical pathology. 2012; 65:87-91.
226. Marchetti A, Martella C, Felicioni L, Barassi F, Salvatore S, Chella A, Camplese PP, Iarussi T, Mucilli F, Mezzetti A. EGFR mutations in non–small-cell lung cancer: analysis of a large series of cases and development of a rapid and sensitive method for diagnostic screening with potential implications on pharmacologic treatment. Journal of Clinical Oncology. 2005; 23:857-865.
227. Marks JL, Broderick S, Zhou Q, Chitale D, Li AR, Zakowski MF, Kris MG, Rusch VW, Azzoli CG, Seshan VE. Prognostic and therapeutic implications of EGFR and KRAS mutations in resected lung adenocarcinoma. Journal of thoracic oncology. 2008; 3:111-116.
228. Martinez P, Hernández-Losa J, Montero MÁ, Cedrés S, Castellví J, Martinez-Marti A, Tallada N, Murtra-Garrell N, Navarro-Mendivill A, Rodriguez-Freixinos V. Fluorescence in situ hybridization and immunohistochemistry as diagnostic methods for ALK positive non-small cell lung cancer patients. PloS one. 2013; 8:e52261.
229. Martínez-Navarro EM, Rebollo J, González-Manzano R, Sureda M, Evgenyeva E, Valenzuela B, Fernández FJ, Forteza J, Brugarolas A. Epidermal growth factor receptor (EGFR) mutations in a series of non-small-cell lung cancer (NSCLC) patients and response rate to EGFR-specific tyrosine kinase inhibitors (TKIs). Clinical and Translational Oncology. 2011; 13:812-818.
230. Masago K, Fujita S, Hatachi Y, Fukuhara A, Sakuma K, Ichikawa M, Kim YH, Mio T, Mishima M. Clinical significance of pretreatment serum amphiregulin and transforming growth factor-α, and an epidermal growth factor receptor somatic mutation in patients with advanced non-squamous, non-small cell lung cancer. Cancer science. 2008; 99:2295-2301.
231. Masago K, Fujita S, Togashi Y, Kim YH, Hatachi Y, Fukuhara A, Nagai H, Sakamori Y, Mio T, Mishima M. Clinicopathologic Factors Affecting the Progression-Free Survival of Patients With Advanced Non–Small-Cell Lung Cancer After Gefitinib Therapy. Clinical lung cancer. 2011; 12:56-61.

232. Massarelli E, Varella-Garcia M, Tang X, Xavier AC, Ozburn NC, Liu DD, Bekele BN, Herbst RS, Wistuba II. KRAS mutation is an important predictor of resistance to therapy with epidermal growth factor receptor tyrosine kinase inhibitors in non–small-cell lung cancer. Clinical Cancer Research. 2007; 13:2890-2896.

233. Matsumoto S, Takahashi K, Iwakawa R, Matsuno Y, Nakanishi Y, Kohno T, Shimizu E, Yokota J. Frequent EGFR mutations in brain metastases of lung adenocarcinoma. International journal of cancer. 2006; 119:1491-1494.

234. McMillen E, Ye F, Li G, Wu Y, Yin G, Liu W. Epidermal growth factor receptor (EGFR) mutation and p-EGFR expression in resected non-small cell lung cancer. Experimental lung research. 2010; 36:531-537.

235. Milella M, Nuzzo C, Bria E, Sperduti I, Visca P, Buttitta F, Antoniani B, Merola R, Gelibter A, Cuppone F. EGFR molecular profiling in advanced NSCLC: a prospective phase II study in molecularly/clinically selected patients pretreated with chemotherapy. Journal of Thoracic Oncology. 2012; 7:672-680.

236. Miller VA, Riely GJ, Zakowski MF, Li AR, Patel JD, Heelan RT, Kris MG, Sandler AB, Carbone DP, Tsao A. Molecular characteristics of bronchioloalveolar carcinoma and adenocarcinoma, bronchioloalveolar carcinoma subtype, predict response to erlotinib. Journal of Clinical Oncology. 2008; 26:1472-1478.

237. Mitsudomi T, Kosaka T, Endoh H, Horio Y, Hida T, Mori S, Hatooka S, Shinoda M, Takahashi T, Yatabe Y. (2005). Mutations of the epidermal growth factor receptor gene predict prolonged survival after gefitinib treatment in patients with non-small-cell lung cancer with postoperative recurrence. (United States, pp. 2513-2520.

238. Miyamae Y, Shimizu K, Hirato J, Araki T, Tanaka K, Ogawa H, Kakegawa S, Sugano M, Nakano T, Mitani Y. Significance of epidermal growth factor receptor gene mutations in squamous cell lung carcinoma. Oncology reports. 2011; 25:921.

239. Mok TS, Wu Y-L, Yu C-J, Zhou C, Chen Y-M, Zhang L, Ignacio J, Liao M, Srimuninnimit V, Boyer MJ. Randomized, placebo-controlled, phase II study of sequential erlotinib and chemotherapy as first-line treatment for advanced non–small-cell lung cancer. Journal of Clinical Oncology. 2009; 27:5080-5087.

240. Molina-Vila MA, Bertran-Alamillo J, Reguart N, Taron M, Castella E, Llatjós M, Costa C, Mayo C, Pradas A, Queralt C. A sensitive method for detecting EGFR mutations in non-small cell lung cancer samples with few tumor cells. Journal of Thoracic Oncology. 2008; 3:1224-1235.

241. Morinaga R, Okamoto I, Fujita Y, Arao T, Sekijima M, Nishio K, Ito H, Fukuoka M, Kadota Ji, Nakagawa K. Association of epidermal growth factor receptor (EGFR) gene mutations with EGFR amplification in advanced non-small cell lung cancer. Cancer science. 2008; 99:2455-2460.

242. Mu XL, Li LY, Zhang XT, Wang MZ, Feng RE, Cui QC, Zhou HS, Guo BQ. Gefitinib-sensitive mutations of the epidermal growth factor receptor tyrosine kinase domain in Chinese patients with non–small cell lung cancer. Clinical cancer research. 2005; 11:4289-4294.

243. 88. Na II, Rho JK, Choi YJ, Kim CH, Koh JS, Ryoo B-Y, Yang SH, Lee JC. Clinical features reflect exon sites of EGFR mutations in patients with resected non-small-cell lung cancer. Journal of Korean medical science. 2007; 22:393-399.

244. Kim H-R, Lee JK, Park SH, Kim CH, Koh JS, Baek HJ, Choe DH. Epidermal growth factor receptor mutations in female patients with postoperative recurrent non-small-cell lung cancer. Journal of cancer research and therapeutics. 2012; 8:373.

245. Nagashima O, Ohashi R, Yoshioka Y, Inagaki A, Tajima M, Koinuma Y, Iwakami S-i, Iwase A, Sasaki S, Tominaga S. High prevalence of gene abnormalities in young patients with lung cancer. Journal of thoracic disease. 2013; 5:27-30.

246. Nakajima T, Yasufuku K, Suzuki M, Hiroshima K, Kubo R, Mohammed S, Miyagi Y, Matsukuma S, Sekine Y, Fujisawa T. Assessment of epidermal growth factor receptor mutation by endobronchial ultrasound-guided transbronchial needle aspiration. CHEST Journal. 2007; 132:597-602.

247. Nakajima T, Yasufuku K, Nakagawara A, Kimura H, Yoshino I. Multigene mutation analysis of metastatic lymph nodes in non-small cell lung cancer diagnosed by endobronchial ultrasound-guided transbronchial needle aspiration. CHEST Journal. 2011; 140:1319-1324.

248. Nakatani K, Takao M, Nishioka J, Yasuda K, Noma K, Hayashi A, Wada H, Nobori T. Association of epidermal growth factor receptor mutations in lung cancer with chemosensitivity to gefitinib in isolated cancer cells from Japanese patients. European journal of cancer care. 2007; 16:263-267.

249. Murray S, Papadopoulou E, Nasioulas G. (2012). Frequency of EGFR mutations in Greek non-small-cell lung cancer (NSCLC) patients. ASCO Annual Meeting Proceedings, pp. e21141.

250. Neal JW, Heist RS, Fidias P, Temel JS, Huberman M, Marcoux JP, Muzikansky A, Lynch TJ, Sequist LV. Cetuximab monotherapy in patients with advanced non-small cell lung cancer after prior epidermal growth factor receptor tyrosine kinase inhibitor therapy. Journal of Thoracic Oncology. 2010; 5:1855-1858.

251. Niho S, Kubota K, Goto K, Yoh K, Ohmatsu H, Kakinuma R, Saijo N, Nishiwaki Y. First-line single agent treatment with gefitinib in patients with advanced non–small-cell lung cancer: A phase II study. Journal of Clinical Oncology. 2006; 24:64-69.

252. Ninomiya H, Hiramatsu M, Inamura K, Nomura K, Okui M, Miyoshi T, Okumura S, Satoh Y, Nakagawa K, Nishio M. Correlation between morphology and EGFR mutations

in lung adenocarcinomas: significance of the micropapillary pattern and the hobnail cell type. Lung cancer (Amsterdam, Netherlands). 2009; 63:235-240.

253. Jingying N, Na Q, Jinghui W, Xinjie Y, ZHANG H, Yuhua W, Jialin L, ZHANG Q, ZHANG S. Clinical Effects for Patients with Recurrent Advanced Non-small Cell Lung Cancer Treated with Icotinib Hydrochloride. Chinese Journal of Lung Cancer. 2013; 16.

254. Noronha V, Prabhash K, Thavamani A, Chougule A, Purandare N, Joshi A, Sharma R, Desai S, Jambekar N, Dutt A. EGFR mutations in Indian lung cancer patients: clinical correlation and outcome to EGFR targeted therapy. PloS one. 2013; 8:e61561.

255. Nose N, Sugio K, Oyama T, Nozoe T, Uramoto H, Iwata T, Onitsuka T, Yasumoto K. Association between estrogen receptor-β expression and epidermal growth factor receptor mutation in the postoperative prognosis of adenocarcinoma of the lung. Journal of Clinical Oncology. 2009; 27:411-417.

256. Ohtsuka K, Ohnishi H, Fujiwara M, Kishino T, Matsushima S, Furuyashiki G, Takei H, Koshiishi Y, Goya T, Watanabe T. Abnormalities of epidermal growth factor receptor in lung squamous-cell carcinomas, adenosquamous carcinomas, and large-cell carcinomas. Cancer. 2007; 109:741-750.

257. Okada H, Anayama T, Kume M, Hirohashi K, Miyazaki R, Matsumoto M, Orihashi K. Comparison of epidermal growth factor receptor mutation analysis results between surgically resected primary lung cancer and metastatic lymph nodes obtained by endobronchial ultrasound-guided transbronchial needle aspiration. Thoracic Cancer. 2012; 3:262-268.

258. Okudela K, Woo T, Yazawa T, Ogawa N, Tajiri M, Masuda M, Kitamura H. Significant association between EGFR-mutated lung adenocarcinoma and past illness from gastric cancer or uterine myoma: Its implication in carcinogenesis. Lung cancer (Amsterdam, Netherlands). 2009; 66:287-291.

259. Onitsuka T, Uramoto H, Ono K, Takenoyama M, Hanagiri T, Oyama T, Izumi H, Kohno K, Yasumoto K. Comprehensive molecular analyses of lung adenocarcinoma with regard to the epidermal growth factor receptor, K-ras, MET, and hepatocyte growth factor status. Journal of Thoracic Oncology. 2010; 5:591-596.

260. Oshita F, Miyagi Y, Honda T, Murakami S, Kondo T, Saito H, Noda K, Yokose T, Kameda Y, Sakuma Y. Prospective study of second-line chemotherapy for non-small cell lung cancer selected according to EGFR gene status. Journal of experimental therapeutics & oncology. 2010; 8:313-319.

261. Otani H, Toyooka S, Soh J, Yamamoto H, Suehisa H, Kobayashi N, Gobara H, Mimura H, Kiura K, Sano Y. Detection of EGFR gene mutations using the wash fluid of CT-guided biopsy needle in NSCLC patients. Journal of Thoracic Oncology. 2008; 3:472-476.

262. Pai R, Nehru AG, Singh A, Bhatt AD, Suganthi S, Korula A, Chacko RT. (2011). Activating EGFR mutations among patients with non-small cell lung cancer from India. Association of Molecular Pathology), pp. 763.

263. Paik PK, Arcila ME, Fara M, Sima CS, Miller VA, Kris MG, Ladanyi M, Riely GJ. Clinical characteristics of patients with lung adenocarcinomas harboring BRAF mutations. Journal of clinical oncology. 2011; 29:2046-2051.

264. Paik PK, Johnson ML, D'Angelo SP, Sima CS, Ang D, Dogan S, Miller VA, Ladanyi M, Kris MG, Riely GJ. Driver mutations determine survival in smokers and never-smokers with stage IIIB/IV lung adenocarcinomas. Cancer. 2012; 118:5840-5847.

265. Pallis AG, Voutsina A, Kentepozidis N, Giassas S, Papakotoulas P, Agelaki S, Tryfonidis K, Kotsakis A, Vamvakas L, Vardakis N. A phase II trial of erlotinib as front-line treatment in clinically selected patients with non–small-cell lung cancer. Clinical lung cancer. 2012; 13:129-135.

266. Pan Q, Pao W, Ladanyi M. Rapid polymerase chain reaction-based detection of epidermal growth factor receptor gene mutations in lung adenocarcinomas. The Journal of molecular diagnostics: JMD. 2005; 7:396-403.

267. Park S, Holmes-Tisch AJ, Shim YM, Kim J, Kim HS, Lee J, Park YH, Ahn JS, Park K, Jänne PA. Discordance of molecular biomarkers associated with epidermal growth factor receptor pathway between primary tumors and lymph node metastasis in non-small cell lung cancer. Journal of Thoracic Oncology. 2009; 4:809-815.

268. Park JH, Lee S-H, Keam B, Kim TM, Kim D-W, Yang S-C, Kim YW, Heo DS. EGFR mutations as a predictive marker of cytotoxic chemotherapy. Lung cancer (Amsterdam, Netherlands). 2012; 77:433-437.

269. Zhao J, Peng Q, Yang B, Lu D. [High-resolution melting analysis for the detection of EGFR mutations in circulating DNA of lung cancer patients]. National Medical Journal of China. 2011; 91:674-678.

270. Pennycuick A, Simpson T, Crawley D, Lal R, Santis G, Cane P, Tobal K, Spicer J. Routine EGFR and KRAS Mutation analysis using COLD-PCR in non-small cell lung cancer. International journal of clinical practice. 2012; 66:748-752.

271. Pham D, Kris MG, Riely GJ, Sarkaria IS, McDonough T, Chuai S, Venkatraman ES, Miller VA, Ladanyi M, Pao W. Use of cigarette-smoking history to estimate the likelihood of mutations in epidermal growth factor receptor gene exons 19 and 21 in lung adenocarcinomas. Journal of Clinical Oncology. 2006; 24:1700-1704.

272. Pietanza MC, Gadgeel SM, Dowlati A, Lynch TJ, Salgia R, Rowland Jr KM, Wertheim MS, Price KA, Riely GJ, Azzoli CG, Miller VA, Krug LM, Kris MG, Beumer JH, Tonda M, Mitchell B, et al. Phase II study of the multitargeted tyrosine kinase inhibitor XL647 in patients with non-small-cell lung cancer. (United States: International Association for the Study of Lung Cancer (PO Box 6511, Mail Stop

8111, Aurora, Colorado 80045-0511, United States)), pp. 856-865.
273. Pietanza MC, Gadgeel SM, Dowlati A, Lynch TJ, Salgia R, Rowland Jr KM, Wertheim MS, Price KA, Riely GJ, Azzoli CG, Miller VA, Krug LM, Kris MG, Beumer JH, Tonda M, Mitchell B, et al. (2012). Phase II study of the multitargeted tyrosine kinase inhibitor XL647 in patients with non-small-cell lung cancer. (United States: International Association for the Study of Lung Cancer (PO Box 6511, Mail Stop 8111, Aurora, Colorado 80045-0511, United States)), pp. 856-865.
274. Porta R, Sanchez-Torres J, Paz-Ares L, Massuti B, Reguart N, Mayo C, Lianes P, Queralt C, Guillem V, Salinas P. Brain metastases from lung cancer responding to erlotinib: the importance of EGFR mutation. European Respiratory Journal. 2011; 37:624-631.
275. Price KA, Azzoli CG, Krug LM, Pietanza MC, Rizvi NA, Pao W, Kris MG, Riely GJ, Heelan RT, Arcila ME. Phase II trial of gefitinib and everolimus in advanced non-small cell lung cancer. Journal of Thoracic Oncology. 2010; 5:1623-1629.
276. Provencio M, Molina MA, Rosell R. Screening for Epidermal Growth Factor Receptor Mutations in Lung Cancer and Tyrosine-Kinase Inhibitors. European Journal of Clinical & Medical Oncology. 2011; 3.
277. Pugh TJ, Bebb G, Barclay L, Sutcliffe M, Fee J, Salski C, O'Connor R, Ho C, Murray N, Melosky B. Correlations of EGFR mutations and increases in EGFR and HER2 copy number to gefitinib response in a retrospective analysis of lung cancer patients. BMC cancer. 2007; 7:1.
278. Raez LE, Ochoa RE, Villasboas JC, Walker G, Gomez JE, Belalcazar A, Danenberg K, Hu JJ, Santos ES. (2011). Disparities in outcomes in Hispanic patients with non-small cell lung cancer (NSCLC): Evaluation of differences of predictive biomarker expression. American Society of Clinical Oncology).
279. Ragusa M, Vannucci J, Ludovini V, Bianconi F, Treggiari S, Tofanetti FR, Flacco A, Colella R, Sidoni A, Crinò L. Impact of Epidermal Growth Factor Receptor and KRAS Mutations on Clinical Outcome in Resected Non–Small Cell Lung Cancer Patients. American journal of clinical oncology. 2014; 37:343-349.
280. Rahman S, Kondo N, Yoneda K, Takuwa T, Hashimoto M, Orui H, Okumura Y, Tanaka F, Kumamoto K, Mostafa MG. Frequency of epidermal growth factor receptor mutations in Bangladeshi patients with adenocarcinoma of the lung. International journal of clinical oncology. 2014; 19:45-49.
281. Ready N, Jänne PA, Bogart J, DiPetrillo T, Garst J, Graziano S, Gu L, Wang X, Green MR, Vokes EE. Chemoradiotherapy and gefitinib in stage III non-small cell lung cancer with epidermal growth factor receptor and KRAS mutation analysis: cancer and leukemia group B (CALEB) 30106, a CALGB-stratified phase II trial. Journal of Thoracic Oncology. 2010; 5:1382-1390.
282. Reinersman JM, Johnson ML, Riely GJ, Chitale DA, Nicastri AD, Soff GA, Schwartz AG, Sima CS, Ayalew G, Lau C. Frequency of EGFR and KRAS mutations in lung adenocarcinomas in African Americans. Journal of Thoracic Oncology. 2011; 6:28-31.
283. Reinmuth N, Jauch A, Xu EC, Muley T, Granzow M, Hoffmann H, Dienemann H, Herpel E, Schnabel PA, Herth FJ. Correlation of EGFR mutations with chromosomal alterations and expression of EGFR, ErbB3 and VEGF in tumor samples of lung adenocarcinoma patients. Lung cancer (Amsterdam, Netherlands). 2008; 62:193-201.
284. Rekhtman N, Brandt SM, Sigel CS, Friedlander MA, Riely GJ, Travis WD, Zakowski MF, Moreira AL. Suitability of thoracic cytology for new therapeutic paradigms in non-small cell lung carcinoma: high accuracy of tumor subtyping and feasibility of EGFR and KRAS molecular testing. Journal of Thoracic Oncology. 2011; 6:451-458.
285. Rekhtman N, Ang DC, Riely GJ, Ladanyi M, Moreira AL. KRAS mutations are associated with solid growth pattern and tumor-infiltrating leukocytes in lung adenocarcinoma. Modern Pathology. 2013; 26:1307-1319.
286. Ren G-j, Zhao Y-y, Zhu Y-j, Xiao Y, Xu J-s, Shan B, Zhang L. Tumor gene mutations and messenger RNA expression: correlation with clinical response to icotinib hydrochloride in non-small cell lung cancer. Chinese Medical Journal-Beijing. 2011; 124:19.
287. Ren RX, Li JY, Li XF, Chen X, Ren SX, Zhou CC. (2012). Detection of epidermal growth factor receptor mutations in small specimens of non-small cell lung cancer by amplification refractory mutation system. (China: Shanghai Jiao Tong University (2200 Xietu Rd no.25, Shanghai 200032, China)), pp. 929-935.
288. Riely GJ, Pao W, Pham D, Li AR, Rizvi N, Venkatraman ES, Zakowski MF, Kris MG, Ladanyi M, Miller VA. Clinical course of patients with non–small cell lung cancer and epidermal growth factor receptor exon 19 and exon 21 mutations treated with gefitinib or erlotinib. Clinical cancer research. 2006; 12:839-844.
289. Righi L, Cuccurullo A, Vatrano S, Cappia S, Giachino D, De Giuli P, Ardine M, Novello S, Volante M, Scagliotti GV. Detection and characterization of classical and “uncommon” exon 19 Epidermal Growth Factor Receptor mutations in lung cancer by pyrosequencing. BMC cancer. 2013; 13:1.
290. Rosell R, Moran T, Queralt C, Porta R, Cardenal F, Camps C, Majem M, Lopez-Vivanco G, Isla D, Provencio M. Screening for epidermal growth factor receptor mutations in lung cancer. New England Journal of Medicine. 2009; 361:958-967.
291. Sahoo R, Babu VC, Okaly GVP, Rao S, Nargund A, Venkataswamy E, Rao R, Kumar BA. Screening for EGFR mutations in lung cancer, a report from India. Lung cancer (Amsterdam, Netherlands). 2011; 73:316-319.

292. Saieg MA, Geddie WR, Boerner SL, Tsao M, Hwang D, Wei C, Kamel Reid S, Da Cunha Santos G. (2011). EGFR mutation analysis in cytological samples: A retrospective study of 248 lung cancer cases. Wiley Blackwell), pp. 43-44.
293. Sakuma Y, Matsukuma S, Yoshihara M, Nakamura Y, Noda K, Nakayama H, Kameda Y, Tsuchiya E, Miyagi Y. Distinctive evaluation of nonmucinous and mucinous subtypes of bronchioloalveolar carcinomas in EGFR and K-ras gene-mutation analyses for Japanese lung adenocarcinomas. American journal of clinical pathology. 2007; 128:100-108.
294. Sartori G, Cavazza A, Sgambato A, Marchioni A, Barbieri F, Longo L, Bavieri M, Murer B, Meschiari E, Tamberi S. EGFR and K-ras mutations along the spectrum of pulmonary epithelial tumors of the lung and elaboration of a combined clinicopathologic and molecular scoring system to predict clinical responsiveness to EGFR inhibitors. American journal of clinical pathology. 2009; 131:478-489.
295. Sasaki H, Shimizu S, Endo K, Takada M, Kawahara M, Tanaka H, Matsumura A, Iuchi K, Haneda H, Suzuki E. EGFR and erbB2 mutation status in Japanese lung cancer patients. International journal of cancer. 2006; 118:180-184.
296. Sasaki H, Endo K, Konishi A, Takada M, Kawahara M, Iuchi K, Matsumura A, Okumura M, Tanaka H, Kawaguchi T. EGFR mutation status in Japanese lung cancer patients: genotyping analysis using LightCycler. Clinical Cancer Research. 2005; 11:2924-2929.
297. Sasaki H, Endo K, Mizuno K, Yano M, Fukai I, Yamakawa Y, Fujii Y. (2006). EGFR mutation status and prognosis for gefitinib treatment in Japanese lung cancer [3]. (Ireland: Elsevier Ireland Ltd), pp. 135-136.
298. Sasaki H, Endo K, Takada M, Kawahara M, Kitahara N, Tanaka H, Okumura M, Matsumura A, Iuchi K, Kawaguchi T, Kawano O, Yukiue H, Yokoyama T, Yano M, Fujii Y. EGFR exon 20 insertion mutation in Japanese lung cancer. Lung cancer (Amsterdam, Netherlands). 2007; 58:324-328.
299. Sasaki H, Endo K, Yukiue H, Kobayashi Y, Yano M, Fujii Y. (2007). Mutation of epidermal growth factor receptor gene in adenosquamous carcinoma of the lung. (Ireland: Elsevier Ireland Ltd), pp. 129-130.
300. Sasaki H, Kawano O, Endo K, Yukiue H, Yano M, Fujii Y. EGFRvIII mutation in lung cancer correlates with increased EGFR copy number. Oncology reports. 2007; 17:319-323.
301. Sasaki H, Endo K, Takada M, Kawahara M, Tanaka H, Kitahara N, Matsumura A, Iuchi K, Kawaguchi T, Okuda K, Kawano O, Yukiue H, Yokoyama T, Yano M, Fujii Y. EGFR polymorphism of the kinase domain in Japanese lung cancer. The Journal of surgical research. 2008; 148:260-263.
302. Sasaki H, Hikosaka Y, Okuda K, Kawano O, Yukiue H, Yano M, Fujii Y. MEK1 gene mutation in Japanese lung adenocarcinoma patients. Molecular medicine reports. 2009; 2:153-155.
303. Sasaki T, Koivunen J, Ogino A, Yanagita M, Nikiforow S, Zheng W, Lathan C, Marcoux JP, Du J, Okuda K, Capelletti M, Shimamura T, Ercan D, Stumpfova M, Xiao Y, Weremowicz S, et al. A novel ALK secondary mutation and EGFR signaling cause resistance to ALK kinase inhibitors. Cancer research. 2011; 71:6051-6060.
304. Savic S, Tapia C, Grilli B, Rufle A, Bihl M, de Vito Barascud A, Herzog M, Terracciano L, Baty F, Bubendorf L. Comprehensive epidermal growth factor receptor gene analysis from cytological specimens of non-small-cell lung cancers. British journal of cancer. 2008; 98:154-160.
305. Schmid K, Oehl N, Wrba F, Pirker R, Pirker C, Filipits M. EGFR/KRAS/BRAF mutations in primary lung adenocarcinomas and corresponding locoregional lymph node metastases. Clinical Cancer Research. 2009; 15:4554-4560.
306. Schneider C-P, Heigener D, Schott-von-Römer K, Gütz S, Laack E, Digel W, Guschall W-R, Franke A, Bodenstein H, Schmidtgen C. Epidermal growth factor receptor-related tumor markers and clinical outcomes with erlotinib in non-small cell lung cancer: an analysis of patients from german centers in the TRUST study. Journal of Thoracic Oncology. 2008; 3:1446-1453.
307. Scoccianti C, Vesin A, Martel G, Olivier M, Brambilla E, Timsit J-F, Tavecchio L, Brambilla C, Field JK, Hainaut P. Prognostic value of TP53, KRAS and EGFR mutations in nonsmall cell lung cancer: the EUELC cohort. European Respiratory Journal. 2012; 40:177-184.
308. Sekine A, Kato T, Hagiwara E, Shinohara T, Komagata T, Iwasawa T, Satoh H, Tamura K, Kasamatsu T, Hayashihara K. Metastatic brain tumors from non-small cell lung cancer with EGFR mutations: distinguishing influence of exon 19 deletion on radiographic features. Lung cancer (Amsterdam, Netherlands). 2012; 77:64-69.
309. Sequist LV, Martins RG, Spigel D, Grunberg SM, Spira A, Jänne PA, Joshi VA, McCollum D, Evans TL, Muzikansky A. First-line gefitinib in patients with advanced non–small-cell lung cancer harboring somatic EGFR mutations. Journal of Clinical Oncology. 2008; 26:2442-2449.
310. Sequist LV, Gettinger S, Senzer NN, Martins RG, Jänne PA, Lilenbaum R, Gray JE, Iafrate AJ, Katayama R, Hafeez N. Activity of IPI-504, a novel heat-shock protein 90 inhibitor, in patients with molecularly defined non–small-cell lung cancer. Journal of Clinical Oncology. 2010:JCO. 2010.2030. 8338.
311. Sequist LV, Besse B, Lynch TJ, Miller VA, Wong KK, Gitlitz B, Eaton K, Zacharchuk C, Freyman A, Powell C. Neratinib, an irreversible pan-ErbB receptor tyrosine kinase inhibitor: Results of a phase II trial in patients with advanced non–small-cell lung cancer. Journal of clinical oncology. 2010; 28:3076-3083.
312. Sequist LV, Heist RS, Shaw AT, Fidias P, Rosovsky R, Temel JS, Lennes IT, Digumarthy S, Waltman BA, Bast E,

Tammireddy S, Morrissey L, Muzikansky A, Goldberg SB, Gainor J, Channick CL, et al. Implementing multiplexed genotyping of non-small-cell lung cancers into routine clinical practice. Annals of oncology: official journal of the European Society for Medical Oncology / ESMO. 2011; 22:2616-2624.

313. Shaw AT, Yeap BY, Mino-Kenudson M, Digumarthy SR, Costa DB, Heist RS, Solomon B, Stubbs H, Admane S, McDermott U. Clinical features and outcome of patients with non–small-cell lung cancer who harbor EML4-ALK. Journal of clinical oncology. 2009; 27:4247-4253.

314. Shi C, Han B, Gu A, Xiong L, Shen J. [Role of erlotinib 100 mg/d in the treatment of advanced adenocarcinoma lung cancer failed to previous chemotherapy]. Chinese journal of lung cancer. 2011; 14:529-533.

315. Shiau CJ, Babwah J, Da Cunha Santos G, Boerner SL, Geddie WR, Kamel-Reid S, Wei C, Hwang DM, Tsao MS. (2012). Selection of samples for epidermal growth factor receptor (EGFR) mutation analysis in non-squamous non-small cell lung carcinoma. Nature Publishing Group), pp. 490A-491A.

316. Shigematsu H, Lin L, Takahashi T, Nomura M, Suzuki M, Wistuba II, Fong KM, Lee H, Toyooka S, Shimizu N. Clinical and biological features associated with epidermal growth factor receptor gene mutations in lung cancers. Journal of the National Cancer Institute. 2005; 97:339-346.

317. Shigematsu H, Gazdar A. (2005). The epidemiology of EGFR mutations. (United Kingdom: ADIS International Ltd), pp. 4-8.

318. Shigematsu H, Gazdar AF. Somatic mutations of epidermal growth factor receptor signaling pathway in lung cancers. International journal of cancer Journal international du cancer. 2006; 118:257-262.

319. Shigematsu H, Takahashi T, Nomura M, Majmudar K, Suzuki M, Lee H, Wistuba, II, Fong KM, Toyooka S, Shimizu N, Fujisawa T, Minna JD, Gazdar AF. Somatic mutations of the HER2 kinase domain in lung adenocarcinomas. Cancer research. 2005; 65:1642-1646.

320. Shih JY, Gow CH, Yu CJ, Yang CH, Chang YL, Tsai MF, Hsu YC, Chen KY, Su WP, Yang PC. Epidermal growth factor receptor mutations in needle biopsy/aspiration samples predict response to gefitinib therapy and survival of patients with advanced nonsmall cell lung cancer. International journal of cancer. 2006; 118:963-969.

321. Shingyoji M, Kageyama H, Sakaida T, Nakajima T, Matsui Y, Itakura M, Iuchi T, Yokoi S, Kimura H, Iizasa T. Detection of epithelial growth factor receptor mutations in cerebrospinal fluid from patients with lung adenocarcinoma suspected of neoplastic meningitis. Journal of Thoracic Oncology. 2011; 6:1215-1220.

322. Shiozawa T, Ishii G, Goto K, Nagai K, Mimaki S, Ono S, Niho S, Fujii S, Ohe Y, Tsuchihara K. Clinicopathological characteristics of EGFR mutated adenosquamous carcinoma of the lung. Pathology international. 2013; 63:77-84.

323. Skov BG, Hogdall E, Clementsen P, Krasnik M, Larsen KR, Sorensen JB, Mellemgaard A. (2012). Population based study of the prevalence of epidermal growth factor receptor (EGFR) mutations in non-small cell lung cancer (NSCLC). Wiley Blackwell), pp. 14.

324. Smits AJ, Kummer JA, Hinrichs JW, Herder GJ, Scheidel-Jacobse KC, Jiwa NM, Ruijter TEG, Nooijen PT, Looijen-Salamon MG, Ligtenberg MJ. EGFR and KRAS mutations in lung carcinomas in the Dutch population: increased EGFR mutation frequency in malignant pleural effusion of lung adenocarcinoma. Cellular oncology. 2012; 35:189-196.

325. Smouse JH, Cibas ES, Jänne PA, Joshi VA, Zou KH, Lindeman NI. EGFR mutations are detected comparably in cytologic and surgical pathology specimens of nonsmall cell lung cancer. Cancer cytopathology. 2009; 117:67-72.

326. Sone T, Kasahara K, Kimura H, Nishio K, Mizuguchi M, Nakatsumi Y, Shibata K, Waseda Y, Fujimura M, Nakao S. Comparative analysis of epidermal growth factor receptor mutations and gene amplification as predictors of gefitinib efficacy in Japanese patients with nonsmall cell lung cancer. Cancer. 2007; 109:1836-1844.

327. Song L, Rawal B, Nemeth JA, Haura EB. (2011). JAK1 activates STAT3 activity in non-small-cell lung cancer cells and IL-6 neutralizing antibodies can suppress JAK1-STAT3 signaling. (United States: American Association for Cancer Research Inc. (615 Chestnut Street, 17th Floor, Philadelphia PA 19106-3483, United States)), pp. 481-494.

328. Song Z, Yu X, Cai J, Shao L, Lin B, He C, Zhang B, Zhang Y. (2013). Efficacy of icotinib for advanced non-small cell lung cancer patients with EGFR status identified. (China: Chinese Journal of Lung Cancer (228 Nanjing Rd, Tianjin 300020, China)), pp. 138-143.

329. Song YY, Gu AQ, Han B. Expression of p21, p53 and c-erbB-2 in tissues of non-small cell lung cancer and its clinical significance. (China: Shanghai Jiaotong Daxue (Shanghai Jiaotong University) (1954 Huasha Lu, Shanghai 200030, China)), pp. 295-298.

330. Sonobe M, Manabe T, Wada H, Tanaka F. Mutations in the epidermal growth factor receptor gene are linked to smoking-independent, lung adenocarcinoma. British journal of cancer. 2005; 93:355-363.

331. Sonobe M, Nakagawa M, Takenaka K, Katakura H, Adachi M, Yanagihara K, Otake Y, Wada H, Tanaka F. Influence of epidermal growth factor receptor (EGFR) gene mutations on the expression of EGFR, phosphoryl-Akt, and phosphoryl-MAPK, and on the prognosis of patients with non-small cell lung cancer. Journal of surgical oncology. 2007; 95:63-69.

332. Sonobe M, Kobayashi M, Ishikawa M, Kikuchi R, Nakayama E, Takahashi T, Menju T, Takenaka K, Miyahara R, Huang C-L. Impact of KRAS and EGFR gene mutations on recurrence and survival in patients with surgically

resected lung adenocarcinomas. Annals of surgical oncology. 2012; 19:347-354.

333. Soung YH, Lee JW, Kim SY, Seo SH, Park WS, Nam SW, Song SY, Han JH, Park CK, Lee JY. Mutational analysis of EGFR and K-RAS genes in lung adenocarcinomas. Virchows Archiv. 2005; 446:483-488.

334. Spigel DR, Burris HA, Greco FA, Shipley DL, Friedman EK, Waterhouse DM, Whorf RC, Mitchell RB, Daniel DB, Zangmeister J. Randomized, double-blind, placebo-controlled, phase II trial of sorafenib and erlotinib or erlotinib alone in previously treated advanced non–small-cell lung cancer. Journal of clinical oncology. 2011; 29:2582-2589.

335. Sriram KB, Tan ME, Savarimuthu SM, Wright CM, Relan V, Stockwell RE, Clarke BE, Duhig EE, Yang IA, Bowman RV. Screening for activating EGFR mutations in surgically resected nonsmall cell lung cancer. European Respiratory Journal. 2011; 38:903-910.

336. Sriuranpong V, Chantranuwat C, Huapai N, Chalermchai T, Leungtaweeboon K, Lertsanguansinchai P, Voravud N, Mutirangura A. High frequency of mutation of epidermal growth factor receptor in lung adenocarcinoma in Thailand. Cancer letters. 2006; 239:292-297.

337. Suehisa H, Toyooka S, Hotta K, Uchida A, Soh J, Fujiwara Y, Matsuo K, Ouchida M, Takata M, Kiura K. Epidermal growth factor receptor mutation status and adjuvant chemotherapy with uracil-tegafur for adenocarcinoma of the lung. Journal of Clinical Oncology. 2007; 25:3952-3957.

338. Sueoka N, Sato A, Eguchi H, Komiya K, Sakuragi T, Mitsuoka M, Satoh T, Hayashi S, Nakachi K, Sueoka E. Mutation profile of EGFR gene detected by denaturing high-performance liquid chromatography in Japanese lung cancer patients. Journal of cancer research and clinical oncology. 2007; 133:93-102.

339. yoHeI MIyAMAe KK, tAKuyA ArAKI MK. Correlation between computed tomography findings and epidermal growth factor receptor and KRAS gene mutations in patients with pulmonary adenocarcinoma. Oncology reports. 2011; 26:1205-1211.

340. Sugio K, Uramoto H, Ono K, Oyama T, Hanagiri T, Sugaya M, Ichiki Y, So T, Nakata S, Morita M. Mutations within the tyrosine kinase domain of EGFR gene specifically occur in lung adenocarcinoma patients with a low exposure of tobacco smoking. British journal of cancer. 2006; 94:896-903.

341. Sugio K, Uramoto H, Onitsuka T, Mizukami M, Ichiki Y, Sugaya M, Yasuda M, Takenoyama M, Oyama T, Hanagiri T. Prospective phase II study of gefitinib in non-small cell lung cancer with epidermal growth factor receptor gene mutations. Lung cancer (Amsterdam, Netherlands). 2009; 64:314-318.

342. Sun L, Luan H, Zang F, Wang M, Dong N, Guo Y, Sun B, Zhan Z. [Relationship between EGFR and K-ras mutations and clinicopathological characteristics and response to erlotinib treatment in 301 Chinese patients with non-small cell lung cancer]. Chinese journal of oncology. 2010; 32:667-670.

343. Sun H-b, Zheng Y, Ou W, Fang Q, Li P, Ye X, Zhang B-b, Yang H, Wang S-y. Association between hormone receptor expression and epidermal growth factor receptor mutation in patients operated on for non-small cell lung cancer. The Annals of thoracic surgery. 2011; 91:1562-1567.

344. Sun L, Zhang Q, Luan H, Zhan Z, Wang C, Sun B. Comparison of KRAS and EGFR gene status between primary non-small cell lung cancer and local lymph node metastases: implications for clinical practice. Journal of Experimental & Clinical Cancer Research. 2011; 30:1.

345. Sun H-b, Ou W, Li Y, Fang Q, Qin J, Zhang L, Wang S-y. Epidermal Growth Factor Receptor Mutation Status and Adjuvant Chemotherapy in Resected Advanced Non–Small-Cell Lung Cancer. Clinical lung cancer. 2013; 14:376-382.

346. Sun P-L, Seol H, Lee HJ, Yoo SB, Kim H, Xu X, Jheon S, Lee C-T, Lee J-S, Chung J-H. High incidence of EGFR mutations in Korean men smokers with no intratumoral heterogeneity of lung adenocarcinomas: correlation with histologic subtypes, EGFR/TTF-1 expressions, and clinical features. Journal of Thoracic Oncology. 2012; 7:323-330.

347. Sun J-M, Hwang DW, Ahn JS, Ahn M-J, Park K. Prognostic and predictive value of KRAS mutations in advanced non-small cell lung cancer. PloS one. 2013; 8:e64816.

348. Suzuki M, Shigematsu H, Hiroshima K, Iizasa T, Nakatani Y, Minna JD, Gazdar AF, Fujisawa T. Epidermal growth factor receptor expression status in lung cancer correlates with its mutation. Human pathology. 2005; 36:1127-1134.

349. Suzuki S, Dobashi Y, Sakurai H, Nishikawa K, Hanawa M, Ooi A. Protein overexpression and gene amplification of epidermal growth factor receptor in nonsmall cell lung carcinomas. An immunohistochemical and fluorescence in situ hybridization study. Cancer. 2005; 103:1265-1273.

350. Suzuki M, Shigematsu H, Nakajima T, Kubo R, Motohashi S, Sekine Y, Shibuya K, Iizasa T, Hiroshima K, Nakatani Y. Synchronous Alterations of Wnt and Epidermal Growth Factor Receptor Signaling Pathways through Aberrant Methylation and Mutation in Non–Small Cell Lung Cancer. Clinical Cancer Research. 2007; 13:6087-6092.

351. Suzuki M, Wada H, Yoshino M, Tian L, Shigematsu H, Suzuki H, Alaa M, Tamura H, Fujiwara T, Nagato K. Molecular characterization of chronic obstructive pulmonary disease-related non-small cell lung cancer through aberrant methylation and alterations of EGFR signaling. Annals of surgical oncology. 2010; 17:878-888.

352. Taga M, Mechanic LE, Hagiwara N, Vähäkangas KH, Bennett WP, Alavanja MC, Welsh JA, Khan MA, Lee A, Diasio R. EGFR somatic mutations in lung tumors: radon exposure and passive smoking in former-and never-smoking

US women. Cancer Epidemiology Biomarkers & Prevention. 2012; 21:988-992.
353. Takahashi T, Sonobe M, Kobayashi M, Yoshizawa A, Menju T, Nakayama E, Mino N, Iwakiri S, Sato K, Miyahara R. Clinicopathologic features of non-small-cell lung cancer with EML4–ALK fusion gene. Annals of surgical oncology. 2010; 17:889-897.
354. Takamochi K, Oh S, Matsuoka J, Suzuki K. Clonality status of multifocal lung adenocarcinomas based on the mutation patterns of EGFR and K-ras. Lung cancer (Amsterdam, Netherlands). 2012; 75:313-320.
355. Takano T, Ohe Y, Sakamoto H, Tsuta K, Matsuno Y, Tateishi U, Yamamoto S, Nokihara H, Yamamoto N, Sekine I. Epidermal growth factor receptor gene mutations and increased copy numbers predict gefitinib sensitivity in patients with recurrent non–small-cell lung cancer. Journal of Clinical Oncology. 2005; 23:6829-6837.
356. Takeda M, Okamoto I, Sakai K, Kawakami H, Nishio K, Nakagawa K. Clinical outcome for EML4-ALK-positive patients with advanced non-small-cell lung cancer treated with first-line platinum-based chemotherapy. Annals of oncology. 2012; 23:2931-2936.
357. Takeshita M, Koga T, Takayama K, Yano T, Maehara Y, Nakanishi Y, Sueishi K. Alternative efficacy-predicting markers for paclitaxel instead of CHFR in non-small-cell lung cancer. Cancer biology & therapy. 2010; 10:933-941.
358. San Tam IY, Chung LP, Suen WS, Wang E, Wong MC, Ho KK, Lam WK, Chiu SW, Girard L, Minna JD. Distinct epidermal growth factor receptor and KRAS mutation patterns in non–small cell lung cancer patients with different tobacco exposure and clinicopathologic features. Clinical Cancer Research. 2006; 12:1647-1653.
359. Tanaka T, Matsuoka M, Sutani A, Gemma A, Maemondo M, Inoue A, Okinaga S, Nagashima M, Oizumi S, Uematsu K. Frequency of and variables associated with the EGFR mutation and its subtypes. International Journal of Cancer. 2010; 126:651-655.
360. Taron M, Ichinose Y, Rosell R, Mok T, Massuti B, Zamora L, Mate JL, Manegold C, Ono M, Queralt C. Activating mutations in the tyrosine kinase domain of the epidermal growth factor receptor are associated with improved survival in gefitinib-treated chemorefractory lung adenocarcinomas. Clinical Cancer Research. 2005; 11:5878-5885.
361. Tiseo M, Rossi G, Capelletti M, Sartori G, Spiritelli E, Marchioni A, Bozzetti C, De Palma G, Lagrasta C, Campanini N. Predictors of gefitinib outcomes in advanced non-small cell lung cancer (NSCLC): study of a comprehensive panel of molecular markers. Lung cancer (Amsterdam, Netherlands). 2010; 67:355-360.
362. Tochigi N, Dacic S, Nikiforova M, Cieply KM and Yousem SA. Adenosquamous Carcinoma of the Lung. American journal of clinical pathology. 2011; 135:783-789.
363. Togashi Y, Masago K, Kubo T, Sakamori Y, Kim YH, Hatachi Y, Fukuhara A, Mio T, Togashi K, Mishima M. Association of diffuse, random pulmonary metastases, including miliary metastases, with epidermal growth factor receptor mutations in lung adenocarcinoma. Cancer. 2011; 117:819-825.
364. Tokumo M, Toyooka S, Kiura K, Shigematsu H, Tomii K, Aoe M, Ichimura K, Tsuda T, Yano M, Tsukuda K. The relationship between epidermal growth factor receptor mutations and clinicopathologic features in non–small cell lung cancers. Clinical cancer research. 2005; 11:1167-1173.
365. Tomizawa Y, Iijima H, Sunaga N, Sato K, Takise A, Otani Y, Tanaka S, Suga T, Saito R, Ishizuka T. Clinicopathologic significance of the mutations of the epidermal growth factor receptor gene in patients with non–small cell lung cancer. Clinical cancer research. 2005; 11:6816-6822.
366. De Pas T, Toffalorio F, Manzotti M, Fumagalli C, Spitaleri G, Catania C, Delmonte A, Giovannini M, Spaggiari L and de Braud F. Activity of epidermal growth factor receptor-tyrosine kinase inhibitors in patients with non-small cell lung cancer harboring rare epidermal growth factor receptor mutations. Journal of Thoracic Oncology. 2011; 6:1895-1901.
367. Toyooka S, Matsuo K, Shigematsu H, Kosaka T, Tokumo M, Yatabe Y, Ichihara S, Inukai M, Suehisa H, Soh J. The Impact of Sex and Smoking Status on the Mutational Spectrum of Epidermal Growth Factor Receptor Gene in Non–small Cell Lung Cancer. Clinical Cancer Research. 2007; 13:5763-5768.
368. Toyooka S, Takano T, Kosaka T, Hotta K, Matsuo K, Ichihara S, Fujiwara Y, Soh J, Otani H, Kiura K. Epidermal growth factor receptor mutation, but not sex and smoking, is independently associated with favorable prognosis of gefitinib-treated patients with lung adenocarcinoma. Cancer science. 2008; 99:303-308.
369. Martínez Rodríguez P, Felip Font E, Suriñach JM. ALK rearrangement in a selected population of advanced non small cell lung cancer patients. FISH and inmunohistochemistry diagnostic methods, prevalence and clinical outcomes. 2012.
370. Tsai T-H, Wu S-G, Chang Y-L, Wu C-T, Tsai M-F, Wei P-F, Yang C-H, Yu C-J, Yang P-C, Shih J-Y. Effusion immunocytochemistry as an alternative approach for the selection of first-line targeted therapy in advanced lung adenocarcinoma. Journal of Thoracic Oncology. 2012; 7:993-1000.
371. Tsao M-S, Sakurada A, Cutz J-C, Zhu C-Q, Kamel-Reid S, Squire J, Lorimer I, Zhang T, Liu N, Daneshmand M. Erlotinib in lung cancer—molecular and clinical predictors of outcome. New England Journal of Medicine. 2005; 353:133-144.
372. Tsao AS, Tang XM, Sabloff B, Xiao L, Shigematsu H, Roth J, Spitz M, Hong WK, Gazdar A, Wistuba I.

Clinicopathologic characteristics of the EGFR gene mutation in non–small cell lung cancer. Journal of Thoracic Oncology. 2006; 1:231-239.

373. Tsao M-S, Sakurada A, Ding K, Aviel-Ronen S, Ludkovski O, Liu N, Le Maître A, Gandara D, Johnson DH, Rigas JR. Prognostic and predictive value of EGFR tyrosine kinase domain mutation status and gene copy number for adjuvant chemotherapy in non-small cell lung cancer. Journal of thoracic oncology: official publication of the International Association for the Study of Lung Cancer. 2011; 6:139.
374. Ueno T, Toyooka S, Suda K, Soh J, Yatabe Y, Miyoshi S, Matsuo K, Mitsudomi T. Impact of age on epidermal growth factor receptor mutation in lung cancer. Lung cancer (Amsterdam, Netherlands). 2012; 78:207-211.
375. Ulivi P, Romagnoli M, Chiadini E, Casoni G-L, Capelli L, Gurioli C, Zoli W, Saragoni L, Dubini A, Tesei A. Assessment of EGFR and K-ras mutations in fixed and fresh specimens from transesophageal ultrasound-guided fine needle aspiration in non-small cell lung cancer patients. International journal of oncology. 2012; 41:147.
376. Umemura S, Tsubouchi K, Yoshioka H, Hotta K, Takigawa N, Fujiwara K, Horita N, Segawa Y, Hamada N, Takata I. Clinical outcome in patients with leptomeningeal metastasis from non-small cell lung cancer: Okayama Lung Cancer Study Group. Lung cancer (Amsterdam, Netherlands). 2012; 77:134-139.
377. Uramoto H, Sugio K, Oyama T, Ono K, Sugaya M, Yoshimatsu T, Hanagiri T, Morita M, Yasumoto K. Epidermal growth factor receptor mutations are associated with gefitinib sensitivity in non-small cell lung cancer in Japanese. Lung cancer (Amsterdam, Netherlands). 2006; 51:71-77.
378. Uramoto H, Shimokawa H, Nagata Y, Ono K, Hanagiri T. EGFR-activating mutations are not present in breast tumors of Japanese patients. Anticancer research. 2010; 30:4219-4222.
379. Usui K, Ushijima T, Tanaka Y, Tanai C, Noda H, Abe N, Horiuchi H, Ishihara T. The frequency of epidermal growth factor receptor mutation of nonsmall cell lung cancer according to the underlying pulmonary diseases. Pulmonary medicine. 2011; 2011.
380. Usui K, Ushijima T, Tanaka Y, Tanai C, Noda H, Abe N, Horiuchi H, Ishihara T. The frequency of epidermal growth factor receptor mutation of nonsmall cell lung cancer according to the underlying pulmonary diseases. (United States: Hindawi Publishing Corporation (410 Park Avenue, 15th Floor, 287 pmb, New York NY 10022, United States)).
381. Van Zandwijk N, Mathy A, Boerrigter L, Ruijter H, Tielen I, De Jong D, Baas P, Burgers S, Nederlof P. EGFR and KRAS mutations as criteria for treatment with tyrosine kinase inhibitors: retro-and prospective observations in non-small-cell lung cancer. Annals of oncology. 2007; 18:99-103.
382. Varghese AM, Sima CS, Chaft JE, Johnson ML, Riely GJ, Ladanyi M, Kris MG. Lungs don't forget: Comparison of the KRAS and EGFR mutation profile and survival of collegiate smokers and never smokers with advanced lung cancers. Journal of Thoracic Oncology. 2013; 8:123-125.
383. Vincenten J, Smit EF, Vos W, Grünberg K, Postmus PE, Heideman DA, Snijders PJ, Meijer G, Kuik J, Witte BI. Negative NKX2-1 (TTF-1) as Temporary Surrogate Marker for Treatment Selection During EGFR-Mutation Analysis in Patients with Non–Small-Cell Lung Cancer. Journal of Thoracic Oncology. 2012; 7:1522-1527.
384. Wada M, Yamamoto M, Ryuge S, Nagashima Y, Hayashi N, Maki S, Otani S, Katono K, Takakura A, Yanaihara T. Phase II study of S-1 monotherapy in patients with previously treated, advanced non-small-cell lung cancer. Cancer chemotherapy and pharmacology. 2012; 69:1005-1011.
385. Wang Z, Wu YL, Zhang GC, Zhou Q, Xu CR, Guo AL. EGFR/KRAS mutations and gefitinib therapy in Chinese NSCLC patients. Oncology Research and Treatment. 2008; 31:174-178.
386. Wang M, Zhang X, Zhang X, Zhang L, Zhong W, Xu L, Li L. [Efficacy and safety of erlotinib as monotherapy for advanced non-small cell lung cancer]. Acta Academiae Medicinae Sinicae. 2010; 32:151-156.
387. Smith G, Chadwick B, Willmore-Payne C, Bentz J. Detection of epidermal growth factor receptor gene mutations in cytology specimens from patients with non-small cell lung cancer utilising high-resolution melting amplicon analysis. Journal of clinical pathology. 2008; 61:487-493.
388. Wang F, Fu S, Tang T, Deng L, Zhang X, Li Y, Shao J. [Relationship between mutations of epidermal growth factor receptor gene and clinicopathologic features of non-small cell lung cancers]. Chinese journal of pathology. 2011; 40:664-666.
389. WANG L-y, HUANG M-j, LI L, ZHANG Y, LU Y. Pre- and post-chemotherapy mutation status of epidermal growth factor receptor gene in non-small cell lung cancer. Tumor. 2011; 31:517-521.
390. Wang F, Wang S, Wang Z, Duan J, An T, Zhao J, Bai H, Wang J. Phosphorylated EGFR expression may predict outcome of EGFR-TKIs therapy for the advanced NSCLC patients with wild-type EGFR. Journal of Experimental & Clinical Cancer Research. 2012; 31:1.
391. Wang S, An T, Duan J, Zhang L, Wu M, Zhou Q, Chen J, Zhuo M, Yang L, Wang Y, Bai H, Wang J. Alterations in EGFR and related genes following neo-adjuvant chemotherapy in Chinese patients with non-small cell lung cancer. PloS one. 2013; 8:e51021.
392. Webb S, Thomas M, Metcalf C, Segal A, Nowak AK, Bentel J, Millward M. (2009). EGFR mutation testing in NSCLC: Patterns of care and outcomes in Western Australia. (United

Kingdom: Blackwell Publishing Ltd (9600 Garsington Road, Oxford OX4 2XG, United Kingdom)), pp. 66-71.

393. Welsh JW, Komaki R, Amini A, Munsell MF, Unger W, Allen PK, Chang JY, Wefel JS, McGovern SL, Garland LL. Phase II trial of erlotinib plus concurrent whole-brain radiation therapy for patients with brain metastases from non–small-cell lung cancer. Journal of Clinical Oncology. 2013; 31:895-902.
394. Wen J, Fu J, Zhang W, Guo M. Genetic and epigenetic changes in lung carcinoma and their clinical implications. Modern pathology: an official journal of the United States and Canadian Academy of Pathology, Inc. 2011; 24:932-943.
395. Wong DW, Leung EL, So KK, Tam IY, Sihoe AD, Cheng LC, Ho KK, Au JS, Chung LP, Pik Wong M. The EML4-ALK fusion gene is involved in various histologic types of lung cancers from nonsmokers with wild-type EGFR and KRAS. Cancer. 2009; 115:1723-1733.
396. Wu C, Li LY, Wang MZ, Zhang L, Zhang XT, Zhong W, Wang SL, Wang HZ. [Gefitinib in the treatment of advanced non-small cell lung cancer with brain metastasis]. Chinese journal of oncology. 2007; 29:943-945.
397. Wu S, Gow C, Yu C, Chang Y, Yang C, Hsu Y, Shih J, Lee Y, Yang P. Frequent epidermal growth factor receptor gene mutations in malignant pleural effusion of lung adenocarcinoma. European Respiratory Journal. 2008; 32:924-930.
398. Wu Y-L, Zhong W-Z, Li L-Y, Zhang X-T, Zhang L, Zhou C-C, Liu W, Jiang B, Mu X-L, Lin J-Y. Epidermal growth factor receptor mutations and their correlation with gefitinib therapy in patients with non-small cell lung cancer: a meta-analysis based on updated individual patient data from six medical centers in mainland China. Journal of Thoracic Oncology. 2007; 2:430-439.
399. Wu CC, Hsu HY, Liu HP, Chang JWC, Chen YT, Hsieh WY, Hsieh JJ, Hsieh MS, Chen YR, Huang SF. Reversed mutation rates of KRAS and EGFR genes in adenocarcinoma of the lung in Taiwan and their implications. Cancer. 2008; 113:3199-3208.
400. Wu J-Y, Yu C-J, Yang C-H, Wu S-G, Chiu Y-H, Gow C-H, Chang Y-C, Hsu Y-C, Wei P-F, Shih J-Y. First-or second-line therapy with gefitinib produces equal survival in non–small cell lung cancer. American journal of respiratory and critical care medicine. 2008; 178:847-853.
401. Wu JY, Shih JY, Yang CH, Chen KY, Ho CC, Yu CJ, Yang PC. Second-line treatments after first-line gefitinib therapy in advanced nonsmall cell lung cancer. International Journal of Cancer. 2010; 126:247-255.
402. Wu M, Zhao J, Song SW, Zhuo M, Wang X, Bai H, Wang S, Yang L, An T, Zhang Y. EGFR mutations are associated with prognosis but not with the response to front-line chemotherapy in the Chinese patients with advanced non-small cell lung cancer. Lung cancer (Amsterdam, Netherlands). 2010; 67:343-347.
403. Wu SG, Yang CH, Yu CJ, Lee JH, Hsu YC, Chang YL, Shih JY, Yang PC. Good response to pemetrexed in patients of lung adenocarcinoma with epidermal growth factor receptor (EGFR) mutations. Lung cancer (Amsterdam, Netherlands). 2011; 72:333-339.
404. Wu JY, Yang CH, Hsu YC, Yu CJ, Chang SH, Shih JY, Yang PC. Use of cetuximab after failure of gefitinib in patients with advanced non-small-cell lung cancer. Clinical lung cancer. 2010; 11:257-263.
405. Wu JY, Wu SG, Yang CH, Chang YL, Chang YC, Hsu YC, Shih JY, Yang PC. Comparison of gefitinib and erlotinib in advanced NSCLC and the effect of EGFR mutations. Lung cancer (Amsterdam, Netherlands). 2011; 72:205-212.
406. Wu JY, Shih JY, Chen KY, Yang CH, Yu CJ, Yang PC. Gefitinib therapy in patients with advanced non-small cell lung cancer with or without testing for epidermal growth factor receptor (EGFR) mutations. Medicine. 2011; 90:159-167.
407. Wu J-Y, Yu C-J, Chang Y-C, Yang C-H, Shih J-Y, Yang P-C. Effectiveness of Tyrosine Kinase Inhibitors on “Uncommon” Epidermal Growth Factor Receptor Mutations of Unknown Clinical Significance in Non–Small Cell Lung Cancer. Clinical Cancer Research. 2011; 17:3812-3821.
408. Xu C, Lin J, Wang Z, Zhou Q, Zhang G, Wang K, Yang X, Chen G, Yang J, Huang Y. [Relationship of the curative effects of gefitinib on non-small cell lung carcinoma to gender and to epithelial growth factor receptor status]. National Medical Journal of China. 2006; 86:2606-2610.
409. Xu M, Liu Y, Zhong H, Wu B. [Status and clinicopathologic implication of epidermal growth factor receptor mutation in non-small cell carcinoma of lung]. Chinese journal of pathology. 2007; 36:453-456.
410. Xu JM, Han Y, Duan HQ, Gao EM, Zhang Y, Liu XQ, Zhang JS, Toschi L, Galetta D, Azzariti A. EGFR mutations and HER2/3 protein expression and clinical outcome in Chinese advanced non-small cell lung cancer patients treated with gefitinib. Journal of cancer research and clinical oncology. 2009; 135:771-782.
411. Xu J, He J, Yang H, Luo X, Liang Z, Chen J, Cai Z, Xu J, Ren-Heidenreich L. Somatic mutation analysis of EGFR, KRAS, BRAF and PIK3CA in 861 patients with non-small cell lung cancer. Cancer biomarkers. 2011; 10:63-69.
412. Miyazawa H, Tanaka T, Nagai Y, Matsuoka M, Sutani A, Udagawa K, Zhang J, Hirama T, Murayama Y, Koyama N. Peptide nucleic acid–locked nucleic acid polymerase chain reaction clamp-based detection test for gefitinib-refractory T790M epidermal growth factor receptor mutation. Cancer science. 2008; 99:595-600.
413. Yamaguchi F, Kugawa S, Tateno H, Kokubu F, Fukuchi K. Analysis of EGFR, KRAS and P53 mutations in lung cancer using cells in the curette lavage fluid obtained by bronchoscopy. Lung cancer (Amsterdam, Netherlands). 2012; 78:201-206.

414. Yang SH, Yang P, Bowman ED, Meerzaman D, Mann F, Fukuoka J. Mutations in the Tyrosine Kinase Domain of the Epidermal Growth Factor Receptor in Non-Small Cell Lung Cancer. Proceedings of Conference: Tuberculosis and Respiratory Diseases. 2006; 103:51-51.
415. Yang C-H, Yu C-J, Shih J-Y, Chang Y-C, Hu F-C, Tsai M-C, Chen K-Y, Lin Z-Z, Huang C-J, Shun C-T. Specific EGFR mutations predict treatment outcome of stage IIIB/IV patients with chemotherapy-naive non–small-cell lung cancer receiving first-line gefitinib monotherapy. Journal of Clinical Oncology. 2008; 26:2745-2753.
416. Yang M-J, Hsu C-K, Chang H-J, Yen L-C, Tsao D-A, Chiu H-H, Huang Y-T, Chen Y-F, Wang J-Y, Lin S-R. The KRAS mutation is highly correlated with EGFR alterations in patients with non-small cell lung cancer. Fooyin Journal of Health Sciences. 2009; 1:65-71.
417. Yang L, Wang Z-j, An T-t, Bai H, Zhao J, Duan J-c, Li P-p, Wu M-n, Sun H, Liang L. A phase II trial of gefitinib as maintenance therapy after first-line chemotherapy for advanced non-small cell lung cancer in China. Chinese Journal of Cancer Research. 2010; 22:1-9.
418. Yang F, Chen K, Jiang G, Li J, Wang J. [Modified Restriction Enzyme-based Detection of EGFR Mutations in Lung Cancer]. Chinese journal of lung cancer. 2011; 14:637-641.
419. Yang SY, Yang TY, Chen KC, Li YJ, Hsu KH, Tsai CR, Chen CY, Hsu CP, Hsia JY, Chuang CY, Tsai YH, Chen KY, Huang MS, Su WC, Chen YM, Hsiung CA, et al. EGFR L858R mutation and polymorphisms of genes related to estrogen biosynthesis and metabolism in never-smoking female lung adenocarcinoma patients. Clinical cancer research: an official journal of the American Association for Cancer Research. 2011; 17:2149-2158.
420. Yang JC, Shih JY, Su WC, Hsia TC, Tsai CM, Ou SH, Yu CJ, Chang GC, Ho CL, Sequist LV, Dudek AZ, Shahidi M, Cong XJ, Lorence RM, Yang PC, Miller VA. Afatinib for patients with lung adenocarcinoma and epidermal growth factor receptor mutations (LUX-Lung 2): a phase 2 trial. The lancet oncology. 2012; 13:539-548.
421. Yin X, Jiang X, Yuan Y, Du Y. [Influence of mutations in epidermal growth factor receptor gene on growth, metastasis and survival rate of non-small cell lung carcinoma]. National Medical Journal of China. 2010; 90:1808-1812.
422. Yip PY, Yu B, Cooper WA, Selinger CI, Ng CC, Kennedy CW, Kohonen-Corish MR, McCaughan BC, Trent RJ, Boyer MJ, Kench JG, Horvath LG, O'Toole SA. Patterns of DNA mutations and ALK rearrangement in resected node negative lung adenocarcinoma. Journal of thoracic oncology: official publication of the International Association for the Study of Lung Cancer. 2013; 8:408-414.
423. Yoshida K, Yatabe Y, Park JY, Shimizu J, Horio Y, Matsuo K, Kosaka T, Mitsudomi T, Hida T. Prospective validation for prediction of gefitinib sensitivity by epidermal growth factor receptor gene mutation in patients with non-small cell lung cancer. Journal of thoracic oncology: official publication of the International Association for the Study of Lung Cancer. 2007; 2:22-28.
424. Yoshimasu T, Oura S, Ohta F, Hirai Y, Naito K, Nakamura R, Nishiguchi H, Hashimoto S, Kawago M, Okamura Y. Epidermal growth factor receptor mutations are associated with docetaxel sensitivity in lung cancer. Journal of thoracic oncology: official publication of the International Association for the Study of Lung Cancer. 2011; 6:1658-1662.
425. Yu Y, Song Z, Gao H, Zhu L, Lu S, Zhang J, Luo Q. EGFR L861Q mutation is a frequent feature of pulmonary mucoepidermoid carcinoma. Journal of cancer research and clinical oncology. 2012; 138:1421-1425.
426. Zhang XT, Li LY, Mu XL, Cui QC, Chang XY, Song W, Wang SL, Wang MZ, Zhong W, Zhang L. The EGFR mutation and its correlation with response of gefitinib in previously treated Chinese patients with advanced non-small-cell lung cancer. Annals of oncology: official journal of the European Society for Medical Oncology / ESMO. 2005; 16:1334-1342.
427. Zhang GC, Lin JY, Wang Z, Zhou Q, Xu CR, Zhu JQ, Wang K, Yang XN, Chen G, Yang JJ, Huang YJ, Liao RQ, Wu YL. Epidermal growth factor receptor double activating mutations involving both exons 19 and 21 exist in Chinese non-small cell lung cancer patients. Clinical oncology (Royal College of Radiologists (Great Britain)). 2007; 19:499-506.
428. Zhang J, Liang Z, Zeng X, Wu S, Gao J, Liu T. [Detection of epidermal growth factor receptor gene mutations in non-small cell lung cancers by real-time polymerase chain reaction using scorpion amplification refractory mutation system]. Chinese journal of pathology. 2008; 37:294-299.
429. Zhang X, Xu L, Wang H, Zhu Y, Liu Z, Yue W, Tang J, Wu W, Liu Z, Wu Y, Zhang C, Shi Y, Wang M, Shi H, Li M, Meng Q, et al. [The relationship between EGFR mutations and response and prognosis of tyrosine kinase inhibitors in advanced Non-small-cell Lung Cancer.]. Chinese journal of lung cancer. 2008; 11:206-213.
430. Zhang J, Wu J, Gao H, Zhu L, Shao J, Shi M, Han B. [Epidermal growth factor receptor gene mutations, amplification and clinicopathologic correlation in patients with lung cancer]. Chinese journal of pathology. 2011; 40:675-678.
431. Zhang L-J, Cai L, Li Z, Wang W-P, Guo K, Shao J-Y, Wang J-Y, Yu H, Rong T-H. Relationship between epidermal growth factor receptor gene mutation and copy number in Chinese patients with non-small cell lung cancer. Chinese journal of cancer. 2012; 31:491.
432. Zhao J, Wang Z-j, Duan J-c, Guo Q-z, Bai H, Yang L, An T-t, Wang X, Wang Y-y, Wu M-n. A phase II clinical trial of celecoxib combined with platinum-based regimen as

first-line chemotherapy for advanced non-small cell lung cancer patients with cyclooxygenase-2 positive expression. Chinese Journal of Cancer Research. 2009; 21:1-12.

433. Zhao J, Zhu Y, Zhang L, Yu Y, Zheng J. Detection of epidermal growth factor receptor gene mutations and its clinical significance in non-small cell lung cancers. Chin J Pathol. 2011; 40:671-674.
434. Zhao YL, Li Q, Li XH, Han WD, Hao HJ, Si YL, Wu ZQ. [Epidermal growth factor receptor mutation in non-small cell lung cancer and breast cancer]. Hereditas. 2007; 29:547-553.
435. Zhao X, Han R-B, Zhao J, Wang J, Yang F, Zhong W, Zhang L, Li L-Y, Wang M-Z. Comparison of epidermal growth factor receptor mutation statuses in tissue and plasma in stage I–IV non-small cell lung cancer patients. Respiration; international review of thoracic diseases. 2012; 85:119-125.
436. Zhong W-Z, Wu Y-L, Yang X-N, Guo A-L, Su J, Zhang X-C, Luo D-L, Wang Z, Chen H-J, Zhou Q. Genetic evolution of epidermal growth factor receptor in adenocarcinoma with a bronchioloalveolar carcinoma component. Clinical lung cancer. 2010; 11:160-168.
437. ZHONG W, WANG M, LI L, XIA Y, CHEN M, ZHANG L, ZHAO J. EGFR Gene Mutation Statuses in Advanced Non-small Cell Lung Cancer Patients and Their Influence on Effect of Gefitinib. Chinese Journal of Lung Cancer. 2012; 15.
438. Zhou C, Zhou S, Pan H, Su B, Gao Z. [Detection of epidermal growth factor receptor mutations in non-small cell lung cancer by real-time PCR using TaqMan-MGB probes]. Chinese journal of oncology. 2007; 29:119-123.
439. Zhou Q, Zhang X-C, Chen Z-H, Yin X-L, Yang J-J, Xu C-R, Yan H-H, Chen H-J, Su J, Zhong W-Z. Relative abundance of EGFR mutations predicts benefit from gefitinib treatment for advanced non–small-cell lung cancer. Journal of Clinical Oncology. 2011; 29:3316-3321.
440. Zhou C. (2012). Chemotherapy with target agents in advanced nonsmall cell lung cancer. International Association for the Study of Lung Cancer), pp. S439-S440.
441. Zhu C-Q, da Cunha Santos G, Ding K, Sakurada A, Cutz J-C, Liu N, Zhang T, Marrano P, Whitehead M, Squire JA. Role of KRAS and EGFR as biomarkers of response to erlotinib in National Cancer Institute of Canada Clinical Trials Group Study BR. 21. Journal of Clinical Oncology. 2008; 26:4268-4275.
442. Zhu Y-J, Xia Y, Ren G-j, Wang M-z, Zeng X, Zhang L. Efficacy and clinical/molecular predictors of erlotinib monotherapy for Chinese advanced non-small cell lung cancer. Chinese medical journal. 2010; 123:3200-3205.
443. Zhu J, Wang Y, Duan J, Bai H, Wang Z, Wei L, Zhao J, Zhuo M, Wang S, Yang L. DNA Methylation status of Wnt antagonist SFRP5 can predict the response to the EGFR-tyrosine kinase inhibitor therapy in non-small cell lung cancer. Journal of Experimental & Clinical Cancer Research. 2012; 31:1.
444. Zhuang Y-P, Wang H-Y, Shi M-Q, Zhang J, Feng Y. Use of CT-guided fine needle aspiration biopsy in epidermal growth factor receptor mutation analysis in patients with advanced lung cancer. Acta Radiologica. 2011; 52:1083-1087.
445. Zhuo M, Wu M, Zhao J, Song SW, Bai H, Wang S, Yang L, An T, Wang X, Duan J. Epidermal growth factor receptor genotype in plasma DNA and outcome of chemotherapy in the Chinese patients with advanced non-small cell lung cancer. Chinese medical journal. 2011; 124:3510-3514.
446. Zimmer S, Kahl P, Buhl TM, Steiner S, Wardelmann E, Merkelbach-Bruse S, Buettner R, Heukamp LC. Epidermal growth factor receptor mutations in non-small cell lung cancer influence downstream Akt, MAPK and Stat3 signaling. Journal of cancer research, clinical oncology. 2009; 135:723-730.
447. Girard N, Sima CS, Jackman DM, Sequist LV, Chen H, Yang JCH, Ji H, Waltman B, Rosell R, Taron M, Zakowski MF, Ladanyi M, Riely G, Pao W. Nomogram to predict the presence of EGFR activating mutation in lung adenocarcinoma. (Switzerland: European Respiratory Society (4 Ave Sainte-Luce, Lausanne CH-1003, Switzerland)), pp. 366-372.
448. Arrieta O, Cardona AF, Federico Bramuglia G, Gallo A, Campos-Parra AD, Serrano S, Castro M, Aviles A, Amorin E, Kirchuk R, Cuello M, Borbolla J, Riemersma O, Becerra H, Rosell R. Genotyping non-small cell lung cancer (NSCLC) in latin America. (United States: International Association for the Study of Lung Cancer (PO Box 6511, Mail Stop 8111, Aurora, Colorado 80045-0511, United States)), pp. 1955-1959.
449. Bai H, Wang Z, Chen K, Zhao J, Lee JJ, Wang S, Zhou Q, Zhuo M, Mao L, An T, Duan J, Yang L, Wu M, Liang Z, Wang Y, Kang X, et al. Influence of chemotherapy on EGFR mutation status among patients with non-small-cell lung cancer. (United States: American Society of Clinical Oncology (330 John Carlyle Street, Suite 300, Alexandria VA 22314, United States)), pp. 3077-3083.
450. Bell DW, Lynch TJ, Haserlat SM, Harris PL, Okimoto RA, Brannigan BW, Sgroi DC, Muir B, Riemenschneider MJ, Iacona RB, Krebs AD, Johnson DH, Giaccone G, Herbst RS, Manegold C, Fukuoka M, et al. (2005). Epidermal growth factor receptor mutations and gene amplification in non-small-cell lung cancer: Molecular analysis of the IDEAL/INTACT gefitinib trials. (United States: American Society of Clinical Oncology), pp. 8081-8092.
451. Kimura H, Fujiwara Y, Sone T, Kunitoh H, Tamura T, Kasahara K, Nishio K. EGFR mutation status in tumour-derived DNA from pleural effusion fluid is a practical basis for predicting the response to gefitinib. British journal of cancer. 2006; 95:1390-1395.
452. Leidner RS, Fu P, Clifford B, Hamdan A, Jin C, Eisenberg R, Boggon TJ, Skokan M, Franklin WA, Cappuzzo F, Hirsch FR, Varella-Garcia M, Halmos B. (2009). Genetic

abnormalities of the EGFR pathway in African American Patients with non-small-cell lung cancer. (United States, pp. 5620-5626.

453. Li C, Sun Y, Fang Z, Han X, Fang R, Zhang Y, Pan Y, Zhang W, Ren Y, Ji H. Comprehensive analysis of epidermal growth factor receptor gene status in lung adenocarcinoma. Journal of Thoracic Oncology. 6:1016-1021.
454. Mu XL, Li LY, Zhang XT, Wang MZ, Feng RE, Cui QC, Zhou HS, Guo BQ. (2005). Gefitinib-sensitive mutations of the epidermal growth factor receptor tyrosine kinase domain in Chinese patients with non-small cell lung cancer. (United States: American Association for Cancer Research Inc.), pp. 4289-4294.
455. Sasaki H, Endo K, Mizuno K, Yano M, Fukai I, Yamakawa Y, Fujii Y. EGFR mutation status and prognosis for gefitinib treatment in Japanese lung cancer. Lung cancer (Amsterdam, Netherlands). 2006; 51:135-136.
456. Yoshida K, Yatabe Y, Park J, Ogawa S, Park JY, Shimizu J, Horio Y, Matsuo K, Mitsudomi T, Hida T. Clinical outcomes of advanced non-small cell lung cancer patients screened for epidermal growth factor receptor gene mutations. Journal of cancer research and clinical oncology. 2010; 136:527-535.
