# Supplementary material for: The prevalence of EGFR mutation in patients with non-small cell lung cancer: a systematic review and meta-analysis
Source: Oncotarget. 2016 Oct 12;7(48):78985–93. doi: 10.18632/oncotarget.12587 (PMC5346692; doi:10.18632/oncotarget.12587)
Supplement: Supplementary file 2 [file oncotarget-07-78985-s002.docx]

Table A.1. The basic characteristics of included studies

| Study | Location | Design | Ethnicity | Sample-size | Proportion of females | Proportion of adenocarcinoma | Proportion of non-smokers |
| --- | --- | --- | --- | --- | --- | --- | --- |
| Addario 2008 | France | Case-series | Caucasian | 63 | 38.1% | 42.9% | 14.3% |
| Ahn 2012 | Korea | Cohort study | Asian | 92 | 38.0% | 47.5% | 31.7% |
| Ahn 2012 | Korea | Cohort study | Asian | 107 | 27.5% | NR | NR |
| Akamatsu 2012 | Japan | Cohort study | Asian | 44 | 27.3% | NR | NR |
| Akca 2012 | Turkey | Case-series | Caucasian | 52 | 25.0% | NR | NR |
| Akerley 2009 | U.S. | Clinical trial | Caucasian | 18 | 37.5% | 55.0% | 20.0% |
| Ambrosini-Spaltro 2011 | Italy | Case-series | Caucasian | 33 | 63.6% | NR | NR |
| An 2011 | China | Cohort study | Asian | 517 | NR | NR | NR |
| Andujar 2012 | France | Case-series | Caucasian | 100 | 12.0% | NR | NR |
| Angulo 2012 | Spain | Case-series | Caucasian | 133 | 39.1% | NR | NR |
| Arrieta 2011 | Peru | Case-series | Mixed | 203 | 48.8% | NR | NR |
| Arrieta 2011 | Argentina | Case-series | Caucasian | 244 | 57.4% | NR | NR |
| Arrieta 2011 | Colombia | Case-series | Mixed | 322 | 67.1% | NR | NR |
| Arrieta 2011 | Mexico | Case-series | Mixed | 381 | 56.7% | NR | NR |
| Arrieta 2011 | Latin America | Case-series | Mixed | 1150 | 58.4% | 87.8% | 52.4% |
| Asahina 2006 | Japan | Clinical trial | Asian | 82 | 59.8% | 87.8% | 46.3% |
| Azuma 2012 | Japan | Cohort study | Asian | 70 | NR | NR | NR |
| Bacchi 2012 | Brazil | Case-series | Mixed | 207 | 58.0% | NR | NR |
| Bae 2007 | Korea | Case-series | Asian | 115 | 24.3% | 47.8% | 20.0% |
| Bai 2009 | China | Case-series | Asian | 230 | 46.5% | 74.3% | 55.2% |
| Bai 2012 | China | Cohort study | Asian | 63 | 25.4% | 39.7% | 47.6% |
| Bai 2012 | China | Cohort study | Asian | 79 | 44.3% | 73.4% | NR |
| Bai 2012 | China | Cohort study | Asian | 264 | 45.5% | 77.7% | 48.5% |
| Baykara 2012 | Turkey | Case-series | Caucasian | 50 | 12.0% | NR | NR |
| Bell 2005 | U.S. | Clinical trial | Mixed | 79 | 36.7% | 11.5% | 5.6% |
| Bell 2005 | U.S. | Clinical trial | Mixed | 312 | 30.4% | 7.7% | NR |
| Bichev 2012 | Bulgaria | Case-series | Caucasian | 709 | 29.3% | NR | NR |
| Billah 2011 | U.S. | Case-series | Mixed | 175 | 60.3% | 51.2% | NR |
| Bloom 2012 | U.S. | Case-series | Unclear | 18246 | NR | NR | NR |
| Boch 2013 | Germany | Case-series | Caucasian | 552 | 36.2% | NR | NR |
| Boldrini 2007 | Italy | Case-series | Caucasian | 23 | 39.1% | NR | 21.7% |
| Bonanno 2010 | Italy | Cohort study | Caucasian | 60 | 50.0% | NR | NR |
| Bosdet 2012 | Canada | Case-series | Unclear | 509 | NR | NR | NR |
| Brachtel 2006 | U.S. | Case-series | African American | 37 | 56.8% | NR | NR |
| Brevet 2010 | U.S. | Case-series | Unclear | 31 | 51.6% | NR | NR |
| Buckingham 2007 | U.S. | Cohort study | Unclear | 58 | 54.3% | NR | 14.6% |
| Cadranel 2012 | France | Cohort study | Caucasian | 307 | 32.6% | NR | NR |
| Camidge 2011 | U.S. | Cohort study | Unclear | 89 | 65.2% | 93.3% | 48.3% |
| Cappuzzo 2005 | Italy | Clinical trial | Caucasian | 89 | 34.3% | 52.9% | 14.7% |
| Cappuzzo 2007 | Italy | Case-series | Caucasian | 65 | 32.6% | 48.9% | 15.8% |
| Cardona 2011 | Colombia | Case-series | Unclear | 228 | 33.3% | 62.3% | NR |
| Castro 2012 | Portugal | Cohort study | Caucasian | 374 | 28.9% | NR | NR |
| Chamberlin 2011 | UK | Case-series | Caucasian | 314 | 48.7% | NR | NR |
| Chang 2007 | Taiwan | Cohort study | Asian | 58 | 39.7% | NR | NR |
| Chang 2010 | Taiwan | Case-series | Asian | 42 | 35.7% | NR | NR |
| Chang 2011 | Taiwan | Case-series | Asian | 56 | 37.5% | 60.7% | 41.1% |
| Chen 2008 | Taiwan | Case-series | Asian | 17 | 47.1% | 82.4% | NR |
| Chen 2008 | Taiwan | Cohort study | Asian | 20 | 58.9% | 98.2% | 75.0% |
| Chen 2011 | China | Case-series | Asian | 40 | 30.0% | 87.5% | NR |
| Chen 2011 | China | Case-series | Asian | 61 | 60.7% | 62.3% | 68.9% |
| Chen 2011 | Taiwan | Cohort study | Asian | 207 | 38.5% | 66.8% | 58.7% |
| Chen 2012 | Taiwan | Clinical trial | Asian | 60 | 17.5% | 64.6% | 78.8% |
| Cho 2007 | Korea | Clinical trial | Asian | 17 | 52.4% | 71.4% | 52.4% |
| Choi 2010 | Korea | Case-series | Asian | 98 | 100.0% | 82.7% | 83.7% |
| Choi 2012 | U.S. | Cohort study | Caucasian | 96 | NR | NR | NR |
| Choi 2012 | Korea | Case-series | Asian | 163 | 39.3% | NR | NR |
| Choi 2012 | Korea | Case-series | Asian | 331 | 52.3% | NR | NR |
| Choi 2012 | Korea | Case-series | Asian | 581 | NR | NR | NR |
| Chung 2010 | Taiwan | Clinical trial | Asian | 388 | 57.7% | NR | NR |
| Chung 2011 | Taiwan | Cohort study | Asian | 59 | 47.7% | 80.4% | 34.6% |
| Ck 2011 | Malaysia | Cohort study | Asian | 72 | 50.0% | 100.0% | 58.3% |
| Conde 2005 | Spain | Case-series | Caucasian | 86 | 34.9% | NR | NR |
| Cortes-Funes 2005 | Spain | Case-series | Caucasian | 83 | 30.1% | 50.6% | 24.1% |
| Cote 2011 | U.S. | Case-series | Mixed | 144 | 70.8% | 72.9% | 22.2% |
| Dacic 2009 | U.S. | Case-series | Unclear | 345 | 57.4% | NR | NR |
| D'Angelo 2010 | U.S. | Case-series | Unclear | 2142 | 61.4% | NR | NR |
| Daniele 2009 | Italy | Case-series | Caucasian | 35 | 11.6% | 47.4% | NR |
| Dasgupta 2011 | U.S. | Case-series | Mixed | 60 | 76.7% | NR | NR |
| David 2005 | U.S. | Clinical trial | Unclear | 228 | 43.9% | 46.1% | 8.8% |
| de Mello 2012 | Portugal | Cohort study | Caucasian | 248 | 33.3% | NR | NR |
| De Pas 2011 | Italy | Cohort study | Caucasian | 681 | NR | NR | NR |
| Deng 2009 | China | Case-series | Asian | 62 | 35.5% | 61.3% | 53.2% |
| Dingemans 2011 | Netherlands | Clinical trial | Caucasian | 27 | 51.1% | 66.0% | 17.0% |
| Doebele 2012 | U.S. | Cohort study | Unclear | 209 | 60.3% | 95.7% | 40.2% |
| Dogan 2012 | U.S. | Case-series | Mixed | 3026 | 62.7% | NR | NR |
| Dong 2006 | China | Case-series | Asian | 176 | 26.1% | NR | NR |
| Dongiovannia 2008 | Italy | Case-series | Caucasian | 43 | 35.8% | 32.1% | NR |
| Eberhardt 2011 | Germany | Cohort study | Caucasian | 3155 | NR | NR | NR |
| Ebi 2008 | Japan | Clinical trial | Asian | 17 | 65.3% | 81.6% | 61.2% |
| Eichler 2010 | U.S. | Cohort study | Caucasian | 93 | 66.7% | 93.5% | 43.0% |
| El-Zammar 2009 | U.S. | Case-series | African American | 49 | 59.2% | NR | NR |
| Enting 2011 | UK | Cohort study | Caucasian | 88 | NR | 100.0% | NR |
| Fan 2011 | China | Cohort study | Asian | 282 | 34.0% | NR | NR |
| Fang 2011 | China | Case-series | Asian | 35 | NR | NR | NR |
| Feng 2011 | China | Case-series | Asian | 309 | 40.5% | NR | NR |
| Fiala 2012 | Czech Republic | Case-series | Caucasian | 613 | NR | NR | NR |
| Fong 2010 | Taiwan | Case-series | Asian | 77 | 54.5% | NR | NR |
| Fujimoto 2012 | Japan | Case-series | Asian | 555 | 44.5% | NR | NR |
| Fujita 2012 | Japan | Clinical trial | Asian | 54 | 63.0% | 68.5% | 53.7% |
| Fujiwara 2006 | Japan | Cohort study | Asian | 26 | 42.3% | 84.6% | 42.3% |
| Fukuoka 2011 | Japan | Clinical trial | Asian | 437 | 76.7% | 35.9% | 33.3% |
| Gandara 2010 | U.S. | Case-series | Unclear | 1207 | 55.2% | NR | NR |
| Gao 2010 | China | Case-series | Asian | 86 | 43.0% | NR | NR |
| Gao 2012 | China | Cohort study | Asian | 120 | 35.8% | NR | NR |
| Garcia-Olive 2009 | Spain | Case-series | Caucasian | 51 | 19.6% | NR | NR |
| Giovannetti 2010 | Italy | Cohort study | Caucasian | 62 | 42.7% | 56.3% | 30.2% |
| Girard 2010 | Korea | Case-series | Asian | 100 | 32.0% | 53.0% | 43.0% |
| Girard 2010 | U.S. | Case-control study | Mixed | 369 | 87.8% | 99.5% | NR |
| Girard 2012 | U.S. | Case-series | Asian | 364 | 58.2% | NR | 70.7% |
| Girard 2012 | U.S. | Case-series | Caucasian | 2392 | 64.4% | 83.9% | 24.6% |
| Goto 2011 | Japan | Clinical trial | Asian | 91 | 87.6% | NR | NR |
| Gow 2009 | Taiwan | Case-series | Asian | 67 | 40.3% | 62.7% | 61.2% |
| Guo 2007 | China | Case-series | Asian | 63 | 46.0% | 73.0% | 57.1% |
| Guo 2010 | China | Cohort study | Asian | 88 | 45.5% | 78.4% | 58.0% |
| Guo 2011 | China | Cohort study | Asian | 128 | 46.9% | NR | NR |
| Gwak 2005 | Korea | Case-series | Asian | 22 | 45.5% | NR | NR |
| Han 2005 | Korea | Cohort study | Asian | 90 | 40.0% | 61.1% | 47.8% |
| Han 2007 | China | Cohort study | Asian | 106 | 48.1% | 73.6% | 67.0% |
| Han 2011 | China | Case-series | Asian | 33 | 39.4% | NR | NR |
| Han 2011 | Korea | Case-series | Asian | 37 | 45.9% | NR | 48.6% |
| Han 2012 | Korea | Case-series | Asian | 41 | 31.7% | NR | 31.7% |
| Hana 2006 | Korea | Case-series | Asian | 86 | 43.0% | 59.3% | 44.2% |
| Hana 2006 | Korea | Cohort study | Asian | 120 | 41.7% | 59.2% | 44.2% |
| Hana 2011 | Korea | Clinical trial | Asian | 90 | 46.7% | 76.7% | 47.8% |
| Haneda 2005 | Japan | Cohort study | Asian | 112 | 37.5% | NR | NR |
| Harada 2011 | U.S. | Case-series | African American | 16 | 56.3% | 75.0% | 43.8% |
| Hata 2010 | Japan | Case-series | Asian | 44 | 68.2% | NR | 63.6% |
| Hata 2010 | Japan | Case-series | Asian | 783 | NR | NR | NR |
| Hata 2013 | Japan | Cohort study | Asian | 249 | NR | NR | NR |
| He 2009 | China | Case-series | Asian | 134 | 36.6% | 75.4% | 53.0% |
| He 2013 | China | Case-series | Asian | 538 | 44.2% | NR | NR |
| Helland 2011 | Norway | Cohort study | Caucasian | 240 | 48.3% | NR | NR |
| Hirsch 2007 | Italy and U.S. | Cohort study | Unclear | 155 | 43.1% | 48.5% | 20.6% |
| Hlinkova 2013 | Slovakia | Case-series | Caucasian | 835 | 26.2% | NR | NR |
| Horiike 2006 | Japan | Case-series | Asian | 94 | 33.0% | NR | NR |
| Hosokawa 2009 | Japan | Case-series | Asian | 93 | 33.3% | 73.1% | 37.6% |
| Hotta 2007 | Japan | Cohort study | Asian | 60 | 41.7% | 73.3% | 36.7% |
| Hou 2013 | China | Case-series | Asian | 40 | 27.5% | NR | NR |
| Hsieh 2004 | Taiwan | Clinical trial | Asian | 35 | 51.4% | 40.0% | 60.0% |
| Hsieh 2006 | Taiwan | Cohort study | Asian | 65 | 56.9% | 80.0% | 60.0% |
| Hsu 2011 | Taiwan | Case-series | Asian | 162 | 55.6% | NR | NR |
| Hsu 2012 | Taiwan | Case-series | Asian | 58 | 51.4% | 82.6% | 56.9% |
| Huang 2004 | Taiwan | Case-series | Asian | 101 | 44.6% | 68.3% | NR |
| Huang 2007 | China | Case-series | Asian | 116 | 34.5% | 47.4% | NR |
| Huang 2010 | Taiwan | Cohort study | Asian | 77 | 57.1% | NR | NR |
| Huang 2011 | China | Case-series | Asian | 1195 | 33.9% | 71.8% | 43.0% |
| Huang 2012 | China | Cohort study | Asian | 207 | 53.6% | NR | NR |
| Hung 2006 | Taiwan | Case-series | Asian | 29 | 51.7% | 89.7% | 72.4% |
| Inoue 2006 | Japan | Clinical trial | Asian | 75 | 60.0% | 85.3% | 46.7% |
| Jackman 2007 | U.S. | Clinical trial | Caucasian | 43 | 50.0% | 60.0% | 10.0% |
| Jackman 2009 | U.S. | Clinical trial | Caucasian | 223 | 68.6% | 85.7% | 32.7% |
| Jang 2009 | Korea | Case-series | Asian | 104 | 46.2% | NR | NR |
| Janne 2012 | U.S. | Clinical trial | Mixed | 164 | 59.1% | 85.6% | 79.0% |
| Jia 2011 | China | Case-series | Asian | 55 | 41.8% | 100.0% | 76.4% |
| Jiang 2011 | China | Case-series | Asian | 33 | 27.3% | NR | NR |
| Jida 2009 | Japan | Cohort study | Asian | 1001 | 36.5% | 77.8% | 37.5% |
| Jin 2010 | Korea | Case-series | Asian | 176 | 34.7% | 66.5% | 32.4% |
| Johnson 2012 | U.S. | Cohort study | Unclear | 1036 | 41.1% | NR | NR |
| Joseph 2010 | U.S. | Clinical trial | Caucasian | 41 | 57.8% | 64.7% | NR |
| Jung 2011 | Korea | Cohort study | Asian | 84 | 43.1% | 59.3% | 48.0% |
| Kaira 2006 | U.S. | Cohort study | Caucasian | 26 | 80.8% | NR | NR |
| Kaira 2010 | Japan | Cohort study | Asian | 17 | 23.5% | NR | NR |
| Kalikaki 2008 | Greece | Case-series | Caucasian | 25 | 12.0% | NR | NR |
| Kalikaki 2010 | Greece | Cohort study | Caucasian | 162 | 22.2% | NR | 28.4% |
| Kanaji 2011 | Japan | Case-series | Asian | 70 | NR | NR | NR |
| Kanaji 2012 | Japan | Case-series | Asian | 161 | NR | NR | NR |
| Kang 2007 | Korea | Case-series | Asian | 25 | 57.1% | NR | 52.0% |
| Kato 2010 | Japan | Case-series | Asian | 70 | 48.6% | 81.4% | 41.4% |
| Kato 2012 | Japan | Case-series | Asian | 42 | 33.3% | NR | NR |
| Katsuhiko 2005 | Japan | Case-series | Asian | 182 | 30.2% | NR | NR |
| Kawada 2008 | Japan | Case-series | Asian | 109 | 45.9% | NR | NR |
| Kawaguchi 2010 | Japan | Cohort study | Asian | 126 | 88.1% | 96.8% | NR |
| Kawahara 2010 | Japan | Cohort study | Asian | 170 | 35.9% | NR | NR |
| Kawano 2011 | Japan | Cohort study | Asian | 30 | 60.0% | NR | 50.0% |
| Kayatani 2011 | Japan | Cohort study | Asian | 41 | NR | NR | NR |
| Kim 2005 | Korea | Cohort study | Asian | 27 | 22.4% | 41.8% | 17.3% |
| Kim 2007 | Korea | Case-series | Asian | 71 | 59.2% | NR | NR |
| Kim 2008 | Korea | Cohort study | Asian | 144 | 26.4% | 41.0% | 31.9% |
| Kim 2011 | Korea | Cohort study | Asian | 50 | 28.0% | NR | NR |
| Kim 2011 | Korea | Case-series | Asian | 112 | NR | NR | NR |
| Kim 2011 | Korea | Case-series | Asian | 205 | 43.4% | NR | NR |
| Kim 2012 | Japan | Clinical trial | Asian | 44 | 40.8% | 98.0% | 49.0% |
| Kim 2012 | Korea | Case-series | Asian | 202 | 35.1% | NR | NR |
| Kim 2012 | Korea | Cohort study | Asian | 229 | 86.9% | 93.9% | 100.0% |
| Kim 2012 | Korea | Case-series | Asian | 240 | 41.1% | 73.3% | 49.6% |
| Kim 2012 | Korea | Cohort study | Asian | 863 | 39.7% | NR | NR |
| Kim 2013 | Korea | Cohort study | Asian | 57 | 38.6% | NR | NR |
| Kimura 2006 | Japan | Case-series | Asian | 24 | 45.8% | NR | NR |
| Kimura 2006 | Japan | Case-series | Asian | 27 | 37.0% | 85.2% | NR |
| Kimura 2006 | Japan | Case-series | Asian | 43 | 48.8% | NR | NR |
| Koga 2010 | Japan | Cohort study | Asian | 208 | 40.4% | 79.3% | 39.4% |
| Kosaka 2004 | Japan | Cohort study | Asian | 277 | 42.6% | NR | NR |
| Kosaka 2008 | Japan | Cohort study | Asian | 397 | 49.4% | NR | NR |
| Koudelakova 2012 | Czech Republic | Case-series | Caucasian | 185 | 34.1% | 47.6% | NR |
| Koyama 2006 | Japan | Cohort study | Asian | 44 | 59.1% | 25.3% | 18.8% |
| Lai 2006 | China | Case-series | Asian | 32 | 21.9% | 59.4% | NR |
| Leary 2012 | UK | Cohort study | Mixed | 119 | NR | NR | NR |
| Lecia 2010 | U.S. | Clinical trial | Mixed | 68 | 61.8% | 71.1% | 39.5% |
| Lee 2006 | Korea | Cohort study | Asian | 90 | 40.0% | 16.0% | NR |
| Lee 2008 | Korea | Cohort study | Asian | 130 | 25.4% | 39.2% | 30.8% |
| Lee 2009 | Korea | Cohort study | Asian | 117 | 61.5% | NR | NR |
| Lee 2010 | Korea | Cohort study | Asian | 69 | 53.6% | NR | NR |
| Lee 2010 | Korea | Case-series | Asian | 173 | 34.7% | NR | NR |
| Lee 2010 | Korea | Cohort study | Asian | 179 | 87.2% | 86.6% | 24.6% |
| Lee 2010 | Korea | Cohort study | Asian | 324 | 45.1% | 71.9% | 52.8% |
| Lee 2011 | Korea | Cohort study | Asian | 95 | 53.7% | 82.1% | 62.1% |
| Lee 2011 | Korea | Case-series | Asian | 153 | 52.3% | NR | NR |
| Lee 2012 | Taiwan | Cohort study | Asian | 43 | 46.5% | NR | NR |
| Lee 2013 | Korea | Cohort study | Asian | 61 | 57.4% | NR | NR |
| Leidner 2009 | U.S. | Case-series | African American | 53 | 52.8% | 34.0% | 13.2% |
| Leidner 2009 | U.S. | Case-series | Caucasian | 89 | 34.3% | 25.5% | 14.7% |
| Leidner 2011 | Singapore | Case-series | Asian | 90 | 45.6% | 100.0% | 41.1% |
| Leidner 2011 | France | Case-series | Caucasian | 136 | 26.5% | 100.0% | 9.6% |
| Li 2008 | China | Case-series | Asian | 46 | 32.6% | 50.0% | 43.5% |
| Li 2008 | U.S. | Case-series | Caucasian | 60 | 76.7% | NR | NR |
| Li 2011 | China | Case-series | Asian | 89 | 44.9% | 100.0% | 56.2% |
| Li 2011 | China | Cohort study | Asian | 99 | 55.6% | 85.9% | 70.7% |
| Li 2011 | China | Case-series | Asian | 118 | 37.3% | 81.4% | 49.2% |
| Li 2011 | China | Case-series | Asian | 157 | 42.7% | NR | NR |
| Li 2011 | China | Cohort study | Asian | 187 | 48.7% | NR | 58.3% |
| Li 2011 | China | Case-series | Asian | 202 | 78.7% | NR | NR |
| Li 2012 | China | Case-series | Asian | 230 | 3.0% | NR | NR |
| Liang 2008 | China | Case-series | Asian | 290 | 50.0% | 84.1% | NR |
| Liang 2010 | China | Case-series | Asian | 133 | 53.4% | NR | NR |
| Lim 2009 | Singapore | Cohort study | Asian | 88 | 25.0% | 43.8% | 26.0% |
| Lin 2010 | Asian countries | Clinical trial | Asian | 122 | 38.5% | 79.5% | 50.0% |
| Lind 2010 | Netherlands | Clinical trial | Caucasian | 36 | 44.0% | 72.0% | 22.0% |
| Liu 2007 | China | Case-series | Asian | 101 | 28.7% | 51.5% | 34.7% |
| Liu 2008 | China | Case-series | Asian | 60 | 63.3% | NR | NR |
| Liu 2009 | China | Cohort study | Asian | 187 | 33.2% | 40.6% | 36.4% |
| Liu 2010 | Taiwan | Cohort study | Asian | 164 | 32.9% | 59.1% | 58.5% |
| Liu 2011 | China | Case-series | Asian | 220 | NR | NR | NR |
| Liu 2012 | China | Case-series | Asian | 212 | 48.1% | NR | NR |
| Liu 2012 | China | Cohort study | Asian | 383 | 43.1% | NR | NR |
| Loprevite 2007 | Italy | Cohort study | Caucasian | 21 | 29.1% | 74.1% | 19.0% |
| Lozano 2011 | Spain | Cohort study | Caucasian | 150 | 41.3% | 73.3% | 26.7% |
| Lu 2011 | China | Case-series | Asian | 30 | 26.7% | NR | NR |
| Ludovini 2011 | Italy | Cohort study | Caucasian | 166 | 45.2% | 61.4% | 36.7% |
| Lv 2013 | China | Case-series | Asian | 11 | 54.5% | NR | NR |
| Mack 2009 | U.S. | Cohort study | Caucasian | 48 | 56.3% | NR | NR |
| Mak 2011 | U.S. | Cohort study | Caucasian | 123 | 65.0% | NR | NR |
| Malapelle 2011 | Italy | Case-series | Caucasian | 42 | 69.0% | 78.6% | 9.5% |
| Marchetti 2005 | Italy | Case-series | Caucasian | 860 | 13.0% | 33.6% | 54.3% |
| Marks 2008 | U.S. | Cohort study | Caucasian | 296 | 59.5% | NR | NR |
| Martinez 2012 | U.S. | Case-series | Caucasian | 99 | 48.5% | NR | NR |
| Martnez-Navarro 2011 | Spain | Case-series | Caucasian | 69 | 24.6% | 78.3% | 24.6% |
| Masago 2008 | Japan | Cohort study | Asian | 46 | NR | NR | NR |
| Masago 2011 | Japan | Cohort study | Asian | 80 | 60.0% | 95.0% | 61.3% |
| Massarelli 2006 | U.S. | Case-series | Mixed | 71 | 57.7% | NR | NR |
| Matsumoto 2005 | Japan | Case-series | Asian | 19 | 31.6% | NR | NR |
| McMillen 2010 | China | Case-series | Asian | 218 | 36.2% | NR | NR |
| Milella 2012 | Italy | Clinical trial | Caucasian | 112 | 40.4% | 66.5% | 31.9% |
| Miller 2008 | U.S. | Clinical trial | Unclear | 81 | 65.3% | 88.1% | NR |
| Mitsudomi 2004 | Japan | Cohort study | Asian | 59 | 45.8% | 84.7% | 47.5% |
| Miyamae 2010 | Japan | Case-series | Asian | 89 | 6.7% | NR | NR |
| Mok 2009 | China | Clinical trial | Asian | 437 | 79.3% | 96.3% | 93.7% |
| Molina-Vila 2008 | Spain | Case-series | Caucasian | 217 | 46.7% | 83.5% | 49.5% |
| Morinaga 2008 | Japan | Cohort study | Asian | 100 | 36.0% | NR | NR |
| Mu 2005 | China | Case-series | Asian | 22 | 54.5% | NR | NR |
| Mu 2005 | China | Case-series | Asian | 54 | 42.6% | NR | NR |
| Na 2007 | Korea | Cohort study | Asian | 133 | 52.6% | NR | 51.9% |
| Na 2012 | Korea | Cohort study | Asian | 86 | 100.0% | NR | NR |
| Nagashima 2012 | Japan | Cohort study | Asian | 12 | 41.7% | NR | NR |
| Nakajima 2007 | Japan | Case-series | Asian | 43 | 30.2% | NR | NR |
| Nakajima 2011 | Japan | Case-series | Asian | 156 | 26.3% | 81.4% | 25.0% |
| Nakatani 2006 | Japan | Case-series | Asian | 30 | 40.0% | NR | NR |
| Nasioulas 2011 | Greece | Case-series | Caucasian | 342 | NR | NR | NR |
| Neal 2010 | U.S. | Clinical trial | Mixed | 18 | 77.8% | 83.3% | 27.8% |
| Niho 2006 | Japan | Clinical trial | Asian | 13 | 40.0% | 75.0% | 20.0% |
| Ninomiya 2009 | Japan | Case-series | Asian | 107 | 51.4% | NR | NR |
| Nong 2013 | China | Cohort study | Asian | 32 | 62.5% | NR | NR |
| Noronha 2013 | U.S. | Cohort study | Caucasian | 111 | 47.7% | NR | NR |
| Nose 2008 | Japan | Cohort study | Asian | 447 | 41.0% | NR | NR |
| Ohtsuka 2007 | Japan | Case-series | Asian | 42 | 9.5% | NR | 9.5% |
| Okada 2012 | Japan | Case-series | Asian | 14 | 35.7% | NR | NR |
| Okudela 2008 | Japan | Case-series | Asian | 153 | 49.0% | NR | NR |
| Onitsuka 2010 | Japan | Case-series | Asian | 183 | 44.3% | NR | NR |
| Oshita 2010 | Japan | Cohort study | Asian | 146 | 49.3% | 83.6% | 43.8% |
| Otani 2008 | Japan | Case-series | Asian | 53 | 41.5% | 56.6% | 39.6% |
| Pai 2011 | India | Case-series | Asian | 46 | 28.3% | NR | NR |
| Paik 2010 | U.S. | Cohort study | Caucasian | 687 | 65.8% | NR | NR |
| Paik 2012 | U.S. | Cohort study | Caucasian | 675 | NR | NR | NR |
| Pallis 2012 | Greece | Clinical trial | Caucasian | 36 | 65.3% | 93.9% | 100.0% |
| Pan 2005 | China | Case-series | Asian | 52 | 25.0% | 44.2% | NR |
| Park 2009 | Korea | Case-series | Asian | 101 | 27.7% | NR | NR |
| Park 2012 | Korea | Cohort study | Asian | 217 | 55.3% | NR | NR |
| Peng 2010 | China | Case-series | Asian | 96 | 30.2% | NR | NR |
| Pennycuick 2012 | UK | Case-series | Mixed | 215 | 57.4% | NR | 4.1% |
| Pham 2005 | U.S. | Case-series | Caucasian | 265 | 66.4% | NR | NR |
| Pietanza 2012 | U.S. | Clinical trial | Caucasian | 38 | 76.4% | NR | 52.7% |
| Pietanza 2012 | U.S. | Clinical trial | Caucasian | 40 | 75.6% | 90.2% | 73.2% |
| Porta 2011 | Spain | Cohort study | Caucasian | 69 | 46.4% | 68.1% | 31.9% |
| Price 2010 | U.S. | Clinical trial | Unclear | 59 | 50.0% | 85.5% | 0.0% |
| Provencio 2011 | Spain | Case-series | Caucasian | 2105 | 38.7% | NR | NR |
| Pugh 2007 | Canada | Cohort study | Mixed | 38 | 60.5% | NR | NR |
| Raez 2011 | U.S. | Cohort study | Unclear | 32 | NR | NR | NR |
| RagU.S. 2013 | Italy | Cohort study | Caucasian | 230 | 21.7% | NR | NR |
| Rahman 2012 | Japan | Case-series | Asian | 61 | 23.0% | NR | NR |
| Ready 2010 | U.S. | Clinical trial | Caucasian | 45 | 26.7% | 33.3% | 10.0% |
| Reinersman 2011 | U.S. | Case-series | African American | 121 | NR | NR | NR |
| Reinmuth 2008 | Germany | Case-series | Caucasian | 120 | 38.3% | NR | NR |
| Rekhtman 2011 | U.S. | Case-series | Unclear | 126 | 64.1% | NR | NR |
| Rekhtman 2012 | U.S. | Case-series | Caucasian | 180 | 59.4% | NR | NR |
| Ren 2011 | China | Case-series | Asian | 14 | 57.1% | 78.6% | NR |
| Ren 2012 | China | Case-series | Asian | 181 | 38.7% | NR | NR |
| Riely 2006 | U.S. | Cohort study | Caucasian | 291 | 66.7% | NR | NR |
| Righi 2013 | Italy | Case-series | Caucasian | 321 | 42.1% | NR | NR |
| Rosell 2009 | Spain | Cohort study | Caucasian | 2105 | 38.7% | 81.1% | 30.4% |
| Sahoo 2011 | India | Case-series | Asian | 220 | 44.1% | 80.0% | 52.7% |
| Saieg 2012 | Canada | Case-series | Caucasian | 210 | NR | NR | NR |
| Sakuma 2007 | Japan | Case-series | Asian | 141 | 51.1% | NR | NR |
| Sartori 2009 | Italy | Case-series | Caucasian | 418 | 47.6% | NR | NR |
| Sasaki 2005 | Japan | Case-series | Asian | 95 | 31.6% | NR | NR |
| Sasaki 2005 | Japan | Case-series | Asian | 102 | 20.6% | NR | NR |
| Sasaki 2006 | Japan | Case-series | Asian | 252 | 25.4% | NR | NR |
| Sasaki 2006 | Japan | Case-series | Asian | 575 | 32.0% | 63.0% | 34.1% |
| Sasaki 2007 | Japan | Case-series | Asian | 27 | 48.1% | 81.5% | 55.6% |
| Sasaki 2007 | Japan | Case-series | Asian | 303 | 31.0% | 64.0% | 32.3% |
| Sasaki 2007 | Japan | Case-series | Asian | 322 | 27.3% | NR | NR |
| Sasaki 2008 | Japan | Cohort study | Asian | 398 | 32.2% | 67.3% | 34.7% |
| Sasaki 2009 | Japan | Case-series | Asian | 109 | 40.4% | 83.5% | 45.9% |
| Sasaki 2011 | Japan | Case-series | Asian | 283 | 33.2% | 66.8% | 33.9% |
| Savic 2008 | U.S. | Case-series | Unclear | 80 | 31.3% | 58.3% | NR |
| Schmid 2009 | AU.S.tria | Case-series | Caucasian | 96 | 39.6% | NR | NR |
| Schneider 2008 | Germany | Case-series | Caucasian | 92 | 41.0% | 50.9% | 24.4% |
| Scoccianti 2011 | France | Cohort study | Caucasian | 130 | 20.8% | NR | NR |
| Sekine 2011 | Japan | Case-series | Asian | 57 | 49.1% | NR | NR |
| Sequist 2008 | U.S. | Cohort study | Mixed | 98 | 70.4% | 90.8% | 37.8% |
| Sequist 2010 | U.S. | Case-series | Mixed | 76 | 63.2% | NR | NR |
| Sequist 2010 | U.S. | Clinical trial | Mixed | 139 | 71.9% | 90.6% | 38.8% |
| Sequist 2011 | U.S. | Case-series | Caucasian | 526 | 58.2% | 80.6% | 23.4% |
| Shaw 2009 | Australia, U.S. and Israel | Cohort study | Mixed | 141 | 66.0% | NR | NR |
| Shi 2011 | China | Cohort study | Asian | 40 | 72.5% | 100.0% | NR |
| Shiau 2012 | Canada | Case-series | Unclear | 1677 | NR | NR | NR |
| Shigematsu 2005 | U.S. | Cohort study | Caucasian | 80 | 46.3% | NR | NR |
| Shigematsu 2005 | Australia | Cohort study | Caucasian | 83 | 30.1% | NR | NR |
| Shigematsu 2005 | Taiwan | Cohort study | Asian | 93 | 31.2% | NR | NR |
| Shigematsu 2005 | Japan | Cohort study | Asian | 263 | 30.4% | NR | NR |
| Shih 2006 | Taiwan | Cohort study | Asian | 62 | 61.3% | 91.9% | 66.1% |
| Shingyoji 2011 | Japan | Case-series | Asian | 20 | 50.0% | NR | NR |
| Shiozawa 2012 | Japan | Cohort study | Asian | 59 | 25.4% | NR | NR |
| Skov 2012 | Denmark | Case-series | Caucasian | 401 | 47.4% | NR | NR |
| Smits 2012 | Netherlands | Case-series | Caucasian | 778 | 45.9% | 79.7% | 3.7% |
| SmoU.S.e 2009 | U.S. | Case-series | Unclear | 236 | NR | NR | NR |
| Sone 2007 | Japan | Cohort study | Asian | 59 | 40.7% | 74.6% | 35.6% |
| Song 2008 | China | Case-series | Asian | 50 | 46.0% | 48.0% | 50.0% |
| Song 2011 | China | Case-series | Asian | 15 | 55.0% | 90.0% | 75.0% |
| Song 2013 | China | Cohort study | Asian | 49 | 46.9% | NR | NR |
| Sonobe 2005 | Japan | Case-series | Asian | 108 | 50.9% | 100.0% | 49.1% |
| Sonobe 2006 | Japan | Cohort study | Asian | 53 | 32.1% | 73.6% | 26.4% |
| Sonobe 2011 | Japan | Cohort study | Asian | 180 | 61.7% | NR | NR |
| Soung 2005 | Korea | Case-series | Asian | 153 | 27.5% | NR | NR |
| Spigel 2011 | U.S. | Clinical trial | Caucasian | 72 | 47.0% | NR | NR |
| Sriram 2010 | Australia | Case-series | Mixed | 522 | 33.9% | NR | NR |
| Sriuranpong 2005 | Thailand | Case-series | Asian | 60 | 38.3% | NR | NR |
| Suehisa 2007 | Japan | Cohort study | Asian | 187 | 47.6% | NR | NR |
| Sueoka 2006 | Japan | Case-series | Asian | 97 | 39.2% | NR | NR |
| Sugano 2011 | Japan | Case-series | Asian | 136 | 55.9% | NR | NR |
| Sugio 2005 | Japan | Cohort study | Asian | 469 | 35.6% | NR | NR |
| Sugio 2009 | Japan | Clinical trial | Asian | 48 | 33.3% | 85.4% | 22.9% |
| Sun 2010 | China | Case-series | Asian | 301 | 42.2% | 62.5% | 42.5% |
| Sun 2011 | China | Case-series | Asian | 80 | 37.5% | NR | NR |
| Sun 2011 | China | Case-series | Asian | 316 | 33.5% | 65.8% | 51.9% |
| Sun 2012 | China | Cohort study | Asian | 150 | 26.7% | NR | NR |
| Sun 2012 | Korea | Case-series | Asian | 382 | 54.2% | 93.7% | 61.5% |
| Sun 2013 | Korea | Cohort study | Asian | 484 | 42.4% | NR | NR |
| Suzuki 2005 | Japan | Case-series | Asian | 130 | 33.8% | NR | NR |
| Suzuki 2005 | Japan | Case-series | Asian | 150 | 32.7% | 52.7% | 27.3% |
| Suzuki 2007 | Japan | Cohort study | Asian | 238 | 29.4% | NR | NR |
| Suzuki 2010 | Japan | Cohort study | Asian | 229 | 30.1% | 57.6% | 29.3% |
| Taga 2012 | U.S. | Case-series | Caucasian | 143 | 100.0% | NR | NR |
| Takahashi 2009 | Japan | Case-series | Asian | 313 | NR | NR | NR |
| Takamochi 2011 | Japan | Cohort study | Asian | 189 | NR | 88.4% | NR |
| Takano 2005 | Japan | Cohort study | Asian | 66 | 39.4% | 93.9% | 47.0% |
| Takeda 2012 | Japan | Cohort study | Asian | 200 | 36.5% | NR | NR |
| Takeshita 2010 | Japan | Case-series | Asian | 69 | 29.0% | NR | NR |
| Tam 2006 | China | Case-series | Asian | 215 | 60.9% | NR | NR |
| Tanaka 2010 | Japan | Case-series | Asian | 308 | 32.8% | 46.1% | 31.2% |
| Taron 2005 | Japan, China, Spain, German, North America, Hong Kong | Case-series | Mixed | 68 | 33.8% | 69.1% | 32.4% |
| Tiseoa 2009 | Italy | Cohort study | Caucasian | 63 | 38.5% | 76.9% | 22.0% |
| Tochigi 2011 | U.S. | Case-series | Unclear | 23 | 30.4% | NR | NR |
| Togashi 2010 | Japan | Case-series | Asian | 55 | 56.4% | NR | NR |
| Tokumo 2005 | Japan | Case-series | Asian | 120 | 30.8% | 68.3% | 30.0% |
| Tomizawa 2005 | Japan | Case-series | Asian | 120 | 35.8% | NR | NR |
| Tommaso 2011 | Italy | Case-series | Caucasian | 681 | NR | NR | NR |
| Toyooka 2007 | Japan, U.S., Taiwan and Australia | Case-series | Mixed | 1467 | 37.6% | 68.9% | 36.8% |
| Toyooka 2008 | Japan | Case-series | Asian | 262 | 44.8% | NR | 64.9% |
| Treball 2011 | Palestine | Case-series | Unclear | 71 | 50.7% | NR | NR |
| Tsai 2012 | Taiwan | Cohort study | Asian | 78 | 59.0% | NR | 70.5% |
| Tsao 2005 | Multiple | Clinical trial | Mixed | 177 | 37.3% | 13.0% | 5.7% |
| Tsao 2006 | U.S. | Case-series | Caucasian | 159 | 46.5% | 56.0% | 14.5% |
| Tsao 2011 | Canada | Case-series | Caucasian | 436 | 34.2% | 53.0% | 0.5% |
| Ueno 2012 | Japan | Case-series | Asian | 1262 | 39.1% | NR | NR |
| Ulivi 2012 | Italy | Case-series | Caucasian | 32 | 28.1% | NR | NR |
| Umemuraa 2012 | Japan | Cohort study | Asian | 44 | 48.4% | 85.6% | 43.3% |
| Uramoto 2005 | Japan | Cohort study | Asian | 20 | 45.0% | NR | NR |
| Uramoto 2010 | Japan | Cohort study | Asian | 437 | 43.5% | NR | NR |
| U.S.ui 2011 | Japan | Case-series | Asian | 138 | 43.4% | 85.4% | 37.4% |
| U.S.ui 2011 | Japan | Cohort study | Asian | 198 | 43.4% | NR | NR |
| van Zandwijk 2007 | Netherlands | Cohort study | Caucasian | 41 | NR | NR | NR |
| Varghese 2013 | U.S. | Case-series | Mixed | 852 | 57.7% | NR | NR |
| Vincenten 2012 | Netherlands | Case-series | Caucasian | 754 | 49.2% | NR | NR |
| Wada 2012 | Japan | Clinical trial | Asian | 27 | 26.7% | 86.7% | 16.7% |
| Wang 2008 | China | Case-series | Asian | 24 | 41.7% | 75.0% | 62.5% |
| Wang 2010 | China | Cohort study | Asian | 19 | 47.4% | NR | NR |
| Wang 2011 | China | Case-series | Asian | 31 | 9.7% | 38.7% | 29.0% |
| Wang 2011 | China | Case-series | Asian | 184 | 42.4% | NR | NR |
| Wang 2011 | China | Case-series | Asian | 1410 | 37.0% | NR | NR |
| Wang 2012 | China | Cohort study | Asian | 205 | 51.7% | NR | NR |
| Wang 2013 | China | Case-series | Asian | 66 | 24.2% | 40.9% | 51.5% |
| Webb 2008 | Australia | Case-series | Caucasian | 53 | 75.5% | NR | NR |
| Welsh 2013 | U.S. | Clinical trial | Mixed | 17 | 52.9% | 100.0% | 35.3% |
| Wen 2012 | China | Case-series | Asian | 70 | 41.4% | 64.3% | 45.7% |
| Wong 2009 | China | Case-series | Asian | 266 | 50.4% | 78.6% | 53.0% |
| Wu 2007 | Taiwan | Case-series | Asian | 91 | 44.0% | NR | NR |
| Wu 2007 | Taiwan | Case-series | Asian | 136 | 61.0% | NR | NR |
| Wu 2007 | China | Cohort study | Asian | 506 | 35.0% | 54.0% | 41.3% |
| Wu 2008 | Taiwan | Case-series | Asian | 235 | 41.7% | 66.8% | 49.4% |
| Wu 2008 | Taiwan | Cohort study | Asian | 328 | 65.5% | NR | 77.4% |
| Wu 2009 | Taiwan | Cohort study | Asian | 95 | 73.7% | NR | NR |
| Wu 2010 | Taiwan | Cohort study | Asian | 50 | 66.0% | 96.0% | 84.0% |
| Wu 2010 | China | Cohort study | Asian | 145 | 45.5% | 73.1% | NR |
| Wu 2010 | Taiwan | Cohort study | Asian | 156 | 60.9% | NR | NR |
| Wu 2011 | Taiwan | Case-series | Asian | 327 | 47.7% | 40.8% | 28.9% |
| Wu 2011 | Taiwan | Cohort study | Asian | 466 | 64.6% | 92.7% | 77.0% |
| Wu 2011 | Taiwan | Cohort study | Asian | 1261 | NR | 84.9% | 34.0% |
| Xu 2006 | China | Case-series | Asian | 135 | 30.4% | 55.6% | 40.0% |
| Xu 2007 | China | Case-series | Asian | 66 | 51.5% | 65.2% | 51.5% |
| Xu 2009 | China | Cohort study | Asian | 106 | 48.1% | 73.6% | 67.0% |
| Xu 2012 | China | Case-series | Asian | 861 | 42.7% | NR | NR |
| Yamada 2011 | Japan | Case-series | Asian | 122 | 52.1% | NR | NR |
| Yamaguchi 2012 | Japan | Case-series | Asian | 68 | NR | NR | NR |
| Yang 2004 | U.S. | Case-series | Mixed | 219 | 38.8% | NR | NR |
| Yang 2008 | Taiwan | Clinical trial | Asian | 90 | 65.6% | 91.1% | 73.3% |
| Yang 2009 | China | Case-series | Asian | 180 | 48.0% | NR | NR |
| Yang 2010 | China | Clinical trial | Asian | 62 | 64.0% | 82.7% | 84.0% |
| Yang 2011 | China | Case-series | Asian | 231 | 46.2% | NR | 64.1% |
| Yang 2011 | China | Case-series | Asian | 617 | 50.7% | NR | NR |
| Yang 2012 | Taiwan and U.S. | Clinical trial | Mixed | 386 | NR | NR | NR |
| Yin 2010 | China | Cohort study | Asian | 107 | 23.4% | NR | NR |
| Yip 2013 | Australia | Cohort study | Caucasian | 204 | 41.2% | NR | NR |
| Yoshida 2007 | Japan | Cohort study | Asian | 66 | 45.5% | 89.4% | 36.4% |
| Yoshida 2010 | Japan | Cohort study | Asian | 100 | 47.0% | 95.0% | 43.0% |
| Yoshimasu 2011 | Japan | Case-series | Asian | 46 | 43.5% | NR | NR |
| Yu 2012 | China | Case-series | Asian | 20 | 45.0% | NR | NR |
| Zhang 2005 | China | Case-series | Asian | 30 | 46.7% | 21.4% | NR |
| Zhang 2007 | China | Case-series | Asian | 145 | NR | NR | NR |
| Zhang 2008 | China | Case-series | Asian | 59 | 47.5% | 74.6% | NR |
| Zhang 2008 | China | Case-series | Asian | 82 | 41.5% | 47.6% | 51.2% |
| Zhang 2011 | China | Case-series | Asian | 454 | 43.6% | NR | NR |
| Zhang 2012 | China | Case-series | Asian | 205 | 32.7% | NR | NR |
| Zhao 2007 | China | Case-series | Asian | 75 | 40.0% | 46.7% | NR |
| Zhao 2009 | China | Clinical trial | Asian | 27 | 31.8% | 70.5% | 43.2% |
| Zhao 2011 | China | Case-series | Asian | 192 | 40.1% | NR | NR |
| Zhao 2012 | China | Case-series | Asian | 111 | 31.5% | NR | NR |
| Zhong 2009 | China | Case-series | Asian | 17 | 64.7% | NR | NR |
| Zhong 2012 | China | Case-series | Asian | 160 | 49.4% | 89.4% | NR |
| Zhou 2007 | China | Case-series | Asian | 80 | 36.3% | 47.5% | NR |
| Zhou 2011 | China | Case-series | Asian | 100 | 51.0% | 93.0% | NR |
| Zhou 2012 | China | Case-series | Asian | 102 | 47.1% | 70.6% | 51.0% |
| Zhu 2008 | Canada | Clinical trial | Mixed | 204 | 35.8% | 14.6% | NR |
| Zhu 2010 | China | Case-series | Asian | 17 | 36.7% | 88.6% | NR |
| Zhu 2012 | China | Cohort study | Asian | 155 | 52.3% | NR | NR |
| Zhuang 2011 | China | Case-series | Asian | 43 | 62.8% | NR | NR |
| Zhuo 2011 | China | Case-series | Asian | 145 | 45.5% | 73.1% | NR |
| Zimmer 2007 | Germany | Case-series | Caucasian | 67 | 35.8% | NR | NR |

*Not reported.
